# Comparison of contamination by polycyclic aromatic hydrocarbons, pesticides and pharmaceuticals in abandoned meanders and channel bars, Czech Republic

**DOI:** 10.1007/s10661-025-14928-0

**Published:** 2025-12-24

**Authors:** Jan Sedláček, Jitka Tolaszová, Zuzana Lenďáková, Ondřej Bábek, Ondřej Koukal, Lukáš Maloušek

**Affiliations:** 1 Department of Geology, Palacký University of Olomouc, Tř. 17 Listopadu 12, 771 46 Olomouc, Czech Republic; 2 Faculty of Environment, J. E. Purkyně University, Králova Výšina 3132/7, 400 96, Ústí Nad Labem, Czech Republic

**Keywords:** Channel bars, Abandoned meanders, Sedimentary archives, Polycyclic aromatic hydrocarbons, Pesticides, Pharmaceuticals

## Abstract

This article focuses on the necessity to enhance the current understanding of the accumulation and fate of pesticides and pharmaceuticals (emerging pollutants) in abandoned meanders and adjacent river channel bars. The primary objective of this study is to conduct a comparative analysis of pollutant concentrations in both settings and to identify the driving factors of their deposition. The studied sites are situated within two distinct catchments of the Morava and Odra rivers in the eastern part of the Czech Republic. The most prevalent pesticides were identified as propiconazole, metazachlor and tebuconazole. For caffeine and pharmaceuticals, the peak concentrations exceeded 10 µg/kg. The other pharmaceuticals frequently detected in these sediments were carbamazepine, diclofenac and metoprolol. The polycyclic aromatic hydrocarbons and polychlorinated biphenyls, selected for comparison (hereafter referred to as ‘legacy pollutants’), reached high to extremely high levels due to their frequent use in the past or present. Pollutant assemblages differed between channel bars and abandoned meanders. The maximum concentrations of emerging pollutants have been observed to be generally higher in abandoned meanders; however, the relative occurrence of pollutants has been found to be higher in channel bars. Abandoned meanders are most vulnerable to contamination in the first years following the cut-off, due to the increased frequency of flooding during this period. Consequently, they pose a higher environmental risk. Older meanders contain residual concentrations of pollutants and can serve as long-term sinks for organic pollutants, thereby providing temporal patterns. Conversely, channel bars represent current contamination levels and thus indicate spatial trends.

**Supplementary Information:**

The online version contains supplementary material available at 10.1007/s10661-025-14928-0.

## Introduction

The impact of human activities on modern fluvial systems is well documented (e.g., Brázdil et al., 2011; Charlton, 2008), and rivers have been identified as significant transport vectors of contaminated sediments. Oxbow lakes (water bodies) and abandoned meanders (terrestrialised), as common landforms of meandering river floodplains, are often located in lowland areas and serve as ideal traps for suspended river sediments. The majority of these are located in regulated river courses, behind flood defences, and experience limited flooding (Hudson et al., 2008; Toonen et al., 2012). Naturally formed oxbow lakes are rare; frequent flooding in natural river segments provides a high-resolution sedimentary record that includes contamination (Sedláček et al., 2019, 2020). Furthermore, the contamination of oxbow lakes has been identified as a significant concern (Ciazela et al., 2018). This makes them a logical choice for a range of contamination studies (e.g., Bábek et al., 2008, 2011; Ciazela et al., 2018; Falkowska and Falkowski, 2015; Nguyen et al., 2009; Zablotowicz et al., 2010; Zachmann et al., 2013). During the twentieth century, many new and potentially harmful persistent organic pollutants (POPs) were introduced into the environment, including polycyclic aromatic hydrocarbons (PAHs) and polychlorinated biphenyls (PCBs). PCBs were banned, but PAHs have natural and anthropogenic ongoing sources. The concentrations of numerous legacy compounds in sedimentary fluvial archives have diminished over the past few decades. This decline can be attributed to the implementation of enhanced environmental legislation and the decline of industry in post-communist countries (e.g., Bábek et al., 2008; Matys Grygar et al., 2012, 2018). On the other hand, other organic pollutants (emerging pollutants) such as pesticides and pharmaceuticals are still used extensively, resulting in the widespread loading of the aquatic environment by these compounds (Kunkel & Radke, 2012). Although the half-lives of currently used pesticides are generally shorter than those of the formerly used organochlorinated pesticides, repeated use can lead to their gradual accumulation in the environment, as their degradation rates are slower than their input (Hvězdová et al., 2018). Pesticides are gradually and consistently released from the soil, thereby allowing the presence of compounds that have been prohibited for extended periods to be identified within the environment (Barchanska et al., 2017; Chiaia-Hernandez et al., 2017; Fikarová et al., 2018). Sheet washing and erosion are the primary mechanisms that cause their removal, transport via fluvial systems and subsequent possible deposition in sedimentary traps such as river channel sediments (Radović et al., 2015; Schulz, 2004), floodplain sediments (Karlsson et al., 2020; Masiá et al., 2013) or pond sediments (Chaumet et al. 2021). A plethora of studies have been dedicated to determining their concentrations in arable soils worldwide (e.g., Hvězdová et al., 2018; Park et al., 2013; Plaza-Bolanos et al., 2012; Sharma et al., 2014); however, they have rarely been measured in floodplain sediments (e.g., Fikarová et al., 2018; Karlsson et al., 2020; Witter et al., 2003). Nevertheless, little is still known about their occurrence in abandoned meanders and oxbow lakes. It has been demonstrated that pharmaceutical residues are not fully eliminated by sewage treatment plants, and they occur with concentrations comparable to those of pesticides (Löffler et al., 2005).

A plethora of studies have previously investigated the presence of pharmaceuticals in diverse aquatic environments, including river water and channel sediments (e.g., Feitosa-Felizzola & Chiron, 2009; Hanamoto et al., 2018; Kondor et al., 2022), estuaries (Kucharski et al., 2022), ponds (Koba et al., 2018), lakes (Blair et al., 2013; Kerrigan et al., 2018; Xu et al., 2014) or floodplains (Matongo et al., 2015). However, the occurrence of pharmaceuticals in abandoned meanders remains unexplored. In river channels, sediments are deposited under lower current-velocity conditions to form mid-channel bars, side-channel bars, and point bars (Miall, 2006). Given their nature, channel bars cannot provide a meaningful stratigraphic record; instead, they can reflect current pollution sources in their catchments (e.g., Faměra et al., 2013; Nehyba et al., 2010). Organic pollutants are assumed to have higher concentrations in the fine-grained sediments of abandoned meanders compared to channel sediments; however, no study has directly tested this assumption. To our knowledge, no study has yet compared the accumulation of pesticides and pharmaceuticals in abandoned meanders versus channel bars.

This study aims to fill that gap by determining to what extent emerging pollutants can accumulate in abandoned meanders and how their levels are affected by fluvial settings and sedimentation conditions. The objective of this study is to provide insight into the occurrence, concentrations and distribution of selected pesticides and pharmaceuticals in abandoned meanders. The analysed compounds were selected based on analytical feasibility and their past or current use in the Czech Republic. Pesticides and their transformation products, determined in sediment samples, were selected from all substances approved for plant protection products in the Czech Republic that are currently in use or were used until recently and persist in the environment. Substances with total annual consumption in the Czech Republic exceeding 5 tons were considered. Pharmaceuticals detected in sediment samples were selected from all substances widely used in the Czech Republic, based on monitoring by the National Institute of Public Health. The findings will be compared with data from adjacent channel bars, reflecting current pollution loads. The investigation extends to other organic pollutants, including PAHs and PCBs, to facilitate a comparative analysis of pollution impacts. This approach is motivated by the recognition that both groups are representative of typical legacy compounds and by their ability to persist in the environment for long periods. A further objective is to undertake a comparative analysis of organic pollutant concentrations across two distinct catchments, followed by a discussion of potential disparities between them.

## Geographic settings

**Fig. 1:**
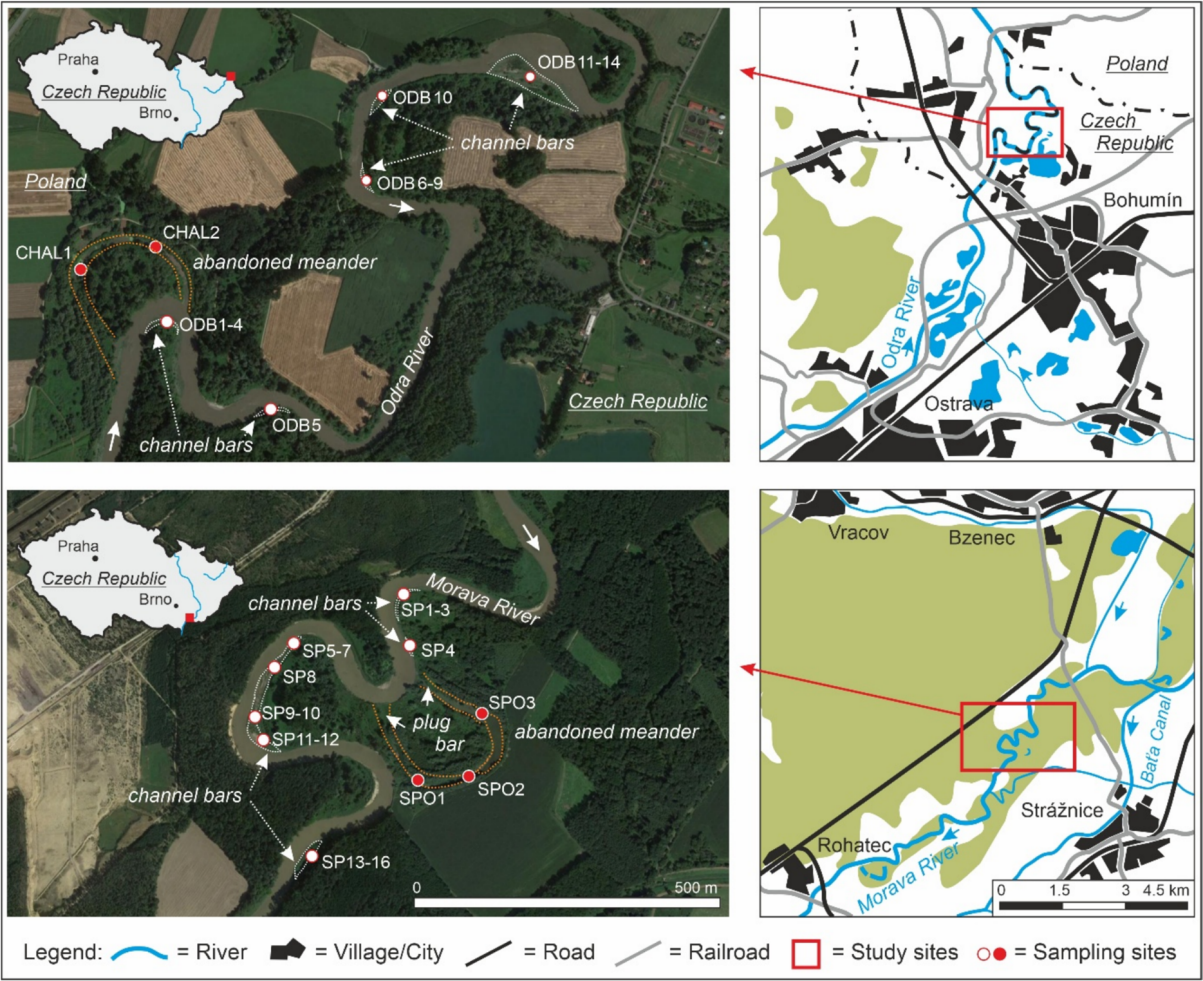
Geographic context, location of sampling sites (channel bars) and sediment cores (abandoned meanders)

For this study, we selected two short semi-natural meandering river segments of the two most important Czech rivers, the Morava and Odra rivers, in the eastern part of the Czech Republic (Fig. 1), representing some of the few semi-natural to natural river courses with considerable river dynamics, with an active meandering process. The positions of the studied sites within natural river segments, along with the absence of flood defences and dykes, enable relatively frequent flooding. In addition, the locations of the studied sites in lower river reaches enable the collection of regional pollution data, thereby enabling effective monitoring of pollution levels. The first site comprises a protected river reach of the Odra River in the north-eastern part of the Czech Republic, at the border between the Czech Republic and Poland, and between the cities of Bohumín (Czech Republic) and Chałupki (Poland). The Odra River in the Czech Republic is 131.7 km long (total length, 854 km). The mean annual discharge of the Odra River is 48.07 m^3^s^−1^ (Bohumín gauging station); the value of Q_100_ is 1810 m^3^s^−1^. The historic maxima of the river discharge were recorded during the devastating floods in 1985 (732 m^3^s^−1^), 1997 (2160 m^3^s^−1^) and 2010 (1067 m^3^s^−1^). From its source, the Odra River flows through an agricultural landscape and several settlements. The large, densely populated urban agglomeration (about one million inhabitants) of the city of Ostrava and the nearby cities of Havířov, Karviná and Bohumín is located just a few km upstream of the studied site. This region was known as the so-called Steel Heart of former Czechoslovakia, a highly industrialised area impacted by coal mining, as well as the chemical industry, metallurgy and machine engineering. Industrial production and coal mining peaked in the 1980 s; this was followed by industrial decline and the closure of some factories after 1989 (Chudaničová et al., 2016; Mulková et al., 2016).

The second study area is located in the south-eastern part of the Czech Republic within the Strážnické Pomoraví protected landscape area on the Morava River. The Morava River is 353.1 km long, and its catchment covers 26,578 km^2^. The mean annual discharge is 59.6 m^3^s^−1^ at the gauging station in Strážnice. The value of Q_100_ is 790 m^3^s^−1^. The peak discharge, reaching a value of 901 m^3^s^−1^, was recorded in July 1997. Lower discharges were observed during floods in 2010 (754 m^3^s^−1^) and 2006 (733 m^3^s^−1^). The Morava River catchment area is used for agriculture, with a large proportion of arable land, followed by forest and grassland areas (Brázdil et al., 2011). Industrial activities are concentrated near large- and medium-sized towns, such as Olomouc, Zlín, Otrokovice, and Uherské Hradiště, located upstream of the sampling area.

## Methods

The channel and point bar samples collected above water were obtained directly in situ (with a minimum weight of 20 g) and wrapped in aluminium foil. Five cores were retrieved from terrestrialised parts of the abandoned meanders using a portable groove corer with an inner diameter of 3 cm (Eijkelkamp, the Netherlands). In the Odra River in the Bohumín area, the channel bar samples were denoted as ODB1 to ODB14, and the samples from the abandoned meander were denoted as CHAL1 and CHAL2 (see Fig. 1). Samples obtained from the Morava River in the Strážnice area were denoted as SP1 to SP14 for channel bars and SPO1 to SPO3 for abandoned meander cores (see Fig. 1). The cores were sampled directly in the situ at a vertical interval of 2 cm or 5 cm (cores SPO2 and CHAL2). Following collection, a selection of samples was immediately transported to the laboratory and lyophilised using a Scanvac lyophiliser (Trigon Plus, Czech Republic) prior to the analysis of organic pollutants.

The grain-size analysis of selected samples was performed by wet-suspension laser granulometry using a Fritsch Analysette 22 MictoTec Plus laser particle sizer (Fritsch, Germany) in the range 0.08–2000 µm. The disintegration of particle aggregates was performed in the ultrasound dispersion unit of the granulometer. The total organic carbon (TOC) was measured in a selection of samples. Prior to analysis, the samples (SPO1, SPO4, and CHAL3 cores) were processed by HCl decomposition, and TOC was subsequently measured using an Eltra 1000CS device (Eltra, Germany). For other samples (channel bar sediments, CHAL2 and SPO3 cores), TOC analysis was performed using a Skalar Analytical TOC analyser (Breda, the Netherlands). The TOC was calculated as the difference between the total carbon (TC) and the inorganic carbon (IC). The TC was determined after catalytic oxidation of the sample (300 mg) at 1100 °C, and the proportion of inorganic carbon was determined by acidifying the sample with 20% H_3_PO_4_ at 150 °C. CaCO_3_ was used to calibrate the instruments.

For the analysis of organic pollutants, all channel bar samples were used; two cores from abandoned meanders in the most distal sites were also used because of the expected fine-grained nature of the sediments. Pre-treatment of PAHs, PCBs, pesticides, and pharmaceuticals was conducted in accordance with the previously delineated protocol (Fikarová et al., 2018). Further details pertaining to this protocol can be found in Supplementary Material 1. For pesticides and pharmaceuticals, methanol (Honeywell, USA) and water (Merck Millipore, Germany) were used as solvents. PAHs and PCBs were extracted by QuEChERS with ethyl acetate (Chromservis, Czech Republic), water (Merck Millipore, Germany), MgSO4 (Merck Millipore, Germany) and NaCl (Penta, Czech Republic). Vortex Mix (Chromservis, Czech Republic) was also used for extraction. A centrifuge (Remi X5 R-10 M, Chromservis, Czech Republic and Biosan Microspin 12, Merci, Czech Republic) was used to centrifuge sample extracts. Nylon syringe filters (0.22 μm, Chromservis, Czech Republic) were added to filter the extracts. An Rxi PAH column (40 m × 0.18 mm, 0.07 μm) (Restek, USA) was used for separation and a DB-EUPAH column (20 m × 0.18 mm, 0.14 μm) (Agilent Technologies, USA) was used for PAH and PCB determination. Sediment extracts were analysed on a gas chromatograph (7890B, Agilent Technologies, USA) coupled to a mass spectrometer (7000D triple quadrupole, Agilent Technologies, USA). Data evaluation was performed in the MassHunter software (version B.09.00) from Agilent Technologies. For pesticides and pharmaceuticals, the extracts were analysed on LC-MS/MS. Gradient elution was used for chromatographic analysis; the time course is described in Supplementary Material 1. Water containing 0.5 mM NH_4_F and 0.25 mM CH_3_COONH_4_ and methanol containing 0.25 mM NH_4_F were used as the mobile phases. The determination was performed on a Kinetex column (2.6 μm C18 100 Å 150 × 2.1 mm). The temperature in the column was 40 °C. The sample injection on the column was 20 µl. The following characteristics were studied: selectivity, linearity, detection and quantification limits (LOD and LOQ), repeatability, recovery, accuracy based on reference material (if available) and matrix effect. Selectivity was ensured using MRM (multiple reaction monitoring) transitions. The limit of detection (LOD) and the limit of quantification (LOQ) were determined as three times or ten times the standard deviation of the signal of the standard solution at the lowest standard concentration in the sediment. The LOQ of all analytes are listed in Supplementary Material 1. These limits were verified using repeated measurements of standards (*n* = 7) or spiked extracts (*n* = 7) at concentrations close to the LOQ and at higher levels. For testing linearity, linear regression with 9 concentration levels was used. Repeatability was examined using the same instrument, the same operator and six repetitions; overall repeatability was calculated from the average of the entire procedure, from sediment weighing through extraction, to the prepared extract solution for analysis of enriched sediments, expressed as a relative standard deviation. Recoveries were calculated as the percentage of the analysed signal in the sample solution compared to the standard solution of the same concentration. A complete description of the methodology is provided in Supplementary Material 1 (Tables S1-S6).

## Results and discussion

### Evolution of the studied abandoned meanders and channel bars

**Fig. 2:**
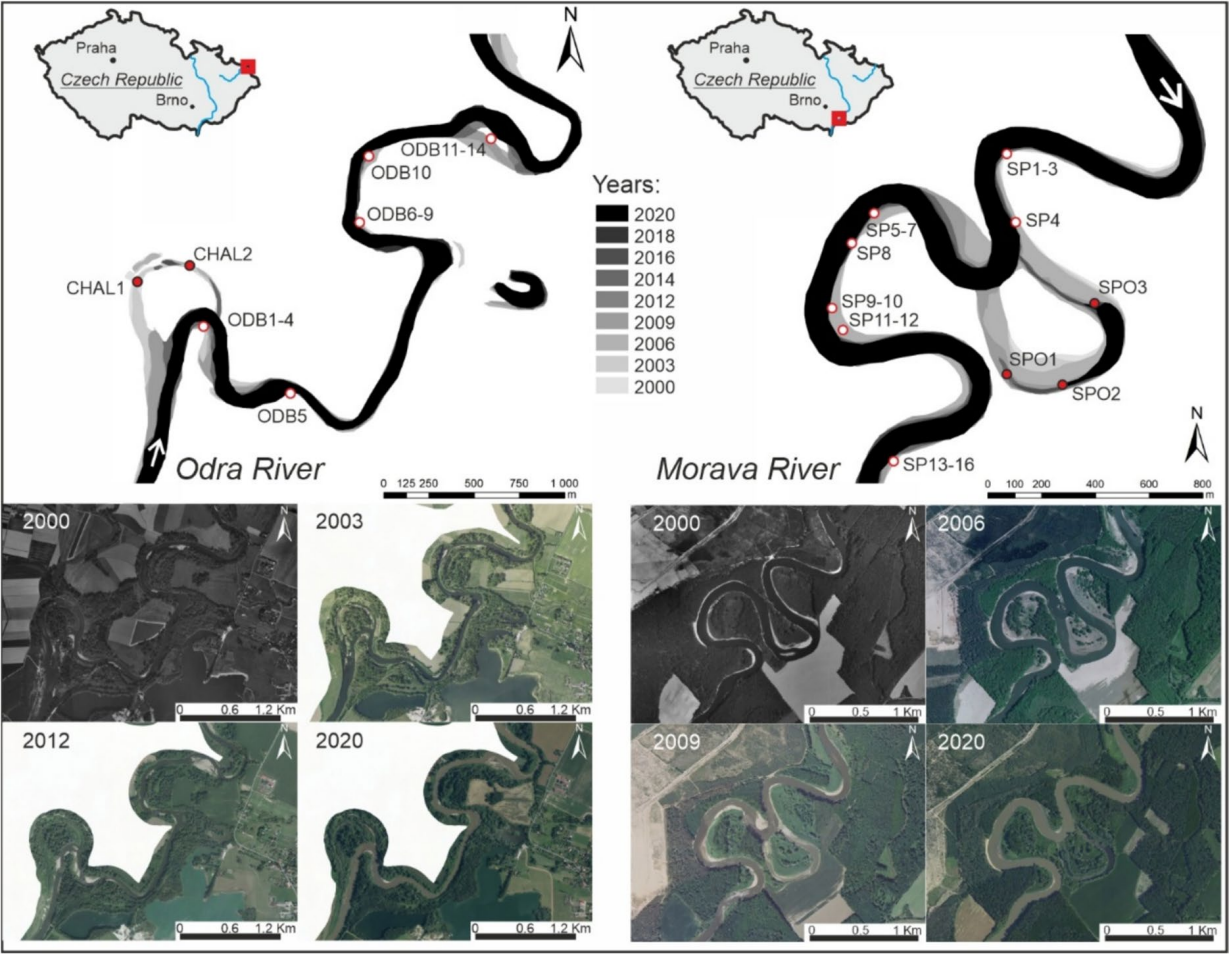
Evolution of the study sites within the natural river reaches of the Odra River (Bohumín area) and the Morava River (Strážnické Pomoraví area) for the period 2000 to 2020, as well as major channel shifts

Both localities exhibited strong fluvial processes and underwent rapid evolution (Fig. 2). Therefore, short-term river dynamics and abandoned meander development were evaluated by comparing a set of historical aerial maps for the period 2000–2020, which revealed significant changes in the river channel. The Odra River segment near Bohumín experienced a substantial channel shift following the 1997 flood, resulting in a cut-off event (Kasperek, 2015). The old and new channels of the Odra River coexisted for several years after the cut-off, and no plug bar formed. Consequently, the abandoned meander was quickly filled with sediment, leading to rapid narrowing and shallowing. At present (autumn 2025), only a minor water body remains in the central area. Channel-side bars and point bars in the adjacent Odra River reach exhibited lateral growth. Especially small channel bars experienced modifications depending on the actual discharge. In the Strážnické Pomoraví Morava River segment, the 2006 flood caused a meander cut-off (Máčka and Kadlec, 2016), resulting in channel shortening and the creation of a newly abandoned meander (Fig. 2). The inflow and outflow parts were quickly filled with sediment, leading to the formation of the alluvial plug. The residual oxbow lake formed in the central part of the former meander, while a heavy sediment supply led to a rapid reduction in its water surface. As of autumn 2025, the residual lake still covers a smaller portion of this abandoned meander, while its bigger portion has been terrestrialised. Channel bars and point bars in the same Morava River segment experienced modifications. The short-term dynamics of channel bars revealed significant lateral erosion on the outer cut banks of the meandering river, as well as the lateral growth of point bars on the opposite inner banks.

### Lithology and grain size distribution

Narrow (1–3 m) channel side bars, attached to the riverbanks, were elongated in the direction of flow in the Strážnice area. Similar characteristics were observed during field campaigns on the Odra River. Based on macroscopic descriptions, the dominant facies comprised river gravel (F1). In the Strážnice area, sandy facies (F2) comprised medium-grained light grey or brown sands with occasionally present organic residues and gravel-sized clasts. The F3 facies is composed of sandy silts, with silt predominating over clay, forming very thin, spatially limited layers. Clayey silts (F4 facies) are well-sorted and form very thin layers in low-velocity zones, allowing the accumulation of fine-grained material.

The thickness of the abandoned meander infill varies from 150 to 215 cm in the Strážnice area (SPO1 to SPO3 cores) and from 100 to 150 cm in the Bohumín area (CHAL1 and CHAL2 cores). The underlying unit, comprising the facies of the former channel bed, was not attained in the cores. Nevertheless, we anticipate that, as in other studies, the depth position of this boundary was revealed by resistance to coring penetration (Citterio & Piégay, 2009; Constantine et al., 2010; Sedláček et al., 2019). In the Strážnické Pomoraví area, the sediments of the abandoned meander infill consist of homogeneous dark grey or brown silts, sandy silts and sometimes sands or silty sands. The proximal cores recovered closer to the Morava River exhibit a coarser-grained texture than the distal core. Small coal particles, identified under a binocular microscope, were found in the basal strata of the Bohumín abandoned meander, as well as in some samples from adjacent channel bars. However, the relative abundance of these particles was lower than in other studies (Geršlová & Schwarzbauer, 2014; Sedláček et al., 2020) because the sediments in this study are younger, deposited after the decline of coal mining in the Ostrava region. The sediment infill of the distal part of the abandoned meander consists of grey-brown silty sands and poorly sorted medium- to coarse-grained sands (basal layers), and the top layers are composed of homogeneous brown-grey silts. The proximal sediments are composed of brown, very poorly sorted medium- to coarse-grained sands and rarely silty sands.

**Fig. 3:**
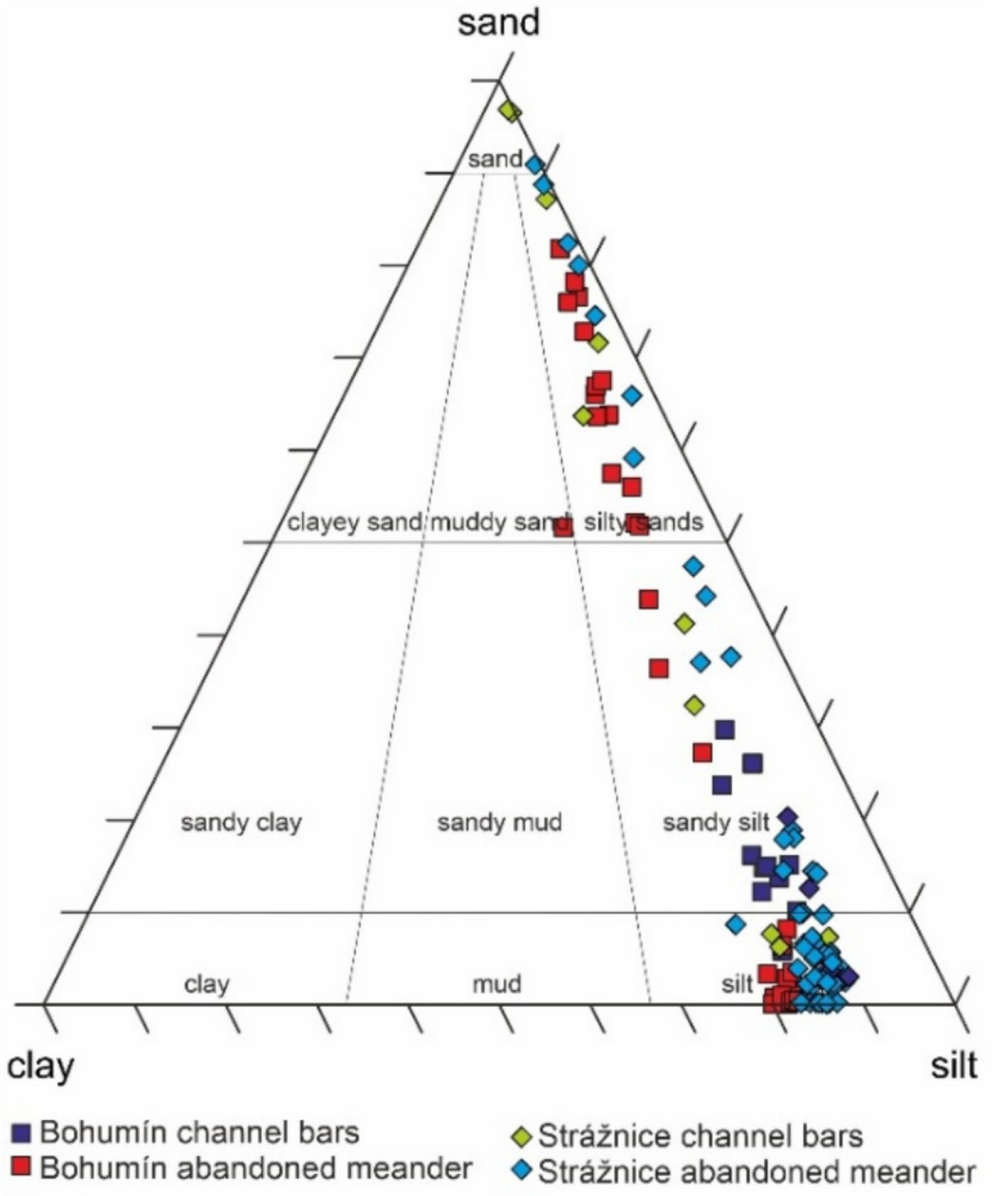
Ternary plot diagram showing clay, silt and sand proportions

**Fig. 4:**
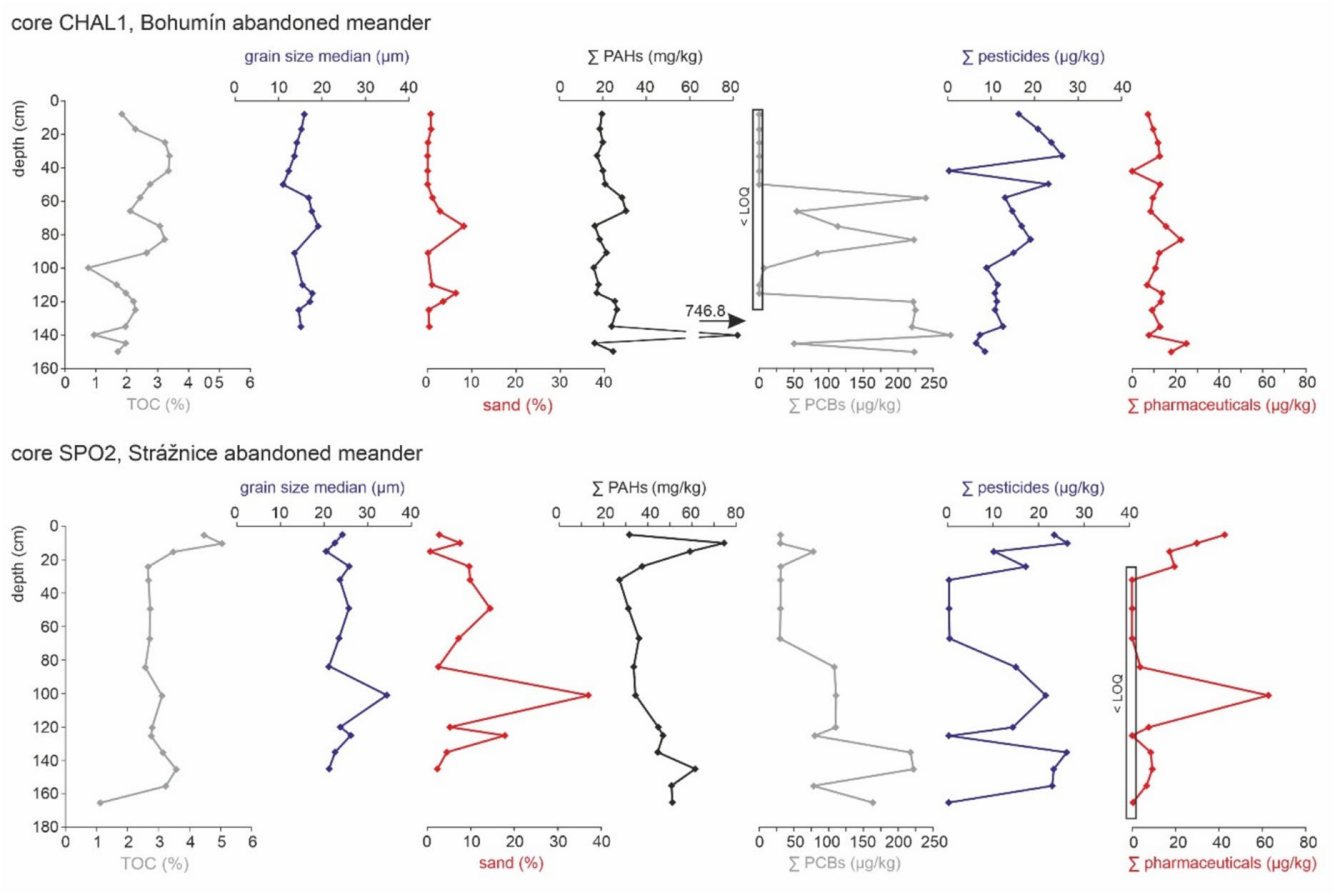
Depth distribution of total organic carbon (TOC), grain size median, sand percentage, the sum of 16 PAHs, the sum of six PCBs congeners, the sum of pesticides and pharmaceuticals in two cores from two abandoned meanders (Bohumín area and Strážnické Pomoraví area)

Grain-size analysis showed a highly variable distribution (Table 1 and Supplementary Material 2, Table S7); however, silt and sand predominated in most samples, followed by clay. According to the ternary plot (Fig. 3), most samples are classified as silts, sandy silts or silty sands (Folk’s classification, 1974). Differences in the grain-size distribution were observed between channel bars and abandoned meander fills, while in the latter settings, grain size varied in the proximal-to-distal direction. Downcore grain-size changes were typical for deeper parts of the cores from abandoned meanders (Fig. 4). In the upper parts of the cores, the depth pattern was free of fluctuations, indicating almost stable flow conditions during deposition. The upward grain-size reduction indicates an upward fining trend. Silt predominated in most of the samples. Both abandoned meanders displayed higher median silt values, ranging from 74.8 to 84.2% in the Strážnice abandoned meander and reaching 80.5% in the Bohumín abandoned meander, except for its proximal part, which showed a median value of only 33.6%. Slightly lower silt proportions were recorded in the Bohumín channel bars, which had a median value of 71.9%, and much lower proportions were recorded in the Strážnice channel bars, which were 49.7% silt. The clay content was relatively low in the channel side bars, especially in the Strážnice area, with a median of 8.9% (min. 0.3% and max. 16.2%). Slightly higher values were observed in the Bohumín area, with a median of 13.1% (min. 9% and max. 15.9%). In contrast, the infill in the abandoned meanders displayed higher clay contents, and the median clay proportions were similar in the cores from the Strážnické Pomoraví abandoned meander, ranging from 11.5% (SPO1 core) to 11.7% (SPO3 core). Even higher clay proportions were found in the distal part of the Bohumín abandoned meander, which had a median value of 18.1% (CHAL1 core), while a much lower value was found in its proximal part, which had a median value of 7% (CHAL2 core). The sand proportions were highly variable. The basal layers of all cores from the Strážnice abandoned meander contained a substantial amount of sand, up to 91%, but the sand proportion decreased upward. Similar downcore trends were observed in the Bohumín abandoned meander. The sand proportions also varied in the proximal-to-distal direction; a substantial amount of sand was observed in the proximal parts of abandoned meanders.

**Table 1:** Grain size data for selected cores showing the percentage of fractions and grain size median (*D*_50_)

Sample/core	Median (minimum–maximum)			
	Clay (%)	Silt (%)	Sand (%)	*D*_50_ (µm)
Bohumín channel bars				
ODB1 to ODB14	13.1 (9–15.9)	72 (59.9–78.3)	15 (5.8–29.8)	21.5 (16.9–31.2)
Bohumín abandoned meander				
CHAL1	18.1 (14.2–22.1)	80.5 (77.6–82.7)	0.7 (0–8.2)	15.3 (11.1–19.1)
CHAL2	7.0 (2.3–16.9)	29.5 (15.7–58.7)	63.8 (27.3–81.9)	160.3 (23.5–316.1)
Strážnice channel bars				
SP1 to SP15	9 (0.3–16.2)	49.8 (2.5–83.4)	41.3 (5.9–97)	44.8 (19.7–809.2)
Strážnice abandoned meander				
SPO1	11.5 (8.1–16.7)	84.2 (71.6–87.1)	3.1 (0–20.3)	21.8 (15.6–29.1)
SPO2	13.1 (0.3–22.1)	72 (2.5–87.1)	15 (0–97)	21.8 (11.1–809.2)
SPO3	10.4 (0.6–19.7)	74.9 (8.4–87.2)	11.4 (0–91.1)	22.2 (12–22.7)

### Vertical and spatial patterns of TOC

TOC measurements showed low to medium TOC values (Supplementary Material 2, Table S8). The TOC content tends to increase as the grain size decreases (Sedláček et al., 2019), and this dependence was best documented in channel bars from the Strážnické Pomoraví area, which, on the other hand, showed generally low TOC values. Here, TOC values ranged from 0.1 to 4.2%, with a median value of 1.7%. The coarse-grained facies exhibited TOC concentrations under 1%, while the silt-to-clay facies exhibited the highest TOC values. Generally, higher values were found in the Strážnice abandoned meander, ranging from 0.3 to 5.1%, with median values of 2.6% in the SPO1 core, 5.1% in the SPO2 core and 2.1% in the SPO3 core. Proximal-to-distal trends indicate rising TOC values at distal sites. A gradual vertical increase in TOC values (Fig. 4) was observed in the cores. The TOC values were significantly higher in the Bohumín area in the Odra River channel bars, ranging from 0.5 to 9.1%, with a median value of 5.1%. The TOC values showed greater variability in the Bohumín abandoned meander. Cyclic oscillations were observed in the distal CHAL1 core, with a slightly increasing upward trend. In the proximal CHAL2 core, the TOC values were similar to those of the distal core, except for the basal strata. Higher TOC values can be associated with the presence of coal particles, as confirmed by visual observations (Geršlová & Schwarzbauer, 2014; Sedláček et al., 2020).

Relatively low TOC values in abandoned meanders are associated with their young age (Sedláček et al., 2019). Lower TOC values can be associated with the removal of light organic matter from coarse-sand-rich strata and the degradation of organic matter to CO_2_ in oxygen-saturated waters (Meyers & Teranes, 2001). Higher TOC values could indicate low-energy conditions, allowing the deposition of fine-grained organic material. The gradual vertical increase in the TOC content observed in abandoned meanders also indicates increasing trophic levels in residual lakes with time and increasing sedimentation from allochthonous sources (Sedláček et al., 2019).

### PAHs and PCBs concentrations

The concentrations of all 16 PAHs (Supplementary Material 3, Table S9) exceed the Czech limit for dry soil in most samples (Czech regulation no. 153/2016), which is 1 mg/kg for the sum of all PAHs. The lowest values were observed in the Strážnice channel bars, ranging from 0.1 to 22.8 mg/kg. Higher values were observed in the Bohumín channel bar samples, ranging from 6.2 to 72.7 mg/kg. Generally, PAH concentrations were higher in abandoned meanders, ranging from 27.2 to 74.5 mg/kg in the Strážnice abandoned meander and from 15.8 to 746.8 mg/kg in the Bohumín abandoned meander. In abandoned meanders, the depth distribution of PAHs (Fig. 4) revealed an almost uniform trend, with the PAH concentration decreasing slightly upward, but the maximum values were observed in the subsurface strata of the Strážnice abandoned meander. An almost uniform pattern was also observed in the Bohumín abandoned meander, except for one extreme peak (746.8 mg/kg). To identify pollution emission sources, the ratio of two- and three-ring to four- and six-ring hydrocarbons (low molecular weight/high molecular weight, LMW/HMW) was calculated, revealing distinct differences between the two areas (Supplementary Material 3, Table S3). Values greater than 1, suggesting petrogenic input (oil-based), were indicated in the channel bars in the Bohumín area; they ranged from 1.22 to 1.78. Meanwhile, values less than 1, indicating pyrogenic (combustion-based) input, were typical in the Strážnice area, ranging from 0.01 to 0.03. There was no difference between channel/point bars and abandoned meanders.

The sum of PCB concentrations (six indicator congeners: PCB-28, 52, 101, 138, 153, and 180) showed high variability (Supplementary Material 3, Table S9). The abandoned meanders displayed higher levels compared to channel bar sediments. The PCB levels were usually below the Czech safety limit of 200 μg/kg (Czech regulation no. 257/2009). Exceptions were found in both abandoned meanders; in the Bohumín area, safety limits were exceeded in seven samples, with a maximum value of 278.7 µg/kg. Slightly lower values were found in the abandoned meander in the Strážnické Pomoraví area, where the PCB concentrations in two samples exceeded the Czech safety limit, reaching a maximum value of 221.7 µg/kg. The depth profiles of PCBs revealed similar patterns; generally, the highest concentrations occurred in deeper layers. The uppermost parts of both profiles showed markedly lower PCB concentrations (SPO2 core, Strážnice abandoned meander) or were under the limit of quantification (LOQ) (CHAL1 core, Bohumín abandoned meander). Significantly lower PCB concentrations were found, especially in channel bars in the Strážnické Pomoraví area, with a maximum of 27.1 µg/kg. Here, more than half of the samples revealed PCB concentrations under the LOQ. PCBs were detected in all Bohumín channel-bar samples, with concentrations ranging from 1 to 47.6 µg/kg.

### Pesticides and pharmaceuticals

**Fig. 5:**
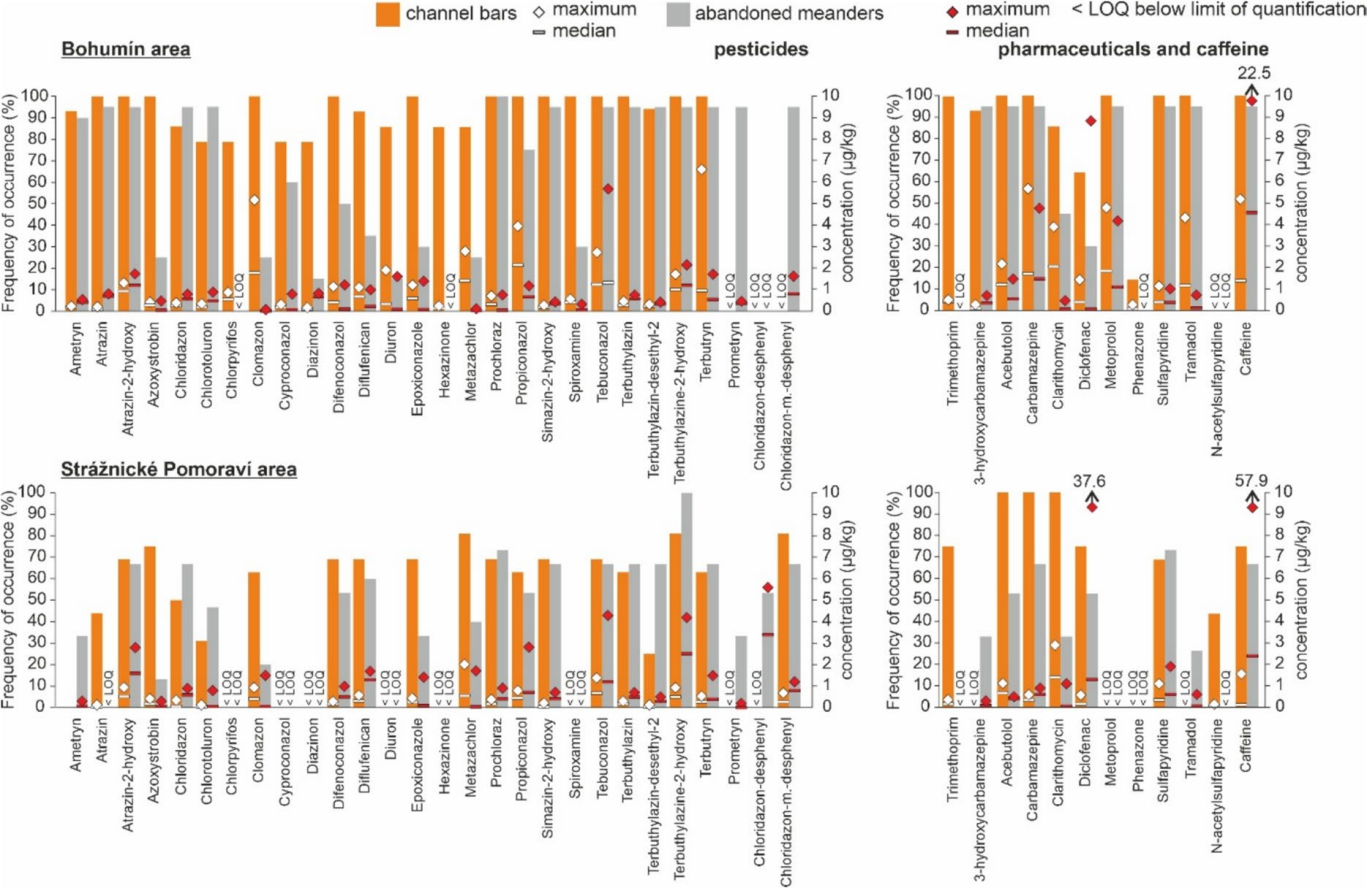
Frequency of occurrence (left Y axis) of individual pesticide and pharmaceutical compounds detected in channel bars (orange columns) and abandoned meanders (grey columns). Median and maximum concentrations (right Y axis) of each compound calculated for the samples where it was detected are shown

The samples contained a mixture of pesticide residues (Fig. 5 and Supplementary Material 3, Table S10), but most were at negligible concentrations; for each pesticide compound, the concentration was predominantly below 1 µg/kg. Most of them are used as fungicides or herbicides. Of the 81 pesticide compounds analysed (see Supplementary Material 1 for the list of all analytes), 29 were detected; the rest were below the LOQ. In general, the most abundant pesticides or pesticide transformation products are propiconazole, metazachlor, tebuconazole and terbuthylazine-2-hydroxy (Fig. 5); in some samples, the concentrations of these substances reached or exceeded 5 µg/kg. The maximum concentrations in the Bohumín area channel bars were found for terbutryn (6.6 µg/kg), clomazone (5.2 µg/kg) and propiconazole (3.9 µg/kg). In the Bohumín abandoned meander, maximum concentrations were observed for tebuconazole (5.7 µg/kg), propiconazole (3.4 µg/kg) and terbuthylazine-2-hydroxy (2.2 µg/kg). In the Strážnické Pomoraví channel bars, the highest concentrations were detected for metazachlor (2 µg/kg), tebuconazole (1.4 µg/kg) and atrazine-2-hydroxy (1 µg/kg). Slightly higher maximum values were found in the Strážnice abandoned meander for chloridazon-desphenyl (5.6 µg/kg), tebuconazole (4.3 µg/kg) and terbuthylazine-2-hydroxy (4.2 µg/kg). The sum of all pesticides varied from 0.3 to 34.7 µg/kg in the Bohumín area and from values below the LOQ to 34.9 µg/kg in the Strážnice area. In general, samples from the Strážnice channel bars revealed the lowest levels of contamination by pesticides, with a mean concentration of 5.4 µg/kg, compared to the Bohumín area channel bars (16.3 µg/kg), Bohumín abandoned meander (12.9 µg/kg) and Strážnice abandoned meander (15 µg/kg).

**Fig. 6:**
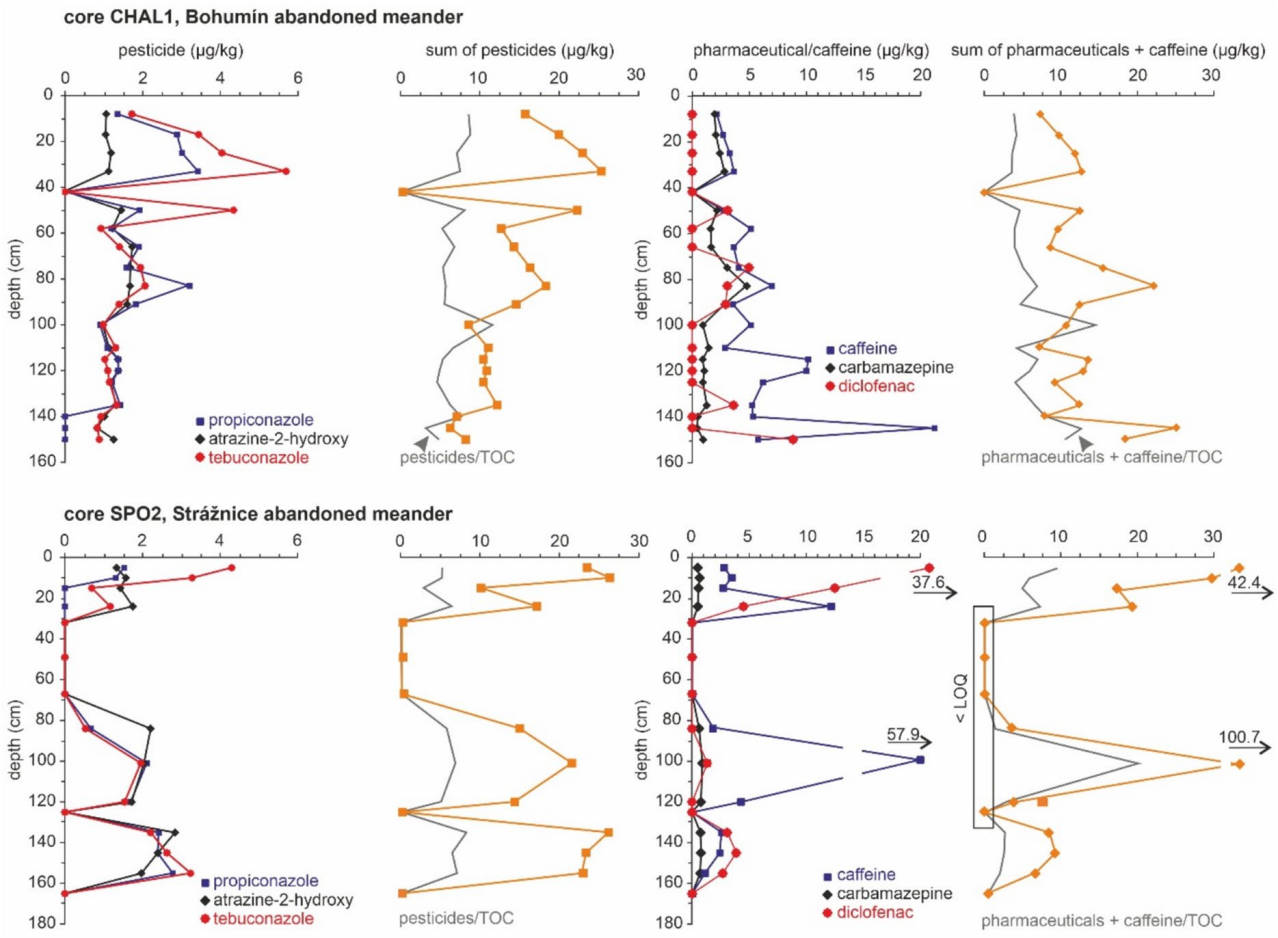
Depth distribution of selected pesticide and pharmaceutical compounds in two cores from two abandoned meanders (Bohumín area and Strážnické Pomoraví area)

The pesticide depth distribution in both cores (CHAL1 and SPO2) from the abandoned meanders shows a saw-tooth pattern (see Fig. 4). Comparing both cores from abandoned meanders showed that almost the entire thickness of the CHAL2 core revealed concentrations from 10 to 26 µg/kg for the sum of all pesticides, with a pronounced minimum at a depth of 42 cm under the surface, while the depth distribution for the sum of all pesticides showed more fluctuations in the SPO2 core. Distinct drops were detected at depths of 165 and 125 cm and especially in the depth interval from 67 to 32 cm below the surface. The depth patterns of individual pesticides were very similar, as shown for three selected compounds (Fig. 6). However, some pesticides displayed different behaviour, with maximum values in the upper or lower strata of the sedimentary infill. Azoxystrobin and metazachlor were detected only in the top strata of both cores (CHAL1 and SPO2). The depth profiles of most individual pesticides showed no long-term trends, but those within a single locality were very similar. Distinct drops, which were found in some layers in both abandoned meanders and show very low values or are under the LOQ, are not accompanied by an unusually low TOC or grain-size distribution.

Markedly different pesticide assemblages occurred in the channel bars and abandoned meanders, as well as in each area. Some differences were observed in the Strážnice abandoned meander: higher concentrations of chloridazon-desphenyl (up to 5.6 µg/kg), whereas at other sites, this substance was below the LOQ. The opposite trend was observed for prometryn, which was detected in both abandoned meanders. Chloridazon-desphenyl, in negligible concentrations, was found in the Strážnice abandoned meander only. Pesticide transformation products had higher concentrations in abandoned meanders compared to channel bars. Terbuthylazine-desmethyl-2-hydroxy and terbuthylazine-2-hydroxy, which are both transformation products of terbuthylazine, were frequently detected. Individual pesticides showed different depth profiles between localities; for example, propiconazole, terbutryn, and tebuconazole had the highest values in the upper strata of the Bohumín abandoned meander, whereas their concentrations were highest in the basal strata of the Strážnice abandoned meander. Other pesticides, such as azoxystrobin and metazachlor, revealed similar depth patterns in both abandoned meanders.

Pharmaceuticals were detected in 33 of 34 samples in the Bohumín area and in 27 of 31 samples in the Strážnice area (Fig. 5 and Supplementary Material 2, Table S11). Of the 17 analysed pharmaceutical compounds (see Supplementary Material 1 for the list of all analytes), 11 and caffeine were detected in at least one sample. These compounds belong to different therapeutic classes, including psychoactive, antibiotic, antihypertensive and analgesic drugs. The mean concentrations of 11 pharmaceutical compounds and caffeine exceeded 10 µg/kg in 41% of all samples. In some samples from abandoned meanders, all compounds were under the LOQ, while at least one pharmaceutical compound was detected in each sample from the channel bars. The maximum observed concentration was 100.7 µg/kg for the sum of all pharmaceutical compounds and caffeine in the abandoned meander in the Strážnické Pomoraví area. The most frequently detected compounds in sediments were caffeine, the pharmaceutical carbamazepine (an anticonvulsant and antiepileptic drug), diclofenac (a nonsteroidal anti-inflammatory drug), metoprolol (a beta-blocker used in the treatment of hypertension) and clarithromycin (a macrolide antibiotic), which were detected in 90% of samples. The maximum observed concentrations varied; in the Bohumín area channel bars, the highest concentrations were observed for carbamazepine (5.7 µg/kg), caffeine (5.2 µg/kg) and metoprolol (4.8 µg/kg). The highest maximum concentrations in the Bohumín abandoned meander were recorded for caffeine (22.5 µg/kg), diclofenac (8.8 µg/kg) and carbamazepine (4.8 µg/kg). Lower maximum values were detected in the Strážnice channel bar for clarithromycin (2.9 µg/kg), caffeine and sulphapyridine (a sulfonamide antibiotic). The abandoned meander in this area had the highest levels of caffeine (57.9 µg/kg), diclofenac (37.6 µg/kg) and sulphapyridine (1.9 µg/kg). Metoprolol, with a peak value of 4.8 µg/kg, was detected in the Bohumín area only in both settings, whereas in the Strážnice area, it was below the LOQ. Phenazone was rarely detected, found in only two samples from the Bohumín channel bars. Similarly, N-acetyl sulphapyridine residues were rarely present in seven samples from the channel side bars in the Strážnické Pomoraví area. Trimethoprim was detected in channel side bar samples only. Differences were also found in the depth profiles of individual pharmaceutical compounds. For example, diclofenac displayed an upward-increasing trend in the Strážnice abandoned meander. Tramadol displayed higher concentrations in the upper strata of both abandoned meanders. On the other hand, no long-term trends were observed for other pharmaceuticals. Their depth profiles showed a fluctuating pattern with occasional maxima; for example, this was observed for acebutolol and clarithromycin.

Both groups of emerging pollutants were also normalised to TOC to correct for pollutant concentrations. The depth distribution of pesticides normalised by the TOC revealed a very similar depth pattern (Fig. 6) in terms of local minima and maxima in the Strážnice abandoned meander, while the correspondence was not so high in the Bohumín abandoned meander.

### Deposition environment and consequences for pollutant dispersion

Channel bars consist of fresh sediment that undergoes rapid modification during higher discharges. The deposition of this sediment and the stability are determined primarily by the flow velocity, the sediment texture and the local geometry of the river channel. Similarly, the spatial distribution of particle-bound pollutants is influenced by sediment texture, which is related to water discharges and the progression of flood waves. Coarse-grained bodies with lower pollutant concentrations, as seen in some of our samples, are deposited during peak discharges and tend to be more stable, whereas silt or clay layers are deposited in the aftermath of floods. The low discharge, typical in summer months in both studied rivers, could produce a pesticide concentration effect, resulting in higher levels of pesticides in sediments (Masiá et al., 2013); the same effect can occur for pharmaceuticals. Krein et al. (2013) and Karlsson et al. (2020) noted that pesticide loads could be considerably higher during flood events. On the other hand, floods also dilute micropollutants during peak discharges.

The evolution of abandoned meanders was influenced by the geometry of the former channel. The almost complete infilling observed in the Bohumín abandoned meander was presumably affected by the low diversion angle. On the other hand, higher diversion angles lead to the formation of plug bars, which prevent rapid siltation of new oxbow lakes (Constantine et al., 2010; Dieras et al., 2013; Sedláček et al., 2019); this corresponds to what we observe in the Strážnice abandoned meander. Distinct proximal-to-distal grain-size trends were observed in both studied abandoned meanders, which is in accordance with the results of other studies (e.g., Citterio & Piégay, 2009; Dieras et al., 2013; Sedláček et al., 2019). Therefore, proximal sites closer to the river and plug bars contain predominantly sand, while distal sites are dominated by silt and higher clay content due to decelerated water flow, suggesting a calmer sedimentary environment. The intercalations of coarser layers provide evidence of flood events (Jiang et al., 2024). Sediment, as well as pesticide and pharmaceutical inputs, could be closely linked to floods; we assume that a significant concentration of pollutants could be deposited in the aftermath of floods due to the fallout of fine-grained suspended particles (Bábek et al., 2008), as with channel bars. On the other hand, one peak in the pesticide and pharmaceutical concentrations (Strážnice abandoned meander, see Fig. 4) coincides with sand-rich strata.

Among other factors, the low elevation difference between active river channels and abandoned meanders in natural river reaches contributes to frequent flooding and the rapid evolution of these meanders. During the initial stages of evolution, low-magnitude floods can still reach abandoned meanders within natural river segments, which could explain the sudden peaks in the depth profiles of some contaminants. The ongoing rapid aggradation of the plug bar increases the elevation difference between the active river channel and the abandoned meander, resulting in lower flooding frequency and, consequently, lower contamination input. Therefore, sediment supply is highest in the first years after the initial cut-off (Ahmed, 2024; Citterio & Piégay, 2009; Toonen et al., 2012), and this also holds true for contaminants. The observed decrease in the abundance of the sand fraction in the uppermost layers of the Strážnice abandoned meander supports the assumption of a decline in sediment supply because of the presence of the plug bar (Sedláček et al., 2019, 2020; Toonen et al., 2012). Plug bars and the proximal parts of the abandoned meanders can still receive relatively coarse-grained material, as can be seen in the Bohumín abandoned meander (CHAL2 core).

### Stability and occurrence of micropollutants, factors affecting contaminant distribution

Present-day pesticide concentrations in our samples are residues resulting from degradation and depend on pesticide properties (Fairbairn et al., 2015; Khurshid et al., 2024). Bromilow et al. (2003) and Arias-Estévez et al. (2008) reported rapid degradation of pesticides in the dissolved state and slower degradation of sorbed (immobile) forms, which holds especially true for sediments of abandoned meanders. Present-day pesticide concentrations in the studied rivers could also be influenced by seasonal use and application history, transport processes, river transport length, sediment properties, and organic matter content. Water bodies located near agricultural fields are particularly vulnerable to pesticide contamination (Campo et al., 2013; Kuster et al., 2008). The higher concentrations found in some of our samples may be related to their extensive use, especially during the vegetation period. The minimum concentrations of some pesticides may be transported and deposited during spring floods or after snow melting. Conversely, some pesticides, such as chlorotoluron, are often used as pre- and post-emergence herbicides, with the highest mean loads observed from December to April (Karlsson et al., 2020). Seasonal variations in pesticide spreading explain the weak correlation with the grain size in our samples, especially in flood layers deposited during the pesticide low season. Generally, weak grain-size control was also observed for pharmaceuticals; a positive correlation with clay percentage (*R*^2^ = 0.79) was found in the Strážnice channel bars, and a weak positive correlation between sand percentage and pharmaceuticals was found in the Bohumín area (*R*^2^ = 0.2 and 0.46). Very low concentrations of pollutants were observed in samples with high proportions of coarse sand, due to low sorption onto sand-sized grains and to washing out by flowing water.

In the Strážnice abandoned meander, most of the pesticides had similar depth patterns, suggesting similar behaviours in sediment profiles and minimal migration. On the other hand, polar pesticides, such as chloridazon, chlorotoluron and terbutryn, can readily migrate downward through sediment strata (Fikarová et al., 2018; Konda & Pásztor, 2001), but we did not observe such patterns. The pesticide depth profiles usually showed higher concentrations in the subsurface strata; the second peak was typical for basal layers, possibly due to pesticide accumulation at the transition between the abandoned meander fill and underlying sediments. Unusually low pollutant concentrations in some samples from abandoned meanders may be associated with the coarse-grained size of some layers. Another explanation points to the post-deposition migration of micropollutants from sand-rich strata or their dilution by floodwater. Masiá et al. (2013) reported higher pesticide levels in water than in river sediments, which can contribute to the relatively high pesticide concentrations in channel bars and to the low age of those sedimentary bodies. Photodegradation can also accelerate the removal of emerging pollutants in channel bars.

**Fig. 7:**
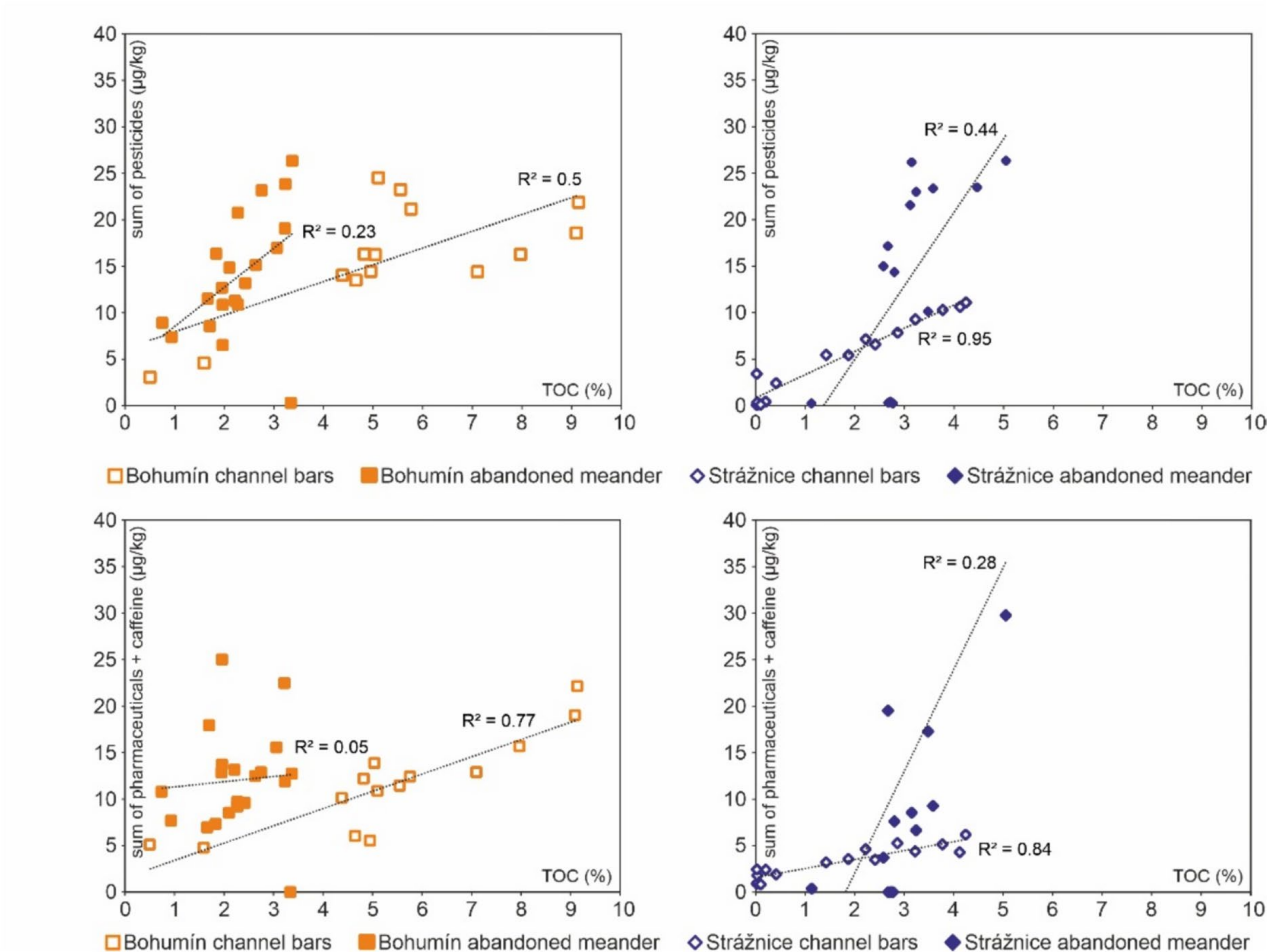
Scatter plots showing correlation of TOC vs. pesticide and pharmaceutical concentrations for all channel bar and abandoned meander samples from both studied areas

In abandoned meanders, the similarities in depth profile patterns of both pesticides and pharmaceuticals suggest that they were transported by the same processes and are not affected significantly by post-depositional migration. Triazine pesticides tend to adsorb to mineral (clay) grains and organic matter (Arias-Estévez et al., 2008; Radović et al., 2015); however, in this sense, no statistically significant correlation was found in our samples (Fig. 7). A weak correlation without statistical significance between the grain size and TOC was reported by Karlsson et al. (2020), suggesting that there are other factors influencing pesticide concentrations. The total concentrations of pharmaceuticals correlate with TOC (Fig. 7), especially in channel bar sediments (*R*^2^ > 0.75). A decrease in grain-size can increase the sorption affinity of organic matter.

Most of the pesticides detected in our samples have a high log *K*_*OW*_, resulting in hydrophobic behaviour and therefore a tendency to accumulate in sediment (Masiá et al., 2013). Conazole fungicides, such as tebuconazole, exhibit strong soil sorption, low to medium solubility, and strong persistence, which can lead to long-term residues (Hvězdová et al., 2018). However, we observed decreasing concentrations with depth, suggesting that this pesticide degrades in older strata. The higher concentrations of pesticide transformation products in the sediments of abandoned meanders may be due to their greater age and, therefore, longer residence time.

Some pharmaceuticals and other compounds, such as caffeine, are biodegradable (Buerge et al., 2003), but the ongoing supply of wastewater may be responsible for their frequent occurrence in river sediments. This could explain the high caffeine concentrations found in some samples. Caffeine concentrations were much higher in abandoned meanders than in channel bars probably because of faster biodegradation in the second setting. A moderately high removal efficiency was reported for diclofenac and metoprolol in pond sediments (Koba et al., 2018). Pharmaceutical removal due to photodegradation and the interaction between water and the atmosphere (Koba et al., 2018) can be limited in the deeper strata of abandoned meander sediments, but biodegradation can occur there. The decay constant of pharmaceuticals is also influenced by sunlight intensity (Hanamoto et al., 2018); therefore, we assume that layers deposited in the summer months can create conditions favourable to faster natural attenuation. Hanamoto et al. (2018) also reported a low rate of natural attenuation of sulphapyridine by biodegradation and volatilisation and identified direct and indirect photolysis as a major loss mechanism during transport. This confirms the role of river length in transport, and this mechanism can further decrease the concentrations of emerging pollutants transported longer distances from the source, such as in the Strážnické Pomoraví area.

### Comparison between pollution in abandoned meanders and channel bars

Distinct differences were found in the occurrence and concentrations of pollutants between channel bars and abandoned meanders. The relative occurrence of most pollutants was higher in channel bars, especially in the Bohumín area, which could be explained by the young age of channel bar sediments and, consequently, the low degree of degradation of deposited emerging pollutants. This also shows the ability of river systems to store pollutants in channel bars. The sorption capacity of sediment particles and the attenuation rate of pharmaceuticals are also influenced by flow velocity (Hanamoto et al., 2018), but the mean annual discharge of the Odra and Morava rivers is comparable. The lowest values of all measured pollutants were found in the Strážnice channel bars. On the other hand, abandoned meander fills showed higher concentrations than channel bars. Some samples from abandoned meanders were characterised by very high concentrations of, for example, caffeine and diclofenac because of decelerated water flow and sediment/pollutant trapping. It should be noted that the distance from the pollution source is also an important factor (Ciazela et al., 2018). Therefore, relatively high contamination values in the Bohumín channel bars can be related to the proximity of the Ostrava industrial agglomeration, while lower concentrations in the Strážnice channel bar sediments could be due to the greater distance from the pollution sources. No significant differences in the levels of pesticide contamination were found between the Bohumín channel bars and the abandoned meander. Therefore, our results showed that channel bars can also store contamination, especially in natural meandering river reaches with numerous channel and point bars. Most rivers have undergone engineering interventions that have dramatically reduced sedimentary body formation in channels. The most important difference between the two studied settings in the ability to store contamination lies in the stability of the deposited strata: channel bars represent short-term contamination, while abandoned meanders exhibit longer-term trends. Therefore, the greatest risk is that incoming sediments, including contaminated sediments, are trapped within abandoned meanders.

### Origin and deposition of studied micropollutants

The high PAH values in the Bohumín area are associated with the proximity of the industrialised Ostrava agglomeration, and the PAHs are mainly of pyrogenic origin. In previous studies from the same region, mixed PAH emission sources (combustion-derived products) were identified (e.g., Geršlová & Schwarzbauer, 2014; Parizek et al., 2024). The unusually high PAH values observed in oxbow lake sediments near Bohumín (Sedláček et al., 2020) were associated with coal matter from coal mining. Coal-rich sediment is a known geosorbent capable of accumulating PAHs through secondary sorption (Pies et al., 2008; Yang et al., 2008).

During the second half of the twentieth century, the widespread distribution of PCBs across former Czechoslovakia resulted in strong pollution by those compounds; the production and use of PCBs were prohibited in 1986 (Kocan et al., 2001; Nondek & Frolikova, 1991). This occurred long before the deposition of the studied sediments. Recently, unknown levels of PCBs have been stored in industrial facilities and old environmental loads, which may be responsible for the sudden increase in PCB levels. For example, the 1997 flood in the Ostrava agglomeration caused severe PCB pollution of the Odra River reach located directly downstream (Ministry of Environment, 2000); this could explain the high PCB concentrations in the Bohumín abandoned meander. Surprisingly, there are relatively high concentrations in the adjacent channel bars, suggesting a source of PCBs in the Odra River.

Pesticide residues are mainly derived from fields due to sheet washing in agricultural areas (Fikarová et al., 2018). The differences between the studied areas reflect pesticide use in local catchments. In general, peak pesticide use occurs during the productive spring and summer months, but pesticide contamination of abandoned meanders could persist into autumn because of ongoing runoff from agricultural fields into nearby water bodies (Lizzote et al., 2009). Pesticide depth profiles can reflect the history of pesticide use, but the specific assemblages of pesticides found in our samples reflect the young ages of both abandoned meanders because they were formed in the last ~25 years for the Bohumín locality and 15 years for the Strážnice locality. Some pesticides previously used in other Czech localities, such as chloridazon and chlorotoluron (Hvězdová et al., 2018), were detected here at negligible concentrations. Similar depth profiles for epoxiconazole and metazachlor were found in both abandoned meanders, suggesting the introduction of both pesticides in the Czech Republic. Diuron and terbutryn have been used to prevent plant growth along roads, railroads, fences, streets, and other urban facilities (Matys Grygar et al., 2020). Propiconazole has higher concentrations likely because it was only recently permitted in the EU (in 2004). This is probably why propiconazole was not found in the deepest strata of the Bohumín abandoned meander, suggesting that these layers were deposited between 1997 and 2004.

Fresh sediments in channel bars represent contemporary bodies; therefore, it is assumed that pollutants in such bodies reflect current inputs and the beginning of their degradation/attenuation. The age of the studied abandoned meanders is higher, so a certain amount of pollutants has undergone degradation during transport before deposition and during deposition. This assumption is also supported by the greater abundance of transformation products in abandoned meanders than in channel bars.

Pesticide levels in abandoned meanders are lower than in Czech soils (Hvězdová et al., 2018), but levels similar to those in Czech soils have been reported in Czech reservoirs (Bábek et al., 2021; Matys Grygar et al., 2020). Some of the pesticides found in our samples, such as terbutryn, are not currently approved for use in the EU. Its concentrations probably reflect residues from historic agricultural applications of this pesticide, or it is still being used illegally. Other formerly used pesticides, such as alachlor, were not detected or were detected only in a few samples (diuron). Triazine pesticides were frequently detected, specifically their transformation products, such as atrazine-2-hydroxy and terbuthylazine-2-hydroxy, due to their strong soil sorption and high persistence. The use of atrazine and simazine was banned in 2004 in the EU, and they were finally retired from the market in 2007; however, both of these substances were still detected in both the channel bars and abandoned meanders. However, simazine and atrazine, the parent compounds of 2-hydroxy-terbuthylazine, will still be emitted into the environment as impurities because of the application of terbuthylazine (Hvězdová et al., 2018; Karlsson et al., 2020). Simazine, atrazine, and some other pesticide residues are persistent under anoxic conditions (Karlsson et al., 2020), which can develop in abandoned meanders. Their occurrence suggests a slow but continuous release from agricultural areas in the catchment due to sheet washing. The concentration of the transformation product atrazine-2-hydroxy was higher than that of its parental compound, atrazine. This is probably the legacy of its long-term intensive use before it was abandoned (Hvězdová et al., 2018). The same finding holds true for simazine-2-hydroxy, but simazine itself, as the parent compound, could not be detected in the sediment samples. Atrazine, which is relatively persistent in soils, is known for its slow movement through the sediment profile by diffusion (Fikarová et al., 2018; Konda & Pásztor, 2001; Morvan et al., 2006). This was observed in the depth profile of its transformation product in the Strážnice abandoned meander (atrazine itself was below the LOQ), whereas in the Bohumín abandoned meander, the concentrations of both atrazine and atrazine-2-hydroxy were more homogeneously distributed with depth. Triazole pesticides, including epoxiconazole and tebuconazole, were frequently applied; they are considered more persistent, but epoxiconazole was detected only in the upper parts of cores from abandoned meanders. Terbuthylazine metabolites, as persistent and mobile chemicals, are frequently detected in environmental systems in Europe (e.g., Karlsson et al., 2020). The most frequently found pesticides, as well as those with the highest values in our samples, belong to herbicides and fungicides applied to cereals, corn, rapeseed or potatoes. All of these are crops with a high proportion in the studied areas. Previously, the highest levels of pesticides were observed in fields where rapeseed and wheat were grown (Zumr, 2022), which also applies to both studied areas.

Compared to pesticides, most pharmaceuticals were detected at very similar concentrations, except for channel bars in the Strážnice area, where pesticides had concentrations 2 times higher, but the total values for both groups were low. Radović et al. (2015) reported detecting pharmaceuticals less frequently than pesticides. Carbamazepine has frequently been detected in river sediments worldwide; for example, it has been detected in Spain (Ferreira da Silva et al., 2011; Martín et al., 2012), the UK (Zhou & Broodbank, 2014) and Hungary (Kondor et al., 2022). Its relatively frequent presence in our samples is due to its extensive use, persistence in the aquatic environment, resistance to biodegradation and low removal rate in wastewater treatment (Clara et al., 2004; Ternes et al., 2002). Sulphapyridine is no longer prescribed for the treatment of infections in humans (Kerrigan et al., 2018). However, it is used in veterinary medicine. In small streams, pharmaceutical concentrations can be higher, sometimes exceeding 100 µg/kg, because of lower dilution, reflecting the almost direct release of pharmaceuticals from wastewater treatment, as reported by Kondor et al. (2022). However, we measured relatively high concentrations of pharmaceuticals and caffeine in some of our samples, reflecting their regional use. Given the dilution of pharmaceuticals by the Morava and Odra rivers, the input concentrations were likely very high. The distance from the pollution source appears to be the most important factor influencing the occurrence and concentrations of pharmaceuticals in channel bar sediments, as evidenced by higher concentrations in the Bohumín area than in the Strážnice area. The densely populated Ostrava agglomeration, which is likely a significant source of pharmaceuticals, is located just a few kilometres upstream. On the other hand, the much lower concentrations found for similar grain-size fractions in the Strážnice area are the consequence of this area’s greater distance from larger sources and are also due to the dilution effect in the coarse-grained matrix. The longer the transport of pharmaceuticals, the more they degrade and attenuate (Hanamoto et al., 2018). However, their elimination is highly substance-specific (Kunkel & Radke, 2012), suggesting that the pharmaceutical concentrations found in our samples may result from their extensive use and different elimination rates.

## Conclusions

Abandoned meanders, especially in natural river reaches, are suitable for the monitoring and assessment of contamination by modern pollutants because of their frequent flooding and high spatial and temporal resolutions. We also confirmed that channel bar sediments can be used to monitor the current level of organic pollutant contamination in river systems. Some pesticides and pharmaceuticals are stable in the sedimentary record of these meanders; therefore, they can persist in sediments for many years after they are abandoned. A higher environmental risk is present in abandoned meanders because of high SARs pollutant trapping and the high persistence of some of the studied compounds in sediments. Channel bar sediments that are already polluted could become additional sources of pollution. Pollution levels in both studied settings reflect input from the upstream catchment and result from the dynamic interplay of several factors, including the supply, hydrological regime, conditions during transport and deposition, sediment properties and degradation. The distance from the source is important because relatively high concentrations of pharmaceuticals in channel bars from the Bohumín area may be due to their proximity to the upstream Ostrava agglomeration. Channel bar sediments are suitable for studying the regional impact of freshly applied pesticides on runoff. The environmental risk is generally low because of the dynamic nature of channel sediments and dilution by flowing water. Pesticide assemblages are also influenced by transport duration, the timing of pesticide application and the properties of the pesticides themselves. Regarding pharmaceuticals, their occurrence and levels depend on human use and the wastewater treatment’s ability to degrade them. The TOC content is the primary factor influencing micropollutant concentrations in the studied samples. Pharmaceutical concentrations are not related to floods because most of their residues enter rivers directly through wastewater treatment plants. On the other hand, abandoned meanders are flooded only during flood events. However, recommendations for future research include regular monitoring over a longer time horizon at selected sites to identify temporal trends in contamination.

## Supplementary Information

Below is the link to the electronic supplementary material.

ESM 1 Supplementary Material 1 (DOCX 238 KB)

## Data Availability

No datasets were generated or analysed during the current study.
