# Supplementary material for: Comparison of contamination by polycyclic aromatic hydrocarbons, pesticides and pharmaceuticals in abandoned meanders and channel bars, Czech Republic
Source: Environ Monit Assess. 2025 Dec 24;198(1):66. doi: 10.1007/s10661-025-14928-0 (PMC12738593; doi:10.1007/s10661-025-14928-0)
Supplement: Supplementary file 1 — Supplementary Material 1 (DOCX 238 KB) [file 10661_2025_14928_MOESM1_ESM.docx]

**Supplementary material to** **paper “Comparison of contamination by polycyclic aromatic hydrocarbons, pesticides, and pharmaceuticals in abandoned meanders and channel bars, Czech Republic” by J. Sedláček, J. Tolaszová, Z. Lenďáková, O. Koukal and L. Maloušek**

**Supplementary Material 1: Detailed description of methodology for organic pollutants analysis**

Extractions were performed in 50 mL conical polypropylene centrifuge tubes (Fisher Scientific, Czech Republic) with ceramic grinding stones in MiniG™ 1600 from SPEX®SamplePrep (USA) or in 50 mL flat-bottom flasks (ThermoFisher, Czech Republic). Vortex Mix (Chromservis, Czech Republic) was also used for extraction. For pesticides and pharmaceuticals, methanol (Honeywell, USA) and water (Merck Millipore, Germany) were used as solvents. PAHs and PCBs were extracted by QuEChERS with ethyl acetate (Chromservis, Czech Republic), water (Merck Millipore, Germany), MgSO_4_ (Merck Millipore, Germany) and NaCl (Penta, Czech Republic). Vortex Mix (Chromservis, Czech Republic) was also used for extraction. Centrifuge (Remi X5 R-10 M, Chromservis, Czech Republic and Biosan Microspin 12, Merci, Czech Republic) was used to centrifuge the sample extracts. Nylon syringe filters (0.22 μm, Chromservis, Czech Republic) were used to filter the extracts. The Rxi PAH 40 m × 0.18 mm, 0.07 μm column (Restek, USA) and the DB-EUPAH column 20 m × 0.18 mm, 0.14 μm for the determination of PAHs (Agilent Technologies, USA) and the HP5-MS 30 m × 0.25 mm, 0.25 μm column for PCB (Agilent Technologies, USA) were used. Sediment extracts were measured on a gas chromatograph (7890B, Agilent Technologies, USA) with a mass spectrometer (7000D triple quadrupole, Agilent Technologies, USA). The data was evaluated using MassHunter software version B.09.00 from Agilent Technologies. Microsoft Excel was used for further data processing.

**Pesticides and pharmaceuticals, Extraction and LC-MS/MS analysis**

5 g of a soil subsample was weighted into 50 mL polypropylene vial, 10 mL of water and 10 mL of methanol were added and vortexed for 5 s. These lyophilizated samples were extracted by shaking - the samples were placed for 3 minutes, 1500 rpm intensity of shaking in MiniG vertical shaker with two grinding stones. These shaked samples were put in a centrifuge for 5 min at 4500 rpm, then the filtration via 0.22 µm nylon syringe filter was performed. 0.2 ml of the sample extract was transferred to the glass vial and into the 0.2 ml sample extract, the 0,8 ml deionized water was added because of matrix effect. The samples were diluted five times. The extracts were analyzed on LC-MS/MS.

Gradient elution was used for chromatographic analysis, the time course is described in Table S1 below. Water (A) with 0.5 mM NH_4_F and 0.25 mM CH_3_COONH_4_ and methanol (B) with 0.25 mM NH_4_F were used as the mobile phase. The determination was performed on a Kinetex column (2.6 μm C18 100 Å 150 x 2.1 mm). The temperature on the column was 40°C. The sample injection on the column was 20 μl. Final data were obtained from measurements in the selective multiple reaction record (dMRM) with two or more analyte transitions. All transitions were obtained by tuning the individual analytes. MS conditions (Table S2) were optimized to obtain precursor ions with maximum intensity.

**Table S1: The time course.**

| **Time (min)** | **A (%)** | **B (%)** | **Flow (ml/min)** |
| --- | --- | --- | --- |
| 0 | 80 | 20 | 0.3 |
| 9 | 0 | 100 | 0.4 |
| 11 | 0 | 100 | 0.45 |
| 11.1 | 80 | 20 | 0.5 |
| 16 | 80 | 20 | 0.5 |

**Table S2: MS conditions for the determination of pesticides and pharmaceuticals.**

| **Source parameters** | **Value (ESI+)** | **Value (ESI-)** |  | **Parameters Ion Funnel** | **Value** |
| --- | --- | --- | --- | --- | --- |
| **Gas Temp (°C)** | 150 | 150 |  | **Positive High Pressure RF** | 110 |
| **Gas Flow (l/min)** | 14 | 14 |  | **Positive Low Pressure RF** | 70 |
| **Nebulizer (psi)** | 35 | 35 |  | **Negative High Pressure RF** | 90 |
| **Sheath Gas Heater** | 380 | 380 |  | **Negative Low Pressure RF** | 60 |
| **Sheath Gas Flow** | 11 | 11 |  |  |  |
| **Capillary (V)** | 3000 | 3000 |  |  |  |
| **V charging** | 0 | 900 |  |  |  |

PAHs and PCBs

5 g of a soil subsample was weighted into 50 mL polypropylene vial, spiked by 1 ml of 20 µg/mL of deuterated standards in acetone and vortexed for 5s. After 15 min, 10 mL of water and 10 mL of ethylacetate were added and vortexed for 5 s. These lyophilizated samples were extracted by QuEChERS method using 4 g MgSO_4_ and 1 g NaCl salts, shaked manually and vortexed for another 10 s. The samples were placed for 5 minutes, 1500 rpm intensity of shaking in MiniG vertical shaker with two grinding stones. These shaked samples were put in a centrifuge for 5 min at 4500 rpm, then the clean-up step (d-SPE) was performed using 150 mg of MgSO_4_ and 50 mg of C18, vortexed for 5 s. d-SPE vials were centrifuged for 2 min, 12000 rpm. The extracts were transferred to a glass vial for the analysis on GC-MS/MS.

The analyses of extracts were performed by GC with a triple quadrupole mass spectrometer, electron ionization at 70 eV. The chromatographic separation of PAH was performed using a chromatographic column DB-EUPAH (length 20 m, ID 0.18 mm, film thickness 0.14 μm). The sample injection was 1 µl in splitless mode with a double taper liner (5190-4007 Splitless double taper UI). The flow rate on the column was 1.5 ml/min. For the chromatographic analysis, the temperature gradient was used.

The oven temperatures were programmed from 80°C (initial hold time 1 min) to 200°C at 45°C/min (hold time 0 min), from 200°C to 300°C at 5°C/min (hold time 0 min), then from 300°C to 320°C at 10°C/min (hold time 10 min). Each analysis took 30 min. Helium (5.5) was used as a carrier gas at a constant flow of 1,5 mL/min, nitrogen was used as a collision gas.

The HP-5MS column (30 m x 250 μm x 0.25 μm) was used for the determination of PCBs. The oven temperatures were programmed from 60°C (initial hold time 1 min) to 170°C at 40°C/min (hold time 0 min), from 170°C to 310°C at 10°C/min (hold time 4.5 min). Each analysis took 22 min. Helium (5.5) was used as a carrier gas at a constant flow of 1 mL/min, nitrogen was used as a collision gas. The injected volume was 1 μL in splitless mode with a splitless liner double taper for PAH as well as for PCB.

**Method characteristics**

The following characteristics were studied: selectivity, linearity, detection and quantification limits (LOD and LOQ), repeatability, recovery, accuracy based on reference material (if available), and matrix effect. Selectivity was ensured using MRM transitions (Table S3). The limit of detection (LOD) and the limit of quantification (LOQ) were determined as three times or ten times the standard deviation of the signal of the standard solution at the lowest standard concentration in the sediment. The LOD and LOQ of all analytes are listed in Tables S4 and S6. These limits were verified using repeated measurements of standards (n=7) or extracted spiked samples (n=7) at concentrations close to the LOQ as well as higher concentration levels. For testing linearity, linear regression with 9 concentration levels was used. Repeatability was examined using the same instrument, the same operator, and six repetitions; overall repeatability was calculated from the average of the entire procedure, from sediment weighing, through extraction, to the prepared extract solution for analysis of enriched sediments, expressed as a relative standard deviation. Recoveries were calculated as the percentage of the analysed signal in the sample solution compared to the standard solution of the same concentration. The overall uncertainty (U) was calculated as the standard combined uncertainty, which was then expanded by a factor of 2, corresponding to a 95% confidence level (k=2). The standard combined uncertainty was expressed as the square root of the sum of the squares of the standard uncertainties determined from repeatability at the concentration level of the first calibration point and at a higher concentration level in sediments. The matrix effect was also studied, which is often monitored in the field of analytical chemistry and can cause enhancement or suppression of analytical signals. The matrix effect refers to the effect of all other components of the sample besides the analyte being determined, meaning any influence that the sample matrix can have on the analytical result. To quantify the matrix effect, a standard of known concentration was prepared along with an extract, which was spiked with the standard at the same concentration. Then the standard, along with the spiked sample and a blank sample was analysed.

**PAHs and PCBs**

The characteristics of the method are summarized in Table S5. The calibration curve was constructed using nine concentration levels (0.1, 0.5, 1, 5, 10, 20, 50, 100, 200 ng/ml) with an internal standard (at a final concentration of 50 ng/ml) for PAHs (deuterated PAHs), and PCBs (Pentachloronitrobenzene-13C6). The internal standard method was used for PAHs, and PCBs in sediments. Satisfactory correlation coefficients (0.997-0.999) were obtained for all compounds, demonstrating that the method is linear across the entire tested calibration range. Recovery was expressed as the mean of seven samples spiked with all determined analytes at concentration levels of LOQ and 100 ng/ml for sediments after subtracting the blank sample. The uncertainty of determination was 15% for PAHs, 20% for PCBs. The accuracy of the proposed method was determined using values obtained from a certified reference material. The obtained data showed acceptable results for PAHs. Table S5 shows the values of the certified material and the values obtained after extraction using the optimized QuEChERS extraction procedure.

**Table S3: MRM transitions, Retention time and Collision energy of PAHs and PCBs, pharmaceuticals and pesticides.**

| **PAH** | **Precursor ion (MS1)** | **Product ion (MS2)** | **Retention time (min)** | **Collision energy (V)** | **PAH** | **Precursor ion (MS1)** | **Product ion (MS2)** | **Retention time (min)** | **Collision energy (V)** |
| --- | --- | --- | --- | --- | --- | --- | --- | --- | --- |
| **Acenaphthene** | 154 | 153 | 4.64 | 20 | **Dibenz[a.h]anthracene** | 278 | 278 | 25.69 | 5 |
|  | 154 | 152 | 4.64 | 36 |  | 278 | 276 | 25.69 | 52 |
|  | 153.1 | 127 | 4.64 | 30 |  | 278 | 274 | 25.69 | 52 |
| **Acenaphthylene** | 152.1 | 126 | 4.54 | 30 | **Phenanthrene** | 178 | 152 | 6.65 | 26 |
|  | 152 | 151 | 4.54 | 22 |  | 178 | 151 | 6.65 | 40 |
|  | 152 | 150 | 4.54 | 32 |  | 152.1 | 151.1 | 6.65 | 15 |
| **Anthracene** | 178 | 152 | 6.71 | 26 | **Fluoranthene** | 202 | 202 | 9.69 | 5 |
|  | 178 | 151 | 6.71 | 40 |  | 202 | 200 | 9.69 | 40 |
|  | 176.1 | 150.1 | 6.71 | 25 |  | 202 | 150 | 9.69 | 52 |
| **Benzo[a]anthracene** | 228 | 228 | 15.25 | 5 | **Fluorene** | 166.1 | 165.1 | 5.11 | 15 |
|  | 228 | 226 | 15.25 | 38 |  | 166 | 166 | 5.11 | 20 |
|  | 228 | 202 | 15.25 | 30 |  | 165.1 | 164.1 | 5.11 | 20 |
| **Benzo[a]pyrene** | 252 | 252 | 21.68 | 5 | **Chrysene** | 228 | 228 | 15.61 | 5 |
|  | 252 | 250 | 21.68 | 44 |  | 228 | 226 | 15.61 | 38 |
|  | 252 | 224 | 21.68 | 31 |  | 113.1 | 112.1 | 15.61 | 10 |
| **Benzo[b]fluoranthene** | 252.1 | 250.1 | 19.98 | 35 | **Indeno[1.2.3-cd]pyrene** | 276 | 276 | 25.67 | 10 |
|  | 252 | 252 | 19.98 | 5 |  | 276 | 274 | 25.67 | 50 |
|  | 252 | 224 | 19.98 | 31 |  | 276 | 248 | 25.67 | 52 |
|  | 252.1 | 250.1 | 20.09 | 30 | **Naphthalene** | 128 | 128 | 3.43 | 15 |
|  | 252 | 252 | 20.09 | 5 |  | 128 | 127.1 | 3.43 | 15 |
|  | 252 | 224 | 20.09 | 31 |  | 128 | 102.1 | 3.43 | 15 |
| **Benzo[g.h.i]perylene** | 276 | 276 | 26.65 | 10 | **Pyrene** | 202 | 202 | 10.81 | 5 |
|  | 276 | 274 | 26.65 | 50 |  | 202 | 200 | 10.81 | 42 |
|  | 276 | 248 | 26.65 | 52 |  | 202 | 150 | 10.81 | 52 |
| **Benzo[k]fluoranthene** | 252.1 | 250.1 | 20.09 | 30 |  |  |  |  |  |
|  | 252 | 252 | 20.09 | 5 |  |  |  |  |  |
|  | 252 | 224 | 20.09 | 31 |  |  |  |  |  |

| **PCB** | **Precursor ion (MS1)** | **Product ion (MS2)** | **Retention time (min)** | **Collision energy (V)** | **PCB** | **Precursor ion (MS1)** | **Product ion (MS2)** | **Retention time (min)** | **Collision energy (V)** |
| --- | --- | --- | --- | --- | --- | --- | --- | --- | --- |
| **2,4,4'-Trichlorobiphenyl (PCB 28)** | 258 | 186 | 12.08 | 25 | **2,2',4,4',5,5'- Hexachlorobiphenyl (PCB 153)** | 361.9 | 289.9 | 15.93 | 25 |
|  | 256 | 186 | 12.08 | 25 |  | 359.9 | 289.9 | 15.93 | 25 |
|  | 186 | 151 | 12.08 | 25 |  | 287.9 | 217.9 | 15.93 | 40 |
| **2,2',5,5'-Tetrachlorobiphenyl (PCB 52)** | 291.9 | 221.9 | 12.69 | 25 | **2,2',3,4,4',5'- Hexachlorobphenyl  (PCB 138)** | 361.9 | 289.9 | 16.48 | 30 |
|  | 289.9 | 219.9 | 12.69 | 25 |  | 359.9 | 289.9 | 16.48 | 30 |
|  | 255 | 220 | 12.69 | 10 |  | 287.9 | 217.9 | 16.48 | 40 |
| **2,2',4,5,5'-Pentachlorobiphenyl (PCB 101)** | 325.9 | 255.9 | 14.33 | 30 | **2,2',3,4,4',5,5'- Heptachlorobiphenyl (PCB 180)** | 395.8 | 325.8 | 17.72 | 30 |
|  | 325.9 | 253.9 | 14.33 | 30 |  | 393.8 | 358.8 | 17.72 | 15 |
|  | 253.9 | 184 | 14.33 | 35 |  | 393.8 | 323.8 | 17.72 | 30 |

| **Pharmaceuticals** | **Precurzor ion (MS1)** | **Product ion (MS2)** | **Retention time (min)** | **Collision energy (V)** | **Polarity** | **Pharmaceuticals** | **Precurzor ion (MS1)** | **Product ion (MS2)** | **Retention time (min)** | **Collision energy (V)** | **Polarity** |
| --- | --- | --- | --- | --- | --- | --- | --- | --- | --- | --- | --- |
| **3-hydroxycarbamazepine** | 253 | 167.1 | 5.54 | 40 | Positive | **N-acetylsulfamethoxazole** | 296 | 65.3 | 4.49 | 48 | Positive |
|  | 253 | 179.9 | 5.54 | 56 | Positive |  | 296 | 108.1 | 4.49 | 36 | Positive |
|  | 253 | 208 | 5.54 | 24 | Positive | **N-acetylsulfapyridine** | 292 | 65.2 | 3.17 | 44 | Positive |
|  | 253 | 210.1 | 5.54 | 20 | Positive |  | 292 | 94.1 | 3.17 | 36 | Positive |
| **Acebutolol** | 337.2 | 72.3 | 5.59 | 32 | Positive |  | 292 | 108.2 | 3.17 | 28 | Positive |
|  | 337.2 | 74.2 | 5.59 | 32 | Positive |  | 292 | 134 | 3.17 | 24 | Positive |
|  | 337.2 | 116.1 | 5.59 | 24 | Positive | **Phenazone** | 189.1 | 130.1 | 3.83 | 40 | Positive |
|  | 337.2 | 319.3 | 5.59 | 16 | Positive |  | 189.1 | 131.1 | 3.83 | 20 | Positive |
| **Caffeine** | 195.1 | 56 | 3.16 | 36 | Positive |  | 189.1 | 147.1 | 3.83 | 20 | Positive |
|  | 195.1 | 83 | 3.16 | 32 | Positive | **Propyphenazone** | 231.1 | 118.1 | 6.25 | 40 | Positive |
|  | 195.1 | 110 | 3.16 | 24 | Positive |  | 231.1 | 130.1 | 6.25 | 40 | Positive |
|  | 195.1 | 138 | 3.16 | 20 | Positive |  | 231.1 | 189.1 | 6.25 | 20 | Positive |
| **Carbamazepine** | 237.1 | 165.1 | 6.21 | 56 | Positive | **Sulfadiazin** | 250.9 | 65 | 1.89 | 48 | Positive |
|  | 237.1 | 179.1 | 6.21 | 41 | Positive |  | 250.9 | 91.8 | 1.89 | 28 | Positive |
|  | 237.1 | 193.1 | 6.21 | 34 | Positive |  | 250.9 | 107.8 | 1.89 | 24 | Positive |
|  | 237.1 | 194.1 | 6.21 | 24 | Positive |  | 250.9 | 155.8 | 1.89 | 12 | Positive |
| **Clarithromycin** | 748.5 | 83 | 8.85 | 40 | Positive | **Sulfamerazine** | 265.1 | 92 | 2.6 | 40 | Positive |
|  | 748.5 | 116.1 | 8.85 | 40 | Positive |  | 265.1 | 108 | 2.6 | 20 | Positive |
|  | 748.5 | 158.1 | 8.85 | 40 | Positive |  | 265.1 | 110.1 | 2.6 | 20 | Positive |
|  | 748.5 | 558.4 | 8.85 | 20 | Positive |  | 265.1 | 156 | 2.6 | 10 | Positive |
|  | 748.5 | 590.4 | 8.85 | 20 | Positive |  | 265.1 | 172 | 2.6 | 10 | Positive |
| **Diclofenac** | 296 | 179.1 | 6.07 | 64 | Positive |  | 265.1 | 199.1 | 2.6 | 20 | Positive |
|  | 296 | 214.1 | 6.07 | 48 | Positive | **Sulfamethoxazole** | 254.1 | 92 | 2.86 | 20 | Positive |
|  | 296 | 215.1 | 6.07 | 16 | Positive |  | 254.1 | 108 | 2.86 | 20 | Positive |
|  | 296 | 250 | 6.07 | 8 | Positive |  | 254.1 | 156 | 2.86 | 10 | Positive |
| **Ketoprofen** | 254.9 | 76.8 | 4.98 | 52 | Positive | **Sulfapyridine** | 250.1 | 91.9 | 2.41 | 28 | Positive |
|  | 254.9 | 208.9 | 4.98 | 12 | Positive |  | 250.1 | 155.9 | 2.41 | 16 | Positive |
| **Metoprolol** | 268.2 | 98.1 | 5.71 | 20 | Positive | **Tramadol** | 264.2 | 42.2 | 5.74 | 48 | Positive |
|  | 268.2 | 103.1 | 5.71 | 40 | Positive |  | 264.2 | 58.2 | 5.74 | 20 | Positive |
|  | 268.2 | 105.1 | 5.71 | 40 | Positive |  | 264.2 | 246 | 5.74 | 12 | Positive |
|  | 268.2 | 116.1 | 5.71 | 20 | Positive |  |  |  |  |  |  |
|  | 268.2 | 121.1 | 5.71 | 40 | Positive |  |  |  |  |  |  |

| **Pesticides** | **Precurzor ion (MS1)** | **Product ion (MS2)** | **Retention time (min)** | **Collision energy (V)** | **Polarity** | **Pesticides** | **Precurzor ion (MS1)** | **Product ion (MS2)** | **Retention time (min)** | **Collision energy (V)** | **Polarity** |
| --- | --- | --- | --- | --- | --- | --- | --- | --- | --- | --- | --- |
| **2,4,5-T** | 252.9 | 194.9 | 5.27 | 12 | negative | **Isoproturon-monodesmethyl** | 193.13 | 94.1 | 6.67 | 24 | positive |
|  | 254.9 | 196.9 | 5.27 | 12 | negative |  | 193.13 | 151.3 | 6.67 | 16 | positive |
| **2,4-D** | 218.9 | 160.9 | 4.23 | 12 | negative | **Linuron** | 249 | 133 | 7.26 | 36 | positive |
|  | 220.9 | 162.9 | 4.23 | 12 | negative |  | 249 | 160 | 7.26 | 20 | positive |
| **2,6-dichlorobenzamide** | 189.98 | 109 | 3.19 | 45 | positive |  | 249 | 182.1 | 7.26 | 16 | positive |
|  | 189.98 | 145 | 3.19 | 30 | positive | **MCPA** | 199 | 141 | 4.3 | 16 | negative |
|  | 189.98 | 173 | 3.19 | 16 | positive |  | 201 | 143 | 4.3 | 16 | negative |
| **Acetochlor** | 270.2 | 148 | 7.91 | 16 | positive | **MCPB** | 227.05 | 141.01 | 6.3 | 8 | negative |
|  | 270.2 | 224.1 | 7.91 | 8 | positive |  | 229.05 | 143.01 | 6.3 | 8 | negative |
| **Acetochlor ESA** | 314 | 80 | 5.01 | 60 | negative | **Mecoprop** | 213 | 71 | 4.96 | 8 | negative |
|  | 314 | 121 | 5.01 | 24 | negative |  | 213 | 141 | 4.96 | 16 | negative |
|  | 314 | 144.1 | 5.01 | 36 | negative |  | 215 | 143 | 4.96 | 16 | negative |
|  | 270.1 | 120.1 | 5.01 | 28 | positive | **Metamitron** | 203.116 | 103.9 | 4.17 | 24 | positive |
|  | 270.1 | 148.1 | 5.01 | 16 | positive |  | 203.116 | 175.1 | 4.17 | 20 | positive |
| **Acetochlor OA** | 264 | 144 | 4.67 | 32 | negative | **Metazachlor** | 278.2 | 134 | 6.66 | 20 | positive |
|  | 264 | 146 | 4.67 | 8 | negative |  | 278.2 | 210.1 | 6.66 | 8 | positive |
| **Alachlor** | 270.2 | 162 | 7.94 | 24 | positive | **Metazachlor ESA** | 322.08 | 80 | 3.41 | 60 | negative |
|  | 270.2 | 238.1 | 7.94 | 8 | positive |  | 322.08 | 121 | 3.41 | 28 | negative |
| **Alachlor ESA** | 314 | 80.3 | 5.01 | 36 | negative |  | 322.08 | 148 | 3.41 | 24 | negative |
|  | 314 | 121.2 | 5.01 | 24 | negative | **Metazachlor OA** | 272.1 | 67.2 | 3.1 | 32 | negative |
|  | 314 | 160.1 | 5.01 | 18 | negative | **Methabenzthiazuron** | 222.1 | 123 | 6.67 | 52 | positive |
|  | 284.1 | 134.1 | 5.01 | 26 | positive |  | 222.1 | 149.9 | 6.67 | 44 | positive |
|  | 284.1 | 162.1 | 5.01 | 16 | positive | **Metobromuron** | 259 | 148 | 6.53 | 14 | positive |
| **Alachlor OA** | 264.1 | 158 | 4.64 | 28 | negative |  | 259 | 170 | 6.53 | 20 | positive |
|  | 264.1 | 160 | 4.64 | 8 | negative | **Metolachlor** | 284.1 | 176.2 | 7.98 | 28 | positive |
| **Ametryn** | 228.1 | 91.1 | 7.32 | 28 | positive |  | 284.1 | 252.2 | 7.98 | 12 | positive |
|  | 228.1 | 95.8 | 7.32 | 28 | positive | **Metolachlor ESA** | 328.1 | 80.1 | 5.2 | 56 | negative |
|  | 228.1 | 186.1 | 7.32 | 20 | positive |  | 328.1 | 120.8 | 5.2 | 24 | negative |
| **Atrazine** | 216.1 | 68.1 | 6.67 | 40 | positive |  | 328.1 | 135.1 | 5.2 | 28 | negative |
|  | 216.1 | 96 | 6.67 | 28 | positive | **Metolachlor OA** | 278.1 | 158 | 5.14 | 20 | negative |
|  | 216.1 | 174.1 | 6.67 | 16 | positive |  | 278.1 | 174.1 | 5.14 | 16 | negative |
| **Atrazine-2-hydroxy** | 198.1 | 86 | 5.01 | 24 | positive |  | 278.1 | 206 | 5.14 | 8 | negative |
|  | 198.1 | 156.1 | 5.01 | 16 | positive | **Metoxuron** | 229 | 46 | 5.22 | 16 | positive |
| **Atrazine-desethyl** | 188.1 | 104 | 4.66 | 28 | positive |  | 229 | 72 | 5.22 | 26 | positive |
|  | 188.1 | 146 | 4.66 | 16 | positive |  | 229 | 156 | 5.22 | 28 | positive |
| **Atrazine-desethyl-desisopropyl** | 146.02 | 42.9 | 1.68 | 32 | positive | **Metribuzin** | 215 | 84.1 | 5.73 | 20 | positive |
|  | 146.02 | 78.8 | 1.68 | 20 | positive |  | 215 | 187.1 | 5.73 | 20 | positive |
|  | 146.02 | 104 | 1.68 | 20 | positive | **Metribuzin-desamino** | 199.9 | 116 | 5.94 | 24 | positive |
| **Atrazine-desisopropyl** | 174.1 | 96.1 | 3.63 | 20 | positive |  | 199.9 | 172 | 5.94 | 20 | positive |
|  | 174.1 | 132 | 3.63 | 20 | positive | **Monolinuron** | 215 | 126 | 6.26 | 16 | positive |
| **Azoxystrobin** | 404.1 | 344.2 | 7.39 | 24 | positive |  | 215 | 147.9 | 6.26 | 12 | positive |
|  | 404.1 | 372.1 | 7.39 | 13 | positive | **Napropamide** | 271.9 | 129 | 7.98 | 16 | positive |
| **Bentazone** | 239.1 | 132 | 2.48 | 30 | negative |  | 271.9 | 171 | 7.98 | 20 | positive |
|  | 239.1 | 175.1 | 2.48 | 24 | negative | **Parathion-methyl** | 248 | 108 | 7.24 | 42 | negative |
| **Clomazone** | 239.9 | 88.9 | 7.1 | 60 | positive |  | 248 | 138 | 7.24 | 20 | negative |
|  | 239.9 | 124.7 | 7.1 | 24 | positive |  | 248 | 154 | 7.24 | 8 | negative |
| **Cyanazine** | 241 | 103.7 | 5.63 | 40 | positive | **Pendimethalin** | 282.089 | 194 | 9.278 | 20 | positive |
|  | 241 | 213.9 | 5.63 | 16 | positive |  | 282.089 | 212 | 9.278 | 8 | positive |
| **Cyproconazole** | 292.1 | 70.1 | 7.76 | 22 | positive | **Phosalone** | 368 | 111.1 | 8.5 | 40 | positive |
|  | 292.1 | 125.1 | 7.76 | 35 | positive |  | 368 | 182 | 8.5 | 18 | positive |
| **Desmetryn** | 214.14 | 82.1 | 6.7 | 36 | positive | **Pirimiphos-methyl** | 306 | 108.1 | 8.57 | 32 | positive |
|  | 214.14 | 172.1 | 6.7 | 16 | positive |  | 306 | 164.2 | 8.57 | 24 | positive |
| **Diazinon** | 305.1 | 96.9 | 8.41 | 44 | positive | **Prochloraz** | 376 | 308 | 8.59 | 8 | positive |
|  | 305.1 | 169.1 | 8.41 | 22 | positive |  | 378 | 310 | 8.59 | 8 | positive |
| **Difenoconazole** | 406 | 251 | 8.72 | 24 | positive | **Prometryn** | 242.1 | 158 | 7.89 | 28 | positive |
|  | 406 | 337 | 8.72 | 16 | positive |  | 242.1 | 200.1 | 7.89 | 20 | positive |
| **Diflufenican** | 394.8 | 246 | 8.82 | 44 | positive | **Propachlor** | 212.1 | 94 | 6.78 | 32 | positive |
|  | 394.8 | 265.9 | 8.82 | 24 | positive |  | 212.1 | 170 | 6.78 | 16 | positive |
| **Dichlorprop** | 233 | 160.96 | 4.99 | 10 | negative | **Propachlor ESA** | 256.1 | 80 | 3.365 | 40 | negative |
|  | 235 | 162.95 | 4.99 | 10 | negative |  | 256.1 | 121 | 3.365 | 20 | negative |
| **Dimethachlor** | 256.1 | 148.1 | 6.94 | 28 | positive | **Propazine** | 230.2 | 146 | 7.32 | 28 | positive |
|  | 256.1 | 224.1 | 6.94 | 12 | positive |  | 230.2 | 188.1 | 7.32 | 16 | positive |
| **Dimethachlor ESA** | 302 | 174 | 3.8 | 28 | positive | **Propiconazole** | 342 | 69.1 | 8.38 | 20 | positive |
|  | 302 | 270 | 3.8 | 12 | positive |  | 342 | 158.9 | 8.38 | 28 | positive |
| **Dimethachlor OA** | 252 | 132 | 3.59 | 24 | positive | **Quinmerac** | 222.05 | 141 | 1.883 | 40 | positive |
|  | 252 | 220.1 | 3.59 | 12 | positive |  | 222.05 | 175.8 | 1.883 | 28 | positive |
| **Diuron** | 233 | 72.1 | 6.82 | 24 | positive |  | 222.05 | 204.1 | 1.883 | 14 | positive |
|  | 235 | 72.1 | 6.82 | 26 | positive | **Sebuthylazine** | 230.2 | 131.9 | 7.29 | 28 | positive |
| **Epoxiconazole** | 330.15 | 101.1 | 7.94 | 56 | positive |  | 230.2 | 174 | 7.29 | 16 | positive |
|  | 330.15 | 121.1 | 7.94 | 24 | positive | **Simazine** | 202.1 | 124.1 | 5.92 | 20 | positive |
| **Ethofumesate** | 287 | 120.9 | 7.34 | 16 | positive |  | 202.1 | 132 | 5.92 | 20 | positive |
|  | 287 | 258.9 | 7.34 | 8 | positive | **Simazine-2-hydroxy** | 184.11 | 97.1 | 3.98 | 28 | positive |
| **Hexazinone** | 253.2 | 71.1 | 5.94 | 36 | positive |  | 184.11 | 114.1 | 3.98 | 20 | positive |
|  | 253.2 | 171.1 | 5.94 | 16 | positive | **Tebuconazole** | 308.1 | 70.1 | 8.26 | 26 | positive |
| **Chloridazon** | 222 | 91.8 | 4.38 | 28 | positive |  | 308.1 | 125 | 8.26 | 44 | positive |
|  | 222 | 104.1 | 4.38 | 24 | positive | **Terbuthylazine** | 230.1 | 96 | 7.45 | 28 | positive |
| **Chloridazon-desphenyl** | 146 | 65.8 | 1.47 | 44 | positive |  | 230.1 | 104 | 7.45 | 40 | positive |
|  | 146 | 100.7 | 1.47 | 16 | positive |  | 230.1 | 174.1 | 7.45 | 16 | positive |
|  | 146 | 116.9 | 1.47 | 24 | positive | **Terbuthylazine desethyl** | 202.1 | 79 | 6.14 | 36 | positive |
| **Chloridazon-m.-desphenyl** | 159.8 | 87.7 | 2.06 | 36 | positive |  | 202.1 | 146 | 6.14 | 16 | positive |
|  | 159.8 | 116.8 | 2.06 | 28 | positive | **Terbuthylazine-desethyl-2-hydroxy** | 184.1 | 86.2 | 3.76 | 28 | positive |
| **Chlorotoluron** | 213.1 | 46.1 | 6.48 | 16 | positive |  | 184.1 | 128.1 | 3.76 | 12 | positive |
|  | 213.1 | 72 | 6.48 | 20 | positive | **Terbuthylazine-2-hydroxy** | 212.2 | 86 | 6.14 | 28 | positive |
| **Chlorpyrifos** | 349.9 | 96.7 | 9.26 | 40 | positive |  | 212.2 | 156.1 | 6.14 | 16 | positive |
|  | 349.9 | 197.8 | 9.26 | 24 | positive | **Terbutryn** | 242.15 | 186.1 | 7.98 | 20 | positive |
|  | 351.9 | 199.9 | 9.26 | 20 | positive | **Thiophanate-methyl** | 343 | 151 | 5.87 | 20 | positive |
| **Chlorpyrifos-methyl** | 321.9 | 125 | 8.69 | 20 | positive |  | 343 | 310.9 | 5.87 | 8 | positive |
|  | 323.9 | 125 | 8.69 | 22 | positive | **Triadimefon** | 294.1 | 69.1 | 7.6 | 20 | positive |
| **Isoproturon** | 207.1 | 72.1 | 6.73 | 28 | positive |  | 294.1 | 197.1 | 7.6 | 14 | positive |
|  | 207.1 | 165.1 | 6.73 | 12 | positive |  | 294.1 | 225.1 | 7.6 | 12 | positive |
| **Isoproturon-didesmethyl** | 178.9 | 94.1 | 6.34 | 24 | positive |  |  |  |  |  |  |
|  | 178.9 | 137 | 6.34 | 12 | positive |  |  |  |  |  |  |

**Table S4: Characteristics of the method for 16 PAHs and 6 PCBs determined by GC-MS/MS.**

| **PAH** | **LOD (ng/g)** | **LOQ (ng/g)** | **Calibration range (ng/ml)** | **Recovery (LOQ levels) ± RSD (%)** | **Recovery (100 ng/ml) ± RSD (%)** | **R^2^** |
| --- | --- | --- | --- | --- | --- | --- |
| **Acenaphthene** | 2 | 6 | 5 - 200 | 82.4 ± 2.83 | 85 ± 1.52 | 0.999 |
| **Acenaphthylene** | 1 | 2 | 1 - 200 | 84.2 ± 3.41 | 87 ± 2.48 | 0.999 |
| **Anthracene** | 1 | 2 | 1 - 200 | 93.1 ± 2.33 | 95 ± 1.96 | 0.998 |
| **Benz[a]anthracene** | 0.2 | 1 | 0.5 - 200 | 100 ± 3.59 | 113 ± 2.93 | 0.999 |
| **Benzo[a]pyrene** | 1 | 3 | 5 - 200 | 103 ± 3.84 | 108 ± 3.45 | 0.997 |
| **Benzo[b]fluoranthene** | 1 | 2 | 2 - 200 | 101 ± 1.89 | 103 ± 1.22 | 0.999 |
| **Benzo[g.h.i]perylene** | 1 | 5 | 2 - 200 | 109 ± 2.32 | 117 ± 2.71 | 0.999 |
| **Benzo[k]fluoranthene** | 0.4 | 1 | 2 - 200 | 83.8 ± 1.29 | 82 ± 0.94 | 0.998 |
| **Dibenz[a.h]anthracene** | 1 | 2 | 5 - 200 | 105 ± 4.28 | 110 ± 3.88 | 0.997 |
| **Phenanthrene** | 2 | 6 | 5 - 200 | 104 ± 8.39 | 109 ± 7.97 | 0.999 |
| **Fluoranthene** | 0.04 | 0.1 | 0.5 - 200 | 102 ± 6.56 | 106 ± 5.67 | 0.999 |
| **Fluorene** | 1 | 2 | 2 - 200 | 89.7 ± 2.78 | 92 ± 1.89 | 0.999 |
| **Chrysene** | 1 | 2 | 0.5 - 200 | 98.5 ± 4.57 | 101 ± 5.72 | 0.999 |
| **Indeno[1.2.3-cd]pyrene** | 0.5 | 2 | 2 - 200 | 106 ± 2.8 | 106 ± 2.87 | 0.997 |
| **Naphthalene** | 0.1 | 0.5 | 0.5 - 200 | 87.3 ± 4.39 | 83 ± 4.96 | 0.999 |
| **Pyrene** | 0.2 | 1 | 0.5 - 200 | 103 ± 6.14 | 108 ± 5.48 | 0.999 |

| **PCB** | **LOD (ng/g)** | **LOQ (ng/g)** | **Calibration range (ng/ml)** | **Recovery (LOQ levels) ± RSD (%)** | **Recovery (100 ng/ml) ± RSD (%)** | **R^2^** |
| --- | --- | --- | --- | --- | --- | --- |
| **2.2'.3.4.4'.5.5'-Heptachlorobiphenyl (PCB180)** | 0.1 | 1 | 0.5 – 200 | 111 ± 3.20 | 118 ± 5.35 | 0.999 |
| **2.2'.3.4.4'.5'-Hexachlorobiphenyl (PCB 138)** | 0.3 | 1 | 0.5 - 200 | 104 ± 6.33 | 115 ± 10.8 | 0.997 |
| **2.2'.4.4'.5.5'-Hexachlorobiphenyl (PCB 153)** | 0.3 | 1 | 0.5 - 200 | 114 ± 1.76 | 119 ± 4.52 | 0.997 |
| **2.2'.4.5.5'-Pentachlorobiphenyl (PCB 101)** | 0.3 | 1 | 0.5 - 200 | 106 ± 5.68 | 112 ± 6.55 | 0.997 |
| **2.2'.5.5'-Tetrachlorobiphenyl (PCB 52)** | 0.3 | 1 | 0.5 - 200 | 110 ± 6.38 | 97 ± 7.75 | 0.997 |
| **2.4.4'-Trichlorobiphenyl (PCB 28)** | 0.3 | 1 | 0.5 - 200 | 111 ± 3.38 | 119 ± 8.43 | 0.998 |

**Table S5: The accuracy of the proposed method.**

| **PAH** | **CRM values**  **c (mg/kg)** | **Obtained values**  **c (mg/kg)** | **Recovery (%)** |
| --- | --- | --- | --- |
| **Naphthalene** | 0.22 ± 0.11 | 0.19 ± 0.02 | 88.1 |
| **Acenaphthene** | 0.07 ± 0.02 | 0.06 ± 0.01 | 83.5 |
| **Fluorene** | 0.12 ± 0.04 | 0.12 ± 0.01 | 99.2 |
| **Phenanthrene** | 1.04 ± 0.3 | 1.01 ± 0.10 | 97.2 |
| **Anthracene** | 0.36 ± 0.11 | 0.36 ± 0.04 | 99.7 |
| **Fluoranthene** | 1.79 ± 0.35 | 1.44 ± 0.14 | 80.4 |
| **Pyrene** | 1.48 ± 0.5 | 1.28 ± 0.13 | 86.5 |
| **Benz[a]anthracene** | 0.83 ± 0.18 | 0.71 ± 0.07 | 86.0 |
| **Chrysene** | 0.83 ± 0.16 | 0.75 ± 0.08 | 90.7 |
| **Benzo[b]fluoranthene** | 0.82 ± 0.19 | 0.83 ± 0.08 | 101 |
| **Benzo[k]fluoranthene** | 0.5 ± 0.08 | 0.46 ± 0.05 | 91.9 |
| **Benzo[a]pyrene** | 0.65 ± 0.14 | 0.54 ± 0.05 | 82.9 |
| **Indeno[1,2,3-cd]pyrene** | 0.37 ± 0.14 | 0.32 ± 0.03 | 87.1 |
| **Dibenz[a,h]anthracene** | 0.13 ± 0.05 | 0.13 ± 0.01 | 98.3 |
| **Benzo[g,h,i]perylene** | 0.36 ± 0.13 | 0.35 ± 0.04 | 98.4 |

**Pesticides and pharmaceuticals**

The characteristics of the method are summarized in Table S6. The calibration curve was constructed using nine concentration levels (1, 5, 10, 25, 50, 75, 100, 250, 500 ng/L). The external calibration method was used to calculate the concentration of pesticides and pharmaceuticals in sediments. Satisfactory correlation coefficients (0.997–0.999) were obtained for all compounds, demonstrating that the method is linear across the entire tested calibration range. The recovery was expressed as the average of seven spiked samples with all the analytes at the final concentration levels of LOQ and 100 ng/L for sediment samples after subtracting the blank sample. The measurement uncertainty for pesticides was 22 % and for pharmaceuticals 21 %. The accuracy and precision of all analytes were evaluated by analyzing seven spiked samples with the mixed standard at a resulting pesticide/pharmaceutical concentration in the sediment extract of 10 ng/L and 100 ng/L after a 5x dilution.

**Table S6: Characteristics of the method for pesticides and pharmaceuticals determined by LC-MS/MS.**

| **Analytes** | **LOD (ng/g)** | **LOQ (ng/g)** | **Calibration range (ng/ml)** | **Recovery (LOQ levels) ± RSD (%)** | **Recovery (100 ng/ml) ± RSD (%)** | **R^2^** | **CAS** | **Type ^a^** |
| --- | --- | --- | --- | --- | --- | --- | --- | --- |
| **2.4.5-T** | 0.06 | 0.2 | 10-500 | 83.7 ± 15.3 | 100 ± 9.44 | 0.998 | 93-76-5 | h |
| **2.4-D** | 0.06 | 0.2 | 10-500 | 90.7 ± 9.82 | 107 ± 6.45 | 0.999 | 94-75-7 | h |
| **2.6-dichlorobenzamide** | 0.03 | 0.1 | 5-500 | 88.6 ± 5.08 | 101 ± 2.42 | 0.999 | 2008-58-4 | h |
| **Acetochlor** | 0.30 | 1 | 50-500 | 107 ± 4.77 | 108 ± 2.20 | 0.998 | 34256-82-1 | h |
| **Acetochlor ESA** | 0.30 | 1 | 50-500 | 106 ± 15.0 | 73.1 ± 14.3 | 0.998 | 187022-11-3 | h |
| **Acetochlor OA** | 0.30 | 1 | 50-500 | 107 ± 12.6 | 94.8 ± 11.4 | 0.999 | 500-72-1 | h |
| **Alachlor** | 0.30 | 1 | 50-500 | 93.9 ± 9.86 | 101 ± 5.93 | 0.997 | 15972-60-8 | h |
| **Alachlor ESA** | 0.30 | 1 | 50-500 | 90.0 ± 12.9 | 98.1 ± 15.8 | 0.997 | 142363-53-9 | h |
| **Alachlor OA** | 0.30 | 1 | 50-500 | 98.1 ± 15.4 | 90.1 ± 11.1 | 0.998 | 171262-17-2 | h |
| **Ametryn** | 0.03 | 0.1 | 5-500 | 97.8 ± 12.0 | 112 ± 11.7 | 0.999 | 834-12-8 | h |
| **Atrazine** | 0.03 | 0.1 | 5-500 | 93.1 ± 6.26 | 105 ± 3.18 | 0.999 | 1912-24-9 | h |
| **Atrazine-2-hydroxy** | 0.03 | 0.1 | 5-500 | 107 ± 15.2 | 101 ± 7.29 | 0.999 | 2163-68-0 | h |
| **Atrazine-desethyl** | 0.03 | 0.1 | 5-500 | 97.7 ± 3.45 | 95.1 ± 4.22 | 0.999 | 6190-65-4 | h |
| **Atrazine-desethyl-desisopropyl** | 0.15 | 0.5 | 25-500 | 82.8 ± 15.7 | 107 ± 4.95 | 0.999 | 3397-62-4 | h |
| **Atrazine-desisopropyl** | 0.06 | 0.2 | 10-500 | 98.0 ± 3.78 | 111 ± 2.94 | 0.998 | 1007-28-9 | h |
| **Azoxystrobin** | 0.03 | 0.1 | 5-500 | 82.3 ± 4.00 | 81.8 ± 1.67 | 0.997 | 131860-33-8 | f |
| **Bentazone** | 0.03 | 0.1 | 5-500 | 93.9 ± 9.02 | 108 ± 1.44 | 0.999 | 25057-89-0 | h |
| **Clomazon** | 0.03 | 0.1 | 5-500 | 95.3 ± 5.89 | 92.3 ± 4.93 | 0.999 | 81777-89-1 | h |
| **Cyanazin** | 0.03 | 0.1 | 5-500 | 94.0 ± 8.26 | 90.8 ± 3.45 | 0.999 | 21725-46-2 | h |
| **Cyproconazol** | 0.03 | 0.1 | 5-500 | 98.3 ± 5.01 | 95.3 ± 6.74 | 0.999 | 94361-06-5 | f |
| **Desmetryn** | 0.03 | 0.1 | 5-500 | 90.1 ± 7.00 | 100 ± 6.60 | 0.999 | 1014-69-3 | h |
| **Diazinon** | 0.03 | 0.1 | 5-500 | 86.0 ± 10.5 | 78.9 ± 8.12 | 0.998 | 333-41-5 | i |
| **Difenoconazol** | 0.03 | 0.1 | 5-500 | 78.9 ± 1.38 | 82.9 ± 7.94 | 0.998 | 119446-68-3 | f |
| **Diflufenican** | 0.15 | 0.5 | 25-500 | 90.1 ± 8.00 | 97.9 ± 8.87 | 0.999 | 83164-33-4 | h |
| **Dichlorprop** | 0.06 | 0.2 | 10-500 | 97.3 ± 7.26 | 92.9 ± 3.98 | 0.999 | 120-36-5 | h |
| **Dimetachlor ESA** | 0.30 | 1 | 50-500 | 86.7 ± 15.4 | 82.3 ± 4.02 | 0.999 | CASID30748 | h |
| **Dimetachlor OA** | 0.03 | 0.1 | 5-500 | 101 ± 15.7 | 81.7 ± 2.59 | 0.998 | 1086384-49-7 | h |
| **Dimethachlor** | 0.15 | 0.5 | 25-500 | 113 ± 9.64 | 87.1 ± 2.53 | 0.997 | 50563-36-5 | h |
| **Dimethenamid** | 0.03 | 0.1 | 5-500 | 98.5 ± 2.44 | 81.5 ± 5.01 | 0.997 | 163515-14-8 | h |
| **Dimethoate** | 0.06 | 0.2 | 10-500 | 89.9 ± 10.9 | 85.8 ± 4.98 | 0.998 | 60-51-5 | i |
| **Diuron** | 0.03 | 0.1 | 5-500 | 88.3 ± 9.93 | 89.0 ± 2.98 | 0.998 | 330-54-1 | h |
| **Epoxiconazole** | 0.30 | 1 | 50-500 | 106 ± 7.00 | 101 ± 5.47 | 0.996 | 135319-73-2 | f |
| **Ethofumesate** | 0.03 | 0.1 | 5-500 | 102 ± 4.77 | 100 ± 5.13 | 0.998 | 26225-79-6 | h |
| **Ethoprophos** | 0.06 | 0.2 | 10-500 | 102 ± 4.77 | 100 ± 5.13 | 0.998 | 13194-48-4 | i |
| **Fenuron** | 0.15 | 0.5 | 25-500 | 80.9 ± 2.75 | 89.4 ± 11.0 | 0.997 | 101-42-8 | h |
| **Fluopicolide** | 0.03 | 0.1 | 5-500 | 88.0 ± 10.1 | 96.1 ± 8.84 | 0.997 | 239110-15-7 | f |
| **Hexazinone** | 0.03 | 0.1 | 5-500 | 91.1 ± 5.12 | 103 ± 3.84 | 0.999 | 51235-04-2 | h |
| **Chloridazon** | 0.06 | 0.2 | 10-500 | 79.8 ± 16.4 | 91.8 ± 5.57 | 0.997 | 1698-60-8 | h |
| **Chloridazon-desphenyl** | 0.03 | 0.1 | 5-500 | 88.4 ± 3.67 | 91.8 ± 1.74 | 0.999 | 6339-19-1 | h |
| **Chloridazon-methyl-desphenyl** | 0.03 | 0.1 | 5-500 | 75.7 ± 14.7 | 87.0 ± 7.04 | 0.999 | 17254-80-7 | h |
| **Chlorotoluron** | 0.06 | 0.2 | 10-500 | 69.1 ± 7.00 | 75.3 ± 0.16 | 0.997 | 15545-48-9 | h |
| **Chlorotoluron-desmethyl** | 0.03 | 0.1 | 5-500 | 86.2 ± 12.3 | 74.8 ± 6.91 | 0.998 | 22175-22-0 | h |
| **Chlorpyrifos** | 0.03 | 0.1 | 5-500 | 78.7 ± 13.9 | 82.2 ± 2.74 | 0.999 | 2921-88-2 | i |
| **Chlorpyrifos-methyl** | 0.03 | 0.1 | 5-500 | 77.1 ± 13.3 | 89.9 ± 5.55 | 0.998 | 5598-13-0 | i |
| **Isoproturon** | 0.06 | 0.2 | 10-500 | 111 ± 8.55 | 98.1 ± 5.01 | 0.999 | 34123-59-6 | h |
| **Isoproturon-didesmethyl** | 0.30 | 1 | 50-500 | 69.8 ± 6.41 | 72.9 ± 8.98 | 0.997 | 56046-17-4 | h |
| **Isoproturon-monodesmethyl** | 0.03 | 0.1 | 5-500 | 79.8 ± 2.81 | 78.9 ± 5.02 | 0.997 | 34123-57-4 | h |
| **Linuron** | 0.03 | 0.1 | 5-500 | 92.2 ± 5.57 | 105 ± 6.49 | 0.998 | 330-55-2 | h |
| **MCPA** | 0.06 | 0.2 | 10-500 | 81.8 ± 13.5 | 104 ± 6.39 | 0.998 | 94-74-6 | h |
| **MCPB** | 0.06 | 0.2 | 10-500 | 87.8 ± 15.0 | 110 ± 2.17 | 0.998 | 94-81-5 | h |
| **Mecoprop (MCPP)** | 0.15 | 0.5 | 25-500 | 96.9 ± 8.93 | 97.9 ± 7.34 | 0.998 | 16484-77-8 | h |
| **Metamitron** | 0.15 | 0.5 | 25-500 | 110 ± 10.1 | 99.1 ± 11.9 | 0.998 | 41394-05-2 | h |
| **Metazachlor** | 0.03 | 0.1 | 5-500 | 82.3 ± 11.0 | 96.8 ± 9.52 | 0.999 | 67129-08-2 | h |
| **Metazachlor ESA** | 0.30 | 1 | 50-500 | 86.8 ± 8.10 | 73.1 ± 3.68 | 0.998 | 172960-62-2 | h |
| **Metazachlor OA** | 0.03 | 0.1 | 5-500 | 78.5 ± 5.92 | 86.5 ± 9.77 | 0.999 | 1231244-60-2 | h |
| **Methabenzthiazuron** | 0.30 | 1 | 50-500 | 87.2 ± 11.9 | 102 ± 3.97 | 0.997 | 18691-97-9 | h |
| **Metobromuron** | 0.30 | 1 | 50-500 | 94.3 ± 16.4 | 95.7 ± 7.46 | 0.998 | 3060-89-7 | h |
| **Metolachlor** | 0.06 | 0.2 | 10-500 | 77.2 ± 12.0 | 101 ± 9.90 | 0.998 | 87392-12-9 | h |
| **Metolachlor ESA** | 0.06 | 0.2 | 10-500 | 94.9 ± 5.66 | 94.6 ± 5.49 | 0.998 | 171118-09-5 | h |
| **Metolachlor OA** | 0.30 | 1 | 50-500 | 83.2 ± 16.7 | 103 ± 6.67 | 0.998 | 152019-73-3 | h |
| **Metoxuron** | 0.30 | 1 | 50-500 | 98.5 ± 16.5 | 92.7 ± 8.65 | 0.997 | 19937-59-8 | h |
| **Metribuzin** | 0.06 | 0.2 | 10-500 | 106 ± 13.0 | 99.2 ± 16.5 | 0.999 | 21087-64-9 | h |
| **Metribuzin-desamino** | 0.06 | 0.2 | 10-500 | 93.8 ± 13.7 | 84.6 ± 7.70 | 0.998 | 35045-02-4 | h |
| **Monolinuron** | 0.15 | 0.5 | 25-500 | 101 ± 10.5 | 101 ± 8.39 | 0.997 | 1746-81-2 | h |
| **Napropamid** | 0.06 | 0.2 | 10-500 | 90.1 ± 13.3 | 83.5 ± 6.84 | 0.997 | 15299-99-7 | h |
| **Parathion-methyl** | 0.03 | 0.1 | 5-500 | 82.0 ± 11.4 | 74.0 ± 2.82 | 0.997 | 298-00-0 | i |
| **Pendimethalin** | 0.15 | 0.5 | 25-500 | 78.7 ± 16.3 | 88.0 ± 13.2 | 0.996 | 40487-42-1 | h |
| **Phosalon** | 0.30 | 1 | 50-500 | 73.3 ± 10.4 | 71.4 ± 8.08 | 0.997 | 2310-17-0 | i |
| **Pirimiphos-methyl** | 0.03 | 0.1 | 5-500 | 99.1 ± 5.37 | 111 ± 5.30 | 0.998 | 29232-93-7 | i |
| **Prochloraz** | 0.03 | 0.1 | 5-500 | 90.4 ± 5.14 | 76.5 ± 1.86 | 0.997 | 67747-09-5 | f |
| **Prometryn** | 0.03 | 0.1 | 5-500 | 105 ± 10.7 | 104 ± 3.09 | 0.998 | 7287-19-6 | h |
| **Propachlor** | 0.15 | 0.5 | 25-500 | 90.5 ± 12.4 | 79.1 ± 3.05 | 0.998 | 1918-16-7 | h |
| **Propachlor ESA** | 0.30 | 1 | 50-500 | 85.3 ± 7.22 | 106 ± 1.00 | 0.997 | 947601-88-9 | h |
| **Propazin** | 0.03 | 0.1 | 5-500 | 88.4 ± 3.87 | 107 ± 5.13 | 0.998 | 139-40-2 | h |
| **Propiconazol** | 0.03 | 0.1 | 5-500 | 95.2 ± 6.36 | 100 ± 5.21 | 0.999 | 60207-90-1 | f |
| **Quinmerac** | 0.30 | 1 | 50-500 | 82.0 ± 13.9 | 73.9 ± 1.29 | 0.999 | 90717-03-6 | h |
| **Sebuthylazin** | 0.03 | 0.1 | 5-500 | 95.7 ± 10.1 | 109 ± 4.33 | 0.997 | 7286-69-3 | h |
| **Simazin** | 0.06 | 0.2 | 10-500 | 90.6 ± 5.80 | 112 ± 7.00 | 0.998 | 122-34-9 | h |
| **Simazin-2-hydroxy** | 0.03 | 0.1 | 5-500 | 107 ± 4.40 | 73.1 ± 2.71 | 0.999 | 2599-11-3 | h |
| **Tebuconazol** | 0.03 | 0.1 | 5-500 | 94.6 ± 7.41 | 96.8 ± 2.03 | 0.997 | 107534-96-3 | f |
| **Terbuthylazin desethyl** | 0.03 | 0.1 | 5-500 | 104 ± 12.0 | 90.8 ± 8.46 | 0.999 | 30125-63-4 | h |
| **Terbuthylazin-desethyl-2-hydroxy** | 0.03 | 0.1 | 5-500 | 102 ± 6.60 | 98.0 ± 2.48 | 0.998 | 66753-06-8 | h |
| **Terbuthylazine** | 0.03 | 0.1 | 5-500 | 97.4 ± 7.68 | 87.5 ± 10.9 | 0.998 | 5915-41-3 | h |
| **Terbuthylazine-2-hydroxy** | 0.03 | 0.1 | 5-500 | 91.8 ± 3.21 | 75.5 ± 5.05 | 0.999 | 66753-07-9 | h |
| **Terbutryn** | 0.03 | 0.1 | 5-500 | 104 ± 10.4 | 107 ± 6.27 | 0.999 | 886-50-0 | h |
| **Thiophanate-methyl** | 0.06 | 0.2 | 10-500 | 70.1 ± 3.61 | 72.8 ± 4.98 | 0.997 | 23564-05-8 | f |
| **Triadimefon** | 0.03 | 0.1 | 5-750 | 81.6 ± 6.13 | 88.2 ± 4.57 | 0.998 | 43121-43-3 | f |

h = herbicide; f = fungicide; i = insecticide

| **Pharmaceuticals** | **LOD (ng/g)** | **LOQ (ng/g)** | **Calibration range (ng/l)** | **Recovery (100 ng/L) ± RSD (%)** | **Recovery (low concentration) ± RSD (%)** | **R^2^** |
| --- | --- | --- | --- | --- | --- | --- |
| **3-hydroxycarbamazepine** | 0.04 | 0.14 | 10 - 500 | 78.9 ± 5.71 | 77.8 ± 17.7 | 0.997 |
| **Acebutolol** | 0.04 | 0.15 | 10 - 500 | 72.3 ± 4.30 | 82.0 ± 5.29 | 0.997 |
| **Caffeine** | 0.04 | 0.15 | 10 - 500 | 76.0 ± 5.82 | 69.7 ± 17.2 | 0.997 |
| **Carbamazepine** | 0.06 | 0.19 | 10 - 500 | 74.4 ± 8.52 | 119 ± 5.85 | 0.997 |
| **Clarithromycin** | 0.03 | 0.11 | 10 - 500 | 74.0 ± 2.00 | 70.0 ± 8.00 | 0.998 |
| **Diclofenac** | 0.05 | 0.17 | 10 - 500 | 79.6 ± 2.74 | 94.8 ± 13.0 | 0.997 |
| **Ketoprofen** | 0.04 | 0.14 | 10 - 500 | 71.9 ± 7.20 | 76.6 ± 7.10 | 0.998 |
| **Metoprolol** | 0.04 | 0.15 | 10 - 500 | 82.3 ± 4.30 | 87.0 ± 5.29 | 0.997 |
| **N-acetylsulfamethoxazole** | 0.05 | 0.17 | 10 - 500 | 82.6 ± 3.62 | 111 ± 7.44 | 0.997 |
| **N-acetylsulfapyridine** | 0.04 | 0.13 | 10 - 500 | 75.6 ± 0.36 | 70.6 ± 22.8 | 0.999 |
| **Phenazone** | 0.09 | 0.29 | 10 - 500 | 91.4 ± 5.09 | 121 ± 28.5 | 0.999 |
| **Propyphenazone** | 0.05 | 0.17 | 10 - 500 | 89.3 ± 8.53 | 96.3 ± 13.4 | 0.998 |
| **Sulfadiazin** | 0.04 | 0.13 | 10 - 500 | 76.4 ± 6.15 | 101 ± 4.07 | 0.997 |
| **Sulfamerazine** | 0.08 | 0.26 | 10 - 500 | 70.1 ± 0.89 | 118 ± 1.71 | 0.997 |
| **Sulfamethoxazole** | 0.02 | 0.05 | 10 - 500 | 68.7 ± 0.36 | 120 ± 5.41 | 0.997 |
| **Sulfapyridine** | 0.03 | 0.09 | 10 - 500 | 72.5 ± 2.87 | 98.9 ± 4.54 | 0.998 |
| **Tramadol** | 0.03 | 0.11 | 10 - 500 | 80.4 ± 1.38 | 87.0 ± 0.98 | 0.997 |

**List of all analytes**

| **Polycyclic aromatic hydrocarbons (PAHs)** | | |
| --- | --- | --- |
| Naphthalene | Acenaphthylene | Acenaphthene |
| Fluorene | Phenanthrene | Anthracene |
| Fluoranthene | Pyrene | Benz[a]anthracene |
| Chrysene | Benzo[b]fluoranthene | Benzo[k]fluoranthene |
| Benzo[a]pyrene | Indeno[1,2,3-cd]pyrene | Dibenz[a,h]anthracene |
| Benzo[g,h,i]perylene |  |  |

| **Polychlorinated byphenyls (PCBs)** | | |
| --- | --- | --- |
| 2,4,4'-Trichlorobifenyl (PCB 28) | 2,2',5,5'-Tetrachlorobifenyl (PCB 52) | 2,2',4,5,5'-Pentachlorobifenyl (PCB 101) |
| 2,2',3,4,4',5'-Hexachlorobifenyl (PCB 138) | 2,2',4,4',5,5'-Hexachlorobifenyl (PCB 153) | 2,2',3,4,4',5,5'-Heptachlorobifenyl (PCB 180) |

| **Pesticides** | | |
| --- | --- | --- |
| 2,4,5-T | 2,4-D | 2,6-dichlorobenzamide |
| Acetochlor | Acetochlor ESA | Acetochlor OA |
| Alachlor | Alachlor ESA | Alachlor OA |
| Ametryn | Atrazine | Atrazine-2-hydroxy |
| Atrazine-desethyl | Atrazine-desethyl-desisopropyl | Atrazine-desisopropyl |
| Azoxystrobin | Bentazone | Clomazone |
| Cyanazine | Cyproconazole | Desmetryn |
| Diazinon | Difenoconazole | Diflufenican |
| Dichlorprop | Dimethachlor ESA | Dimethachlor OA |
| Dimethachlor | Diuron | Epoxiconazole |
| Ethofumesate | Hexazinone | Chloridazon |
| Chloridazon-desphenyl | Chloridazon-m.-desphenyl | Chlorotoluron |
| Chlorpyrifos | Chlorpyrifos-methyl | Isoproturon |
| Isoproturon-didesmethyl | Isoproturon-monodesmethyl | Linuron |
| MCPA | MCPB | Mecoprop |
| Metamitron | Metazachlor | Metazachlor ESA |
| Metazachlor OA | Methabenzthiazuron | Metobromuron |
| Metolachlor | Metolachlor ESA | Metolachlor OA |
| Metoxuron | Metribuzin | Metribuzin-desamino |
| Monolinuron | Napropamide | Parathion-methyl |
| Pendimethalin | Phosalone | Pirimiphos-methyl |
| Prochloraz | Prometryn | Propachlor |
| Propachlor ESA | Propazine | Propiconazole |
| Quinmerac | Sebuthylazine | Simazine |
| Simazine-2-hydroxy | Tebuconazole | Terbuthylazine desethyl |
| Terbuthylazine-desethyl-2-hydroxy | Terbuthylazine | Terbuthylazine-2-hydroxy |
| Terbutryn | Thiophanate-methyl | Triadimefon |

| **Pharmaceuticals + caffeine** | | |
| --- | --- | --- |
| 3-hydroxycarbamazepine | Acebutolol | Caffeine |
| Carbamazepine | Clarithromycin | Diclofenac |
| Ketoprofen | Metoprolol | N-acetylsulfamethoxazole |
| N-acetylsulfapyridine | Phenazone | Propyphenazone |
| Sulfadiazin | Sulfamerazine | Sulfamethoxazole |
| Sulfapyridine | Tramadol |  |

**Supplementary Material 2: Results**

**Table S7: Grain size data for all measured samples showing the percentage of fractions and basic grain size parameters (D_10_, D_50_, and D_90_)**

| **Bohumín area - channel bars** | | | | | | | | | | | |
| --- | --- | --- | --- | --- | --- | --- | --- | --- | --- | --- | --- |
| Sample | Clay (%) | Silt (%) | | Sand (%) | | | | | Grain size parameters | | |
|  |  |  |  | Very fine | Fine | Medium | Coarse | Very coarse |  |  |  |
|  | μm | | | | | | | | | | |
|  | <4 | 4-32 | 32-63 | 63-125 | 125-250 | 250-500 | 500-1000 | 1000-2000 | D_10_ (μm) | D_50_ (μm) | D_90_ (μm) |
| ODB1 | 13.4 | 50.4 | 21.3 | 13.2 | 1.7 | 0.0 | 0.0 | 0.0 | 2.9 | 20.8 | 75.8 |
| ODB2 | 14.1 | 49.6 | 20.1 | 14.6 | 1.6 | 0.0 | 0.0 | 0.0 | 2.8 | 19.9 | 78.4 |
| ODB3 | 13.6 | 45.9 | 16.7 | 15.9 | 7.8 | 0.1 | 0.0 | 0.0 | 2.9 | 22.2 | 113.5 |
| ODB4 | 10.3 | 38.4 | 21.5 | 17.3 | 10.9 | 1.6 | 0.0 | 0.0 | 3.8 | 31.3 | 117.8 |
| ODB7 | 12.3 | 48.7 | 25.3 | 13.0 | 0.7 | 0.0 | 0.0 | 0.0 | 3.1 | 23.5 | 70.8 |
| ODB8 | 9.1 | 43.2 | 21.6 | 15.4 | 7.9 | 2.8 | 0.0 | 0.0 | 4.5 | 28.9 | 131.5 |
| ODB9 | 13.1 | 49.2 | 22.8 | 14.1 | 0.9 | 0.0 | 0.0 | 0.0 | 3.1 | 21.5 | 73.9 |
| ODB10 | 10.5 | 51.5 | 22.9 | 14.1 | 1.1 | 0.0 | 0.0 | 0.0 | 3.8 | 22.4 | 74.7 |
| ODB11 | 15.9 | 56.9 | 21.4 | 5.8 | 0.0 | 0.0 | 0.0 | 0.0 | 2.6 | 16.9 | 52.3 |
| ODB13 | 12.2 | 52.2 | 25.5 | 10.1 | 0.1 | 0.0 | 0.0 | 0.0 | 3.3 | 21.5 | 62.3 |
| ODB14 | 14.9 | 50.1 | 22.8 | 11.9 | 0.4 | 0.0 | 0.0 | 0.0 | 2.6 | 20.1 | 67.3 |

| **Bohumín area – abandoned meander, CHAL1 core** | | | | | | | | | | | |
| --- | --- | --- | --- | --- | --- | --- | --- | --- | --- | --- | --- |
| Depth (cm) | Clay (%) | Silt (%) | | Sand (%) | | | | | Grain size parameters | | |
|  |  |  |  | Very fine | Fine | Medium | Coarse | Very coarse |  |  |  |
|  | μm | | | | | | | | | | |
|  | <4 | 4-32 | 32-63 | 63-125 | 125-250 | 250-500 | 500-1000 | 1000-2000 | D_10_ (μm) | D_50_ (μm) | D_90_ (μm) |
| 8 | 18.9 | 63.2 | 17.3 | 0.7 | 0.0 | 0.0 | 0.0 | 0.0 | 2.6 | 15.9 | 37.8 |
| 17 | 19.3 | 63.3 | 16.6 | 0.8 | 0.0 | 0.0 | 0.0 | 0.0 | 2.5 | 15.3 | 37.5 |
| 25 | 18.1 | 69.1 | 12.7 | 0.1 | 0.0 | 0.0 | 0.0 | 0.0 | 2.8 | 14.2 | 33.4 |
| 33 | 19.1 | 69.4 | 11.5 | 0.0 | 0.0 | 0.0 | 0.0 | 0.0 | 2.7 | 13.6 | 32.3 |
| 42 | 20.9 | 70.3 | 8.7 | 0.0 | 0.0 | 0.0 | 0.0 | 0.0 | 2.4 | 12.3 | 30.1 |
| 50 | 22.1 | 70.9 | 7.1 | 0.0 | 0.0 | 0.0 | 0.0 | 0.0 | 2.4 | 11.1 | 28.5 |
| 58 | 17.2 | 62.7 | 19.0 | 1.1 | 0.0 | 0.0 | 0.0 | 0.0 | 2.8 | 16.9 | 39.7 |
| 66 | 16.9 | 59.3 | 21.0 | 2.8 | 0.0 | 0.0 | 0.0 | 0.0 | 2.8 | 17.7 | 44.5 |
| 75 | 14.2 | 57.6 | 20.0 | 7.8 | 0.4 | 0.0 | 0.0 | 0.0 | 3.2 | 19.1 | 56.4 |
| 91 | 19.8 | 67.8 | 12.3 | 0.1 | 0.0 | 0.0 | 0.0 | 0.0 | 2.6 | 13.6 | 33.1 |
| 105 | 18.8 | 60.2 | 17.7 | 3.3 | 0.0 | 0.0 | 0.0 | 0.0 | 2.6 | 16.3 | 42.9 |
| 110 | 18.3 | 63.1 | 17.5 | 1.0 | 0.0 | 0.0 | 0.0 | 0.0 | 2.7 | 15.5 | 38.7 |
| 115 | 15.8 | 60.4 | 17.5 | 4.3 | 0.4 | 1.0 | 0.7 | 0.0 | 3.1 | 17.8 | 48.4 |
| 120 | 16.0 | 60.1 | 20.4 | 3.5 | 0.0 | 0.0 | 0.0 | 0.0 | 2.9 | 17.2 | 45.6 |
| 125 | 17.8 | 67.6 | 14.3 | 0.3 | 0.0 | 0.0 | 0.0 | 0.0 | 2.8 | 14.7 | 35.1 |
| 130 | 17.3 | 66.4 | 15.8 | 0.5 | 0.0 | 0.0 | 0.0 | 0.0 | 2.9 | 15.3 | 36.4 |
| 135 | 16.9 | 67.6 | 15.1 | 0.4 | 0.0 | 0.0 | 0.0 | 0.0 | 2.9 | 15.1 | 35.7 |

| **Bohumín area – abandoned meander, CHAL2 core** | | | | | | | | | | | |  |
| --- | --- | --- | --- | --- | --- | --- | --- | --- | --- | --- | --- | --- |
| Depth (cm) | Clay (%) | Silt (%) | | Sand (%) | | | | | Grain size parameters | | |  |
|  |  |  |  | Very fine | Fine | Medium | Coarse | Very coarse |  |  |  |  |
|  | μm | | | | | | | | | | |  |
|  | <4 | 4-32 | 32-63 | 63-125 | 125-250 | 250-500 | 500-1000 | 1000-2000 | D_10_ (μm) | D_50_ (μm) | D_90_ (μm) |  |
| 3 | 6.4 | 19.9 | 7.6 | 6.7 | 24.5 | 34.5 | 0.4 | 0.0 | 6.3 | 191.4 | 358.9 |  |
| 7 | 7.5 | 26.9 | 9.6 | 6.8 | 18.4 | 29.4 | 1.5 | 0.0 | 5.1 | 117.6 | 377.1 |  |
| 10 | 6.0 | 20.7 | 9.4 | 9.2 | 37.4 | 17.3 | 0.0 | 0.0 | 6.4 | 144.1 | 284.5 |  |
| 14 | 5.8 | 20.4 | 6.7 | 4.1 | 15.6 | 45.3 | 2.0 | 0.0 | 6.8 | 237.8 | 410.7 |  |
| 17 | 9.0 | 29.0 | 9.8 | 9.1 | 22.3 | 20.8 | 0.0 | 0.0 | 4.3 | 74.8 | 311.3 |  |
| 21 | 16.9 | 31.2 | 0.0 | 1.3 | 11.2 | 10.1 | 14.4 | 14.7 | 2.9 | 134.8 | 1099.8 |  |
| 28 | 11.5 | 33.7 | 10.8 | 8.8 | 10.4 | 19.6 | 4.3 | 0.8 | 3.6 | 38.5 | 406.9 |  |
| 31 | 13.9 | 45.7 | 13.0 | 8.5 | 11.5 | 7.3 | 0.0 | 0.0 | 3.3 | 23.5 | 218.8 |  |
| 34 | 2.9 | 13.0 | 7.3 | 5.6 | 23.8 | 42.4 | 4.7 | 0.2 | 21.3 | 238.8 | 442.2 |  |
| 38 | 7.5 | 22.0 | 6.9 | 7.0 | 23.3 | 33.0 | 0.4 | 0.0 | 4.9 | 176.6 | 358.9 |  |
| 41 | 8.6 | 28.4 | 11.1 | 8.5 | 18.8 | 23.7 | 0.9 | 0.0 | 4.5 | 76.1 | 354.3 |  |
| 45 | 4.1 | 15.7 | 7.2 | 6.2 | 20.1 | 29.5 | 11.2 | 5.9 | 14.2 | 232.6 | 875.8 |  |
| 48 | 9.0 | 25.6 | 8.0 | 7.4 | 17.8 | 31.6 | 0.7 | 0.0 | 4.3 | 126.3 | 366.7 |  |
| 52 | 14.2 | 39.5 | 9.9 | 4.4 | 14.9 | 17.1 | 0.0 | 0.0 | 3.2 | 27.2 | 296.7 |  |
| 55 | 2.3 | 10.1 | 5.6 | 3.2 | 13.6 | 53.8 | 11.3 | 0.0 | 25.5 | 316.1 | 512.2 |  |
| 58 | 4.5 | 14.6 | 4.9 | 3.4 | 20.8 | 49.9 | 2.0 | 0.0 | 13.1 | 256.1 | 411.6 |  |
| 62 | 4.9 | 18.9 | 8.6 | 5.9 | 24.4 | 36.5 | 0.8 | 0.0 | 10.8 | 199.8 | 372.3 |  |
| 65 | 2.7 | 12.3 | 6.8 | 3.7 | 29.9 | 43.6 | 1.1 | 0.0 | 21.9 | 233.6 | 386.8 |  |
| 69 | 3.76 | 14.12 | 6.39 | 4.22 | 32.91 | 38.20 | 0.40 | 0.0 | 16.9 | 216.5 | 361.4 |  |
| 72 | 7.40 | 26.58 | 9.45 | 8.33 | 28.48 | 19.75 | 0.02 | 0.0 | 5.1 | 112.9 | 300.3 |  |
| 76 | 9.75 | 29.88 | 5.38 | 12.58 | 15.13 | 9.64 | 11.83 | 5.78 | 4.1 | 85.5 | 872.4 |  |
| 79 | 8.26 | 28.16 | 9.53 | 10.47 | 5.24 | 0.81 | 34.52 | 3.01 | 4.6 | 84.5 | 865.1 |  |
| 83 | 16.79 | 54.06 | 19.68 | 9.25 | 0.22 | 0.0 | 0.0 | 0.0 | 2.8 | 18.9 | 60.6 |  |
| 86 | 16.49 | 54.74 | 19.67 | 8.74 | 0.36 | 0.0 | 0.0 | 0.0 | 2.9 | 19.5 | 59.2 |  |
| 93 | 10.22 | 37.51 | 15.75 | 20.69 | 15.78 | 0.05 | 0.0 | 0.0 | 3.9 | 33.3 | 145.8 |  |
| **Strážnické Pomoraví area - channel bars** | | | | | | | | | | | | |
| Sample | Clay (%) | Silt (%) | | Sand (%) | | | | | Grain size parameters | | | |
|  |  |  |  | Very fine | Fine | Medium | Coarse | Very coarse |  |  |  |  |
|  | μm | | | | | | | | | | | |
|  | <4 | 4-32 | 32-63 | 63-125 | 125-250 | 250-500 | 500-1000 | 1000-2000 | D_10_ (μm) | D_50_ (μm) | D_90_ (μm) | |
| SP1 | 9.0 | 30.7 | 19.1 | 10.1 | 20.3 | 10.9 | 0.0 | 0.0 | 4.7 | 44.8 | 255.9 | |
| SP2 | 16.0 | 52.7 | 25.0 | 6.2 | 0.0 | 0.0 | 0.0 | 0.0 | 2.4 | 19.7 | 54.2 | |
| SP3 | 10.7 | 52.9 | 30.5 | 5.9 | 0.0 | 0.0 | 0.0 | 0.0 | 3.8 | 24.1 | 54.3 | |
| SP5 | 1.2 | 6.6 | 5.0 | 3.1 | 4.5 | 58.6 | 21.1 | 0.0 | 39.5 | 385.1 | 571.8 | |
| SP6 | 0.3 | 1.8 | 1.2 | 0.7 | 1.5 | 29.1 | 57.6 | 7.8 | 317.9 | 598.6 | 962.7 | |
| SP8 | 3.3 | 13.6 | 11.5 | 10.0 | 37.2 | 24.5 | 0.0 | 0.0 | 20.1 | 171.5 | 313.0 | |
| SP9 | 10.1 | 50.3 | 32.4 | 7.3 | 0.0 | 0.0 | 0.0 | 0.0 | 4.0 | 25.5 | 57.2 | |
| SP12 | 16.2 | 51.6 | 24.6 | 7.7 | 0.0 | 0.0 | 0.0 | 0.0 | 2.4 | 19.7 | 57.0 | |
| SP13 | 12.3 | 36.8 | 18.5 | 17.1 | 14.3 | 1.0 | 0.0 | 0.0 | 3.1 | 32.1 | 153.9 | |
| SP15 | 0.4 | 1.7 | 0.8 | 0.1 | 2.1 | 3.6 | 71.2 | 20.0 | 525.9 | 809.2 | 1111.4 | |
| SP16 | 8.9 | 20.6 | 6.7 | 10.5 | 39.9 | 13.4 | 0.0 | 0.0 | 22.4 | 165.2 | 275.8 | |

| **Strážnické Pomoraví area – abandoned meander, SPO1 core** | | | | | | | | | | | |
| --- | --- | --- | --- | --- | --- | --- | --- | --- | --- | --- | --- |
| Depth (cm) | Clay (%) | Silt (%) | | Sand (%) | | | | | Grain size parameters | | |
|  |  |  |  | Very fine | Fine | Medium | Coarse | Very coarse |  |  |  |
|  | μm | | | | | | | | | | |
|  | <4 | 4-32 | 32-63 | 63-125 | 125-250 | 250-500 | 500-1000 | 1000-2000 | D_10_ (μm) | D_50_ (μm) | D_90_ (μm) |
| 4 | 11.5 | 59.0 | 26.6 | 2.9 | 0.0 | 0.0 | 0.0 | 0.0 | 3.6 | 21.4 | 47.1 |
| 11 | 10.7 | 59.8 | 27.2 | 2.4 | 0.0 | 0.0 | 0.0 | 0.0 | 3.8 | 22.0 | 46.0 |
| 19 | 11.0 | 59.3 | 27.1 | 2.6 | 0.0 | 0.0 | 0.0 | 0.0 | 3.8 | 21.8 | 46.4 |
| 26 | 12.4 | 62.9 | 23.0 | 1.7 | 0.0 | 0.0 | 0.0 | 0.0 | 3.5 | 19.9 | 42.9 |
| 30 | 11.9 | 58.2 | 26.8 | 3.1 | 0.0 | 0.0 | 0.0 | 0.0 | 3.5 | 21.6 | 47.6 |
| 37 | 10.8 | 59.2 | 27.2 | 2.8 | 0.0 | 0.0 | 0.0 | 0.0 | 3.8 | 22.0 | 47.0 |
| 48 | 12.4 | 57.8 | 26.9 | 3.0 | 0.0 | 0.0 | 0.0 | 0.0 | 3.4 | 21.6 | 47.4 |
| 56 | 11.3 | 58.0 | 26.3 | 4.5 | 0.0 | 0.0 | 0.0 | 0.0 | 3.7 | 21.9 | 49.8 |
| 67 | 12.1 | 59.7 | 25.9 | 2.3 | 0.0 | 0.0 | 0.0 | 0.0 | 3.5 | 21.2 | 45.5 |
| 78 | 10.6 | 57.3 | 28.1 | 4.1 | 0.0 | 0.0 | 0.0 | 0.0 | 3.9 | 22.7 | 49.7 |
| 85 | 12.8 | 56.6 | 27.5 | 3.2 | 0.0 | 0.0 | 0.0 | 0.0 | 3.3 | 21.7 | 48.2 |
| 93 | 12.0 | 58.1 | 23.4 | 5.9 | 0.7 | 0.0 | 0.0 | 0.0 | 3.6 | 21.5 | 51.7 |
| 100 | 10.7 | 54.4 | 29.3 | 5.6 | 0.0 | 0.0 | 0.0 | 0.0 | 3.8 | 23.5 | 53.3 |
| 102 | 11.3 | 54.9 | 30.0 | 3.8 | 0.0 | 0.0 | 0.0 | 0.0 | 3.7 | 23.1 | 50.1 |
| 110 | 11.5 | 52.0 | 31.2 | 5.3 | 0.0 | 0.0 | 0.0 | 0.0 | 3.6 | 24.0 | 53.5 |
| 118 | 14.8 | 59.3 | 24.2 | 1.8 | 0.0 | 0.0 | 0.0 | 0.0 | 3.0 | 19.4 | 44.2 |
| 124 | 13.7 | 56.5 | 26.9 | 2.9 | 0.0 | 0.0 | 0.0 | 0.0 | 3.2 | 20.7 | 47.6 |
| 132 | 13.1 | 54.6 | 25.7 | 6.4 | 0.1 | 0.0 | 0.0 | 0.0 | 3.2 | 21.6 | 53.9 |
| 140 | 8.3 | 45.0 | 27.9 | 8.5 | 9.4 | 0.9 | 0.0 | 0.0 | 4.6 | 29.1 | 128.5 |
| 148 | 15.1 | 58.6 | 24.2 | 2.1 | 0.0 | 0.0 | 0.0 | 0.0 | 3.0 | 19.6 | 44.7 |
| 154 | 9.6 | 48.5 | 29.4 | 10.6 | 1.9 | 0.0 | 0.0 | 0.0 | 4.1 | 26.6 | 69.0 |
| 162 | 11.7 | 55.6 | 29.1 | 3.6 | 0.0 | 0.0 | 0.0 | 0.0 | 3.6 | 22.5 | 49.4 |
| 170 | 10.0 | 58.4 | 28.7 | 2.9 | 0.0 | 0.0 | 0.0 | 0.0 | 4.0 | 22.9 | 47.8 |
| 178 | 16.7 | 72.1 | 11.2 | 0.0 | 0.0 | 0.0 | 0.0 | 0.0 | 2.8 | 15.6 | 32.0 |
| 184 | 8.1 | 46.6 | 25.1 | 15.5 | 4.7 | 0.0 | 0.0 | 0.0 | 4.6 | 28.2 | 97.0 |
| **Strážnické Pomoraví area – abandoned meander, SPO2 core** | | | | | | | | | | | |
| Depth (cm) | Clay (%) | Silt (%) | | Sand (%) | | | | | Grain size parameters | | |
|  |  |  |  | Very fine | Fine | Medium | Coarse | Very coarse |  |  |  |
|  | μm | | | | | | | | | | |
|  | <4 | 4-32 | 32-63 | 63-125 | 125-250 | 250-500 | 500-1000 | 1000-2000 | D_10_ (μm) | D_50_ (μm) | D_90_ (μm) |
| 5 | 8.5 | 58.0 | 30.8 | 2.7 | 0.0 | 0.0 | 0.0 | 0.0 | 4.4 | 24.2 | 47.7 |
| 10 | 10.6 | 57.1 | 24.8 | 7.4 | 0.2 | 0.0 | 0.0 | 0.0 | 3.9 | 22.5 | 55.4 |
| 15 | 12.7 | 63.6 | 23.1 | 0.6 | 0.0 | 0.0 | 0.0 | 0.0 | 3.4 | 20.5 | 40.4 |
| 24 | 9.5 | 50.4 | 30.4 | 9.5 | 0.1 | 0.0 | 0.0 | 0.0 | 4.2 | 25.7 | 61.3 |
| 32 | 11.7 | 51.8 | 26.7 | 9.7 | 0.1 | 0.0 | 0.0 | 0.0 | 3.5 | 23.6 | 61.7 |
| 41 | 12.9 | 56.0 | 26.2 | 4.9 | 0.0 | 0.0 | 0.0 | 0.0 | 3.3 | 21.5 | 50.8 |
| 49 | 11.5 | 47.7 | 26.4 | 12.9 | 1.6 | 0.0 | 0.0 | 0.0 | 3.6 | 25.7 | 74.5 |
| 58 | 12.1 | 53.2 | 25.1 | 9.4 | 0.3 | 0.0 | 0.0 | 0.0 | 3.5 | 22.9 | 61.4 |
| 67 | 11.9 | 52.6 | 28.3 | 7.2 | 0.0 | 0.0 | 0.0 | 0.0 | 3.5 | 23.4 | 56.1 |
| 75 | 15.2 | 56.9 | 24.0 | 3.8 | 0.0 | 0.0 | 0.0 | 0.0 | 2.9 | 20.1 | 48.0 |
| 84 | 13.1 | 58.1 | 26.2 | 2.6 | 0.0 | 0.0 | 0.0 | 0.0 | 3.3 | 21.1 | 46.2 |
| 93 | 13.8 | 56.6 | 26.7 | 2.9 | 0.0 | 0.0 | 0.0 | 0.0 | 3.1 | 21.2 | 47.2 |
| 101 | 9.3 | 37.6 | 16.0 | 24.0 | 13.1 | 0.0 | 0.0 | 0.0 | 4.2 | 34.3 | 135.3 |
| 110 | 8.6 | 44.5 | 28.9 | 16.7 | 1.4 | 0.0 | 0.0 | 0.0 | 4.6 | 29.0 | 80.1 |
| 115 | 5.6 | 22.3 | 12.9 | 31.0 | 28.0 | 0.3 | 0.0 | 0.0 | 9.2 | 84.7 | 168.9 |
| 120 | 11.0 | 53.4 | 30.4 | 5.2 | 0.0 | 0.0 | 0.0 | 0.0 | 3.7 | 23.7 | 53.0 |
| 125 | 9.7 | 47.8 | 24.6 | 10.6 | 0.5 | 0.0 | 4.7 | 2.1 | 4.1 | 26.1 | 91.6 |
| 130 | 8.3 | 45.4 | 32.0 | 14.1 | 0.3 | 0.0 | 0.0 | 0.0 | 4.7 | 28.9 | 70.7 |
| 135 | 11.1 | 56.4 | 28.1 | 4.5 | 0.0 | 0.0 | 0.0 | 0.0 | 3.7 | 22.5 | 50.7 |
| 140 | 12.7 | 55.5 | 26.6 | 5.2 | 0.0 | 0.0 | 0.0 | 0.0 | 3.3 | 21.6 | 51.8 |
| 145 | 12.7 | 58.4 | 26.6 | 2.3 | 0.0 | 0.0 | 0.0 | 0.0 | 3.3 | 21.1 | 46.0 |
| 150 | 13.4 | 55.8 | 24.7 | 6.1 | 0.0 | 0.0 | 0.0 | 0.0 | 3.1 | 20.9 | 53.0 |

| **Strážnické Pomoraví area – abandoned meander, SPO3 core** | | | | | | | | | | | |
| --- | --- | --- | --- | --- | --- | --- | --- | --- | --- | --- | --- |
| Depth (cm) | Clay (%) | Silt (%) | | Sand (%) | | | | | Grain size parameters | | |
|  |  |  |  | Very fine | Fine | Medium | Coarse | Very coarse |  |  |  |
|  | μm | | | | | | | | | | |
|  | <4 | 4-32 | 32-63 | 63-125 | 125-250 | 250-500 | 500-1000 | 1000-2000 | D_10_ (μm) | D_50_ (μm) | D_90_ (μm) |
| 7 | 15.8 | 75.5 | 8.7 | 0.0 | 0.0 | 0.0 | 0.0 | 0.0 | 2.9 | 14.9 | 30.2 |
| 14 | 14.5 | 74.1 | 11.5 | 0.0 | 0.0 | 0.0 | 0.0 | 0.0 | 3.2 | 16.6 | 32.1 |
| 25 | 13.7 | 75.3 | 11.1 | 0.0 | 0.0 | 0.0 | 0.0 | 0.0 | 3.3 | 16.7 | 31.8 |
| 32 | 16.8 | 74.5 | 8.7 | 0.0 | 0.0 | 0.0 | 0.0 | 0.0 | 2.8 | 14.9 | 30.2 |
| 46 | 12.8 | 73.9 | 13.3 | 0.0 | 0.0 | 0.0 | 0.0 | 0.0 | 3.5 | 17.6 | 33.2 |
| 57 | 15.1 | 73.6 | 11.2 | 0.0 | 0.0 | 0.0 | 0.0 | 0.0 | 3.1 | 16.2 | 31.9 |
| 68 | 16.8 | 74.5 | 8.7 | 0.0 | 0.0 | 0.0 | 0.0 | 0.0 | 2.8 | 15.1 | 30.2 |
| 82 | 14.8 | 75.1 | 10.2 | 0.0 | 0.0 | 0.0 | 0.0 | 0.0 | 3.1 | 15.8 | 31.1 |
| 89 | 13.9 | 72.3 | 13.8 | 0.1 | 0.0 | 0.0 | 0.0 | 0.0 | 3.3 | 17.6 | 33.5 |
| 96 | 19.7 | 62.7 | 9.1 | 8.1 | 0.5 | 0 | 0.0 | 0.0 | 0.6 | 11.9 | 53.7 |
| 102 | 5.7 | 37.1 | 19.6 | 6.3 | 27.5 | 3.8 | 0.0 | 0.0 | 6.1 | 36.9 | 210.3 |
| 108 | 7.9 | 48.2 | 29.8 | 13.2 | 0.9 | 0 | 0.0 | 0.0 | 4.7 | 27.8 | 72.5 |
| 112 | 5.1 | 31.5 | 19.2 | 14.3 | 28.3 | 1.7 | 0.0 | 0.0 | 6.8 | 45.7 | 191.0 |
| 116 | 2.1 | 14.4 | 8.8 | 4.8 | 50.2 | 19.7 | 0.0 | 0.0 | 22.3 | 179.3 | 286.8 |
| 118 | 4.9 | 30.5 | 17.1 | 8.9 | 32.5 | 6.1 | 0.0 | 0.0 | 7.5 | 51.3 | 227.2 |
| 124 | 2.4 | 17.6 | 13.9 | 15.1 | 41.9 | 8.9 | 0.0 | 0.0 | 20.5 | 127.3 | 245.3 |
| 132 | 0.7 | 5.8 | 4.6 | 0.9 | 29.9 | 56.7 | 1.6 | 0.0 | 47.7 | 270.8 | 407.6 |
| 136 | 0.6 | 4.6 | 3.8 | 2.1 | 21.6 | 57.5 | 10.1 | 0.0 | 106.3 | 312.6 | 500.3 |
| 142 | 1.2 | 9.6 | 6.6 | 4.4 | 25.8 | 38.1 | 14.2 | 0.0 | 29.4 | 261.2 | 546.4 |
| 148 | 1.2 | 10.4 | 8.3 | 4.3 | 24.2 | 40.9 | 10.7 | 0.0 | 28.3 | 257.3 | 507.9 |

**Table S8: Results of total organic carbon (TOC) measurements**

| **Bohumín area** | | | | | | **Strážnické Pomoraví area** | | | | | | | |
| --- | --- | --- | --- | --- | --- | --- | --- | --- | --- | --- | --- | --- | --- |
| **Channel bars** | | **Abandoned meander**: cores (depth in cm) | | | | **Channel bars** | | **Abandoned meander**: cores (depth in cm) | | | | | |
| Sample | TOC (%) | CHAL1 | TOC (%) | CHAL2 | TOC (%) | Sample | TOC (%) | SPO1 | TOC (%) | SPO2 | TOC (%) | SPO3 | TOC (%) |
| ODB1 | 4.8 | 3 | 2.6 | 8 | 1.8 | SP1 | 1.4 | 11 | 3.4 | 5 | 4.5 | 4 | 2.9 |
| ODB2 | 4.4 | 10 | 3.6 | 17 | 2.3 | SP2 | 3.2 | 30 | 3.1 | 10 | 5.1 | 18 | 2.5 |
| ODB3 | 7.9 | 17 | 3.5 | 25 | 3.2 | SP3 | 4.1 | 48 | 3.3 | 15 | 3.5 | 36 | 3.1 |
| ODB4 | 5.1 | 24 | 3.1 | 33 | 3.4 | SP4 | 4.2 | 67 | 3.6 | 24 | 2.7 | 53 | 3.1 |
| ODB5 | 9.1 | 31 | 3.4 | 42 | 3.4 | SP5 | 0.1 | 85 | 2.6 | 32 | 2.7 | 71 | 2.1 |
| ODB6 | 1.6 | 38 | 2.9 | 50 | 2.8 | SP6 | 0.1 | 100 | 2.5 | 49 | 2.7 | 100 | 1.1 |
| ODB7 | 4.9 | 45 | 2.9 | 58 | 2.4 | SP7 | 0.1 | 106 | 2.7 | 67 | 2.7 | 104 | 1.7 |
| ODB8 | 7.1 | 52 | 2.6 | 67 | 2.1 | SP8 | 0.1 | 116 | 2.4 | 84 | 2.6 | 110 | 1.8 |
| ODB9 | 5.8 | 59 | 2.8 | 75 | 3.1 | SP9 | 2.9 | 126 | 2.4 | 101 | 3.1 | 120 | 1.9 |
| ODB10 | 9.1 | 66 | 3.3 | 83 | 3.2 | SP10 | 2.4 | 136 | 2.3 | 120 | 2.8 | 130 | 0.3 |
| ODB11 | 5.1 | 72 | 3.4 | 92 | 2.6 | SP11 | 0.2 | 146 | 2.6 | 125 | 2.8 | 140 | 2.2 |
| ODB12 | 0.5 | 79 | 3.4 | 100 | 0.7 | SP12 | 3.8 | 156 | 2.5 | 135 | 3.2 | 150 | 1.2 |
| ODB13 | 4.7 | 86 | 3.1 | 110 | 1.7 | SP13 | 2.2 | 166 | 2.9 | 145 | 3.6 |  |  |
| ODB14 | 5.6 | 90 | 5.7 | 115 | 1.9 | SP14 | 1.9 | 176 | 2.9 | 155 | 3.2 |  |  |
|  |  | 100 | 3.9 | 120 | 2.2 | SP15 | 0.1 | 186 | 1.7 | 165 | 1.1 |  |  |
|  |  |  |  | 125 | 2.3 | SP16 | 0.4 | 196 | 0.6 |  |  |  |  |
|  |  |  |  | 135 | 1.9 |  |  |  |  |  |  |  |  |
|  |  |  |  | 140 | 0.9 |  |  |  |  |  |  |  |  |
|  |  |  |  | 145 | 1.9 |  |  |  |  |  |  |  |  |
|  |  |  |  | 150 | 1.7 |  |  |  |  |  |  |  |  |

**Table S9: Concentrations of PAHs, ratio LMW/HMW and PCBs in channel bars and abandoned meanders**

| **Bohumín area,** < under the limit of quantification | | | | | | | |
| --- | --- | --- | --- | --- | --- | --- | --- |
| **Channel bars** | | | | **Abandoned meander** | | | |
| Sample | Σ PAHs mg/kg | LMW/HMW | Σ PCBs µg/kg | CHAL1 core (depth in cm) | Σ PAHs mg/kg | LMW/HMW | Σ PCBs µg/kg |
| ODB1 | 29.8 | 1.73 | 9.9 | 8 | 19.6 | 0.12 | < |
| ODB2 | 19.7 | 1.77 | 13.1 | 17 | 18.7 | 0.09 | < |
| ODB3 | 72.7 | 1.69 | 24.0 | 25 | 20.0 | 0.09 | < |
| ODB4 | 34.8 | 1.60 | 35.3 | 33 | 17.3 | 0.10 | < |
| ODB5 | 61.2 | 1.64 | 1.0 | 42 | 20.0 | 0.09 | < |
| ODB6 | 8.6 | 1.22 | 10.4 | 50 | 21.0 | 0.12 | < |
| ODB7 | 20.5 | 1.78 | 12.1 | 58 | 28.8 | 0.14 | 239.8 |
| ODB8 | 14.0 | 1.66 | 14.6 | 66 | 30.7 | 0.18 | 54.1 |
| ODB9 | 13.2 | 1.56 | 11.4 | 75 | 16.3 | 0.10 | 113.2 |
| ODB10 | 6.2 | 1.51 | 1.0 | 83 | 18.6 | 0.11 | 222.8 |
| ODB11 | 8.9 | 1.38 | 6.3 | 91 | 21.7 | 0.19 | 83.7 |
| ODB12 | 18.0 | 1.76 | 9.2 | 100 | 15.8 | 0.17 | 6.5 |
| ODB13 | 15.2 | 1.75 | 23.4 | 110 | 18.0 | 0.22 | < |
| ODB14 | 18.1 | 1.61 | 47.6 | 115 | 17.2 | 0.19 | < |
|  |  |  |  | 120 | 25.5 | 0.20 | 221.7 |
|  |  |  |  | 125 | 26.5 | 0.28 | 224.7 |
|  |  |  |  | 135 | 24.1 | 0.22 | 220.0 |
|  |  |  |  | 140 | 746.9 | 0.30 | 278.7 |
|  |  |  |  | 145 | 16.2 | 0.32 | 50.3 |
|  |  |  |  | 150 | 24.7 | 0.27 | 223.2 |
| **Strážnické Pomoraví area** | | | | | | | |
| **Channel bars** | | | | **Abandoned meander** | | | |
| Sample | Σ PAHs mg/kg | LMW/HMW | Σ PCBs µg/kg | SPO2 core (depth in cm) | Σ PAHs mg/kg | LMW/HMW | Σ PCBs µg/kg |
| SP1 | 4.7 | 0.13 | < | 5 | 31.7 | 0.02 | 30.2 |
| SP2 | 13.8 | 0.09 | 3.9 | 10 | 74.5 | 0.01 | 29.9 |
| SP3 | 22.8 | 0.06 | 1.3 | 15 | 59.1 | 0.01 | 77.6 |
| SP4 | 22.5 | 0.05 | 15.1 | 24 | 37.4 | 0.03 | 30.4 |
| SP5 | 1.2 | 0.08 | < | 32 | 27.2 | 0.02 | 30.2 |
| SP6 | 1.5 | 0.12 | < | 49 | 31.2 | 0.02 | 30.2 |
| SP7 | 0.8 | 0.10 | < | 67 | 36.0 | 0.02 | 29.5 |
| SP8 | 3.9 | 0.07 | < | 84 | 33.6 | 0.01 | 107.8 |
| SP9 | 11.2 | 0.08 | 12.3 | 101 | 34.4 | 0.02 | 110.1 |
| SP10 | 12.3 | 0.08 | < | 120 | 44.7 | 0.02 | 109.6 |
| SP11 | 2.0 | 0.04 | < | 125 | 46.9 | 0.02 | 79.5 |
| SP12 | 19.6 | 0.07 | 13.3 | 135 | 44.5 | 0.02 | 217.4 |
| SP13 | 6.6 | 0.11 | < | 145 | 61.4 | 0.02 | 221.7 |
| SP14 | 5.0 | 0.13 | 27.1 | 155 | 50.7 | 0.02 | 77.2 |
| SP15 | 0.2 | 0.29 | 4.6 | 165 | 51.1 | 0.01 | 163.2 |
| SP16 | 3.6 | 0.11 | < |  |  |  |  |

**Table S10: Concentrations of pesticides in channel bars and abandoned meanders**

| **Bohumín channel bars** | | | | | | | | | | | | | | | | | | | | | | | | | | | | | |
| --- | --- | --- | --- | --- | --- | --- | --- | --- | --- | --- | --- | --- | --- | --- | --- | --- | --- | --- | --- | --- | --- | --- | --- | --- | --- | --- | --- | --- | --- |
|  | µg/kg; < under the limit of quantification | | | | | | | | | | | | | | | | | | | | | | | | | | | | |
| Sample | | Ametryn | Atrazine | Atrazine-2-hydroxy | Azoxystrobin | Chloridazon | Chlorotoluron | Chlorpyrifos | Clomazon | Cyproconazol | Diazinon | Difenoconazol | Diflufenican | Diuron | Epoxiconazole | Hexazinone | Metazachlor | Prochloraz | Propiconazole | Simazin-2-hydroxy | Spiroxamine | Tebuconazole | Terbuthylazin | Terbuthylazin-desethyl-2-hydroxy | Terbuthylazine-2-hydroxy | Terbutryn | Prometryn | Chloridazon-desphenyl | Chloridazon-m.-desphenyl |
| ODB1 | | < | 0.1 | 0.9 | 0.3 | 0.2 | 0.2 | 0.4 | 2.7 | 0.2 | 0.1 | 0.4 | 0.8 | 0.4 | 0.7 | 0.2 | 1.5 | 0.4 | 2.2 | 0.2 | 0.3 | 1.5 | 0.3 | 0.2 | 1.0 | 0.7 | < | < | < |
| ODB2 | | 0.2 | 0.1 | 0.8 | 0.3 | 0.2 | 0.2 | 0.5 | 2.0 | 0.2 | 0.1 | 0.4 | 0.5 | 0.3 | 0.6 | 0.2 | 1.5 | 0.3 | 1.6 | 0.2 | 0.4 | 0.9 | 0.2 | 0.2 | 0.8 | 0.9 | < | < | < |
| ODB3 | | 0.2 | 0.1 | 1.0 | 0.3 | 0.2 | 0.2 | 0.6 | 2.2 | 0.2 | 0.1 | 0.4 | 0.8 | 0.3 | 0.6 | 0.2 | 1.5 | 0.3 | 2.0 | 0.2 | 0.5 | 1.4 | 0.2 | 0.2 | 0.9 | 1.3 | < | < | < |
| ODB4 | | 0.2 | 0.1 | 0.9 | 0.3 | 0.3 | 0.3 | 0.6 | 1.3 | 0.2 | 0.1 | 0.4 | 0.9 | 0.3 | 1.0 | 0.2 | 0.8 | 0.4 | 2.0 | 0.2 | 0.4 | 2.3 | 0.3 | 0.3 | 1.1 | 0.9 | < | < | < |
| ODB5 | | 0.2 | 0.0 | 0.3 | 0.2 | < | < | 0.3 | 0.5 | < | < | 0.1 | 0.2 | < | 0.2 | < | 0.2 | 0.1 | 0.6 | 0.2 | 0.3 | 0.2 | 0.1 | 0.2 | 0.3 | 0.1 | < | < | < |
| ODB6 | | 0.2 | 0.1 | 1.2 | 0.2 | 0.1 | < | 0.4 | 1.5 | 0.2 | < | 0.2 | 0.6 | 0.3 | 0.6 | 0.2 | 2.5 | 0.2 | 1.9 | 0.2 | 0.3 | 0.7 | 0.2 | 0.3 | 1.7 | 0.5 | < | < | < |
| ODB7 | | 0.2 | 0.1 | 0.5 | 0.2 | 0.2 | 0.2 | 0.6 | 1.5 | < | < | 0.4 | 0.7 | 0.4 | 0.5 | 0.2 | 1.3 | 0.2 | 3.0 | 0.2 | 0.4 | 1.2 | 0.2 | 0.2 | 0.5 | 1.3 | < | < | < |
| ODB8 | | 0.2 | 0.1 | 1.3 | 0.4 | 0.2 | 0.2 | 0.9 | 3.3 | 0.3 | 0.1 | 0.5 | 1.1 | 0.4 | 0.9 | 0.2 | 1.7 | 0.4 | 2.4 | 0.2 | 0.4 | 2.3 | 0.3 | 0.2 | 1.1 | 1.6 | < | < | < |
| ODB9 | | 0.2 | < | 1.2 | 0.4 | 0.3 | 0.2 | 0.6 | 5.2 | 0.2 | 0.1 | 0.6 | 0.9 | 0.3 | 1.2 | 0.2 | 2.5 | 0.7 | 2.6 | 0.3 | 0.5 | 2.7 | 0.3 | 0.2 | 1.6 | 1.0 | < | < | < |
| ODB10 | | 0.2 | < | 0.3 | 0.1 | < | < | < | 0.3 | < | < | 0.1 | < | < | 0.2 | < | < | 0.1 | 0.6 | 0.2 | 0.3 | 0.2 | 0.1 | < | 0.1 | 0.1 | < | < | < |
| ODB11 | | 0.2 | < | 1.0 | 0.2 | 0.2 | 0.1 | 0.6 | 2.5 | 0.2 | 0.1 | 0.3 | 0.6 | 0.3 | 0.4 | 0.2 | 0.8 | 0.4 | 1.5 | 0.2 | 0.4 | 1.0 | 0.2 | 0.2 | 0.7 | 0.8 | < | < | < |
| ODB12 | | 0.2 | 0.1 | 1.0 | 0.4 | 0.3 | 0.3 | 0.7 | 3.8 | 0.3 | 0.1 | 0.6 | 1.1 | 0.5 | 1.1 | 0.2 | 2.8 | 0.5 | 2.8 | 0.3 | 0.5 | 1.9 | 0.3 | 0.2 | 1.0 | 1.9 | < | < | < |
| ODB13 | | 0.2 | 0.1 | 0.8 | 0.4 | 0.3 | 0.3 | < | 0.3 | 0.2 | 0.1 | 1.1 | 0.5 | 1.9 | 0.2 | 0.2 | < | 0.2 | 3.9 | 0.2 | 0.5 | 1.3 | 0.4 | 0.2 | 1.3 | 6.6 | < | < | < |
| ODB14 | | 0.2 | 0.2 | 0.9 | 0.3 | 0.4 | 0.3 | < | 0.3 | 0.2 | 0.1 | 0.5 | 0.4 | 1.4 | 0.2 | 0.2 | 0.2 | 0.1 | 2.7 | 0.2 | 0.4 | 1.2 | 0.4 | 0.3 | 1.0 | 6.0 | < | < | < |

| **Bohumín abandoned meander, CHAL1 core** | | | | | | | | | | | | | | | | | | | | | | | | | | | | | |
| --- | --- | --- | --- | --- | --- | --- | --- | --- | --- | --- | --- | --- | --- | --- | --- | --- | --- | --- | --- | --- | --- | --- | --- | --- | --- | --- | --- | --- | --- |
|  | µg/kg; < under the limit of quantification | | | | | | | | | | | | | | | | | | | | | | | | | | | | |
| Depth (cm) | | Ametryn | Atrazine | Atrazine-2-hydroxy | Azoxystrobin | Chloridazon | Chlorotoluron | Chlorpyrifos | Clomazon | Cyproconazol | Diazinon | Difenoconazol | Diflufenican | Diuron | Epoxiconazole | Hexazinone | Metazachlor | Prochloraz | Propiconazole | Simazin-2-hydroxy | Spiroxamine | Tebuconazole | Terbuthylazin | Terbuthylazin-desethyl-2-hydroxy | Terbuthylazine-2-hydroxy | Terbutryn | Prometryn | Chloridazon-desphenyl | Chloridazon-m.-desphenyl |
| 8 | | 0.4 | 0.7 | 1.1 | 0.3 | 0.5 | 0.4 | < | < | 0.7 | < | 0.6 | 1.0 | < | 0.7 | < | 0.4 | 0.7 | 1.4 | 0.4 | 0.3 | 1.7 | 0.7 | 0.4 | 1.6 | 1.3 | 0.4 | < | 1.0 |
| 17 | | 0.4 | 0.6 | 1.0 | 0.4 | 0.5 | 0.5 | < | < | < | < | 0.9 | 1.5 | < | 0.8 | < | 0.6 | 1.0 | 2.9 | 0.4 | 0.3 | 3.4 | 0.7 | 0.4 | 1.7 | 1.2 | 0.5 | < | 1.1 |
| 25 | | 0.4 | 0.7 | 1.2 | 0.4 | 0.6 | 0.9 | < | 0.7 | 0.7 | < | 0.9 | 1.6 | < | 0.9 | < | 0.7 | 1.1 | 3.0 | 0.4 | 0.3 | 4.0 | 0.7 | 0.4 | 1.3 | 1.5 | 0.5 | < | 1.2 |
| 33 | | 0.4 | 0.7 | 1.1 | 0.5 | 0.6 | 0.7 | < | 0.8 | 0.6 | < | 0.9 | 1.6 | < | 0.9 | < | 0.7 | 1.0 | 3.4 | 0.4 | 0.3 | 5.7 | 0.8 | 0.4 | 1.8 | 1.7 | 0.5 | < | 1.0 |
| 42 | | < | < | < | < | < | < | < | < | < | < | < | < | < | < | < | < | 0.3 | < | < | < | < | < | < | < | < | < | < | < |
| 50 | | 0.4 | 0.7 | 1.4 | 0.5 | 0.6 | 0.7 | < | < | 0.7 | < | 1.0 | 1.4 | < | 1.0 | < | 0.8 | 0.9 | 1.9 | 0.4 | 0.3 | 4.3 | 0.6 | 0.4 | 2.1 | 1.0 | 0.5 | < | 1.6 |
| 58 | | < | 0.7 | 1.2 | < | 0.5 | 0.2 | < | < | 0.7 | < | 0.4 | 0.9 | < | 0.6 | < | < | 0.4 | 1.2 | 0.4 | < | 0.9 | 0.6 | 0.4 | 2.2 | 0.4 | 0.3 | < | 1.2 |
| 66 | | 0.4 | 0.7 | 1.7 | < | 0.6 | 0.3 | < | < | 0.7 | < | 0.5 | 0.7 | < | < | < | < | 0.7 | 1.9 | 0.4 | < | 1.4 | 0.6 | 0.4 | 2.0 | 0.7 | 0.4 | < | 0.9 |
| 75 | | 0.4 | 0.7 | 1.7 | < | 0.7 | 0.6 | < | < | 0.7 | < | 0.5 | < | 1.4 | < | < | < | 1.0 | 1.6 | 0.4 | < | 1.9 | 0.6 | 0.4 | 2.1 | 1.0 | 0.4 | < | 0.9 |
| 83 | | 0.5 | 0.7 | 1.7 | < | 0.6 | 0.6 | < | < | 0.8 | < | 0.5 | < | 1.3 | < | < | < | 1.2 | 3.2 | 0.4 | 0.2 | 2.1 | 0.6 | 0.4 | 2.0 | 1.0 | 0.4 | < | 0.9 |
| 91 | | 0.5 | 0.7 | 1.6 | < | 0.8 | 0.5 | < | 0.6 | 0.8 | < | < | < | 1.2 | < | < | < | 0.7 | 1.8 | 0.4 | < | 1.4 | 0.6 | 0.3 | 1.4 | 0.6 | 0.4 | < | 0.8 |
| 100 | | 0.4 | 0.7 | 1.0 | < | 0.4 | 0.3 | < | < | 0.7 | < | < | < | < | < | < | < | 0.5 | 0.9 | 0.4 | < | 1.0 | 0.6 | 0.3 | 0.5 | 0.4 | 0.4 | < | 0.5 |
| 110 | | 0.4 | 0.7 | 1.1 | < | 0.6 | 0.5 | < | < | 0.7 | < | 0.4 | < | < | < | < | < | 0.7 | 1.1 | 0.4 | < | 1.3 | 0.6 | 0.3 | 1.1 | 0.5 | 0.4 | < | 0.7 |
| 115 | | 0.4 | 0.7 | 1.4 | < | 0.6 | 0.4 | < | < | 0.7 | < | < | < | < | < | < | < | 0.6 | 1.4 | 0.4 | < | 1.0 | 0.6 | 0.3 | 1.0 | 0.4 | 0.4 | < | 0.7 |
| 120 | | 0.5 | 0.8 | 1.4 | < | 0.6 | 0.6 | < | < | < | 0.6 | < | < | < | < | < | < | 0.6 | 1.4 | 0.3 | < | 1.1 | 0.6 | 0.3 | 0.8 | 0.5 | 0.4 | < | 0.7 |
| 125 | | 0.5 | 0.7 | 1.2 | < | 0.5 | 0.4 | < | 0.7 | < | 0.5 | < | < | < | < | < | < | 0.6 | 1.2 | 0.3 | < | 1.1 | 0.6 | 0.3 | 0.8 | 0.4 | 0.4 | < | 0.7 |
| 135 | | 0.5 | 0.7 | 1.3 | < | 0.6 | 0.5 | < | 0.7 | < | 1.2 | < | < | < | < | < | < | 0.7 | 1.4 | 0.3 | < | 1.3 | 0.6 | 0.3 | 1.0 | 0.5 | 0.4 | < | 0.7 |
| 140 | | 0.4 | 0.7 | 1.0 | < | 0.5 | 0.3 | < | < | < | < | < | < | < | < | < | < | 0.5 | < | 0.3 | < | 0.9 | 0.6 | 0.3 | 0.5 | 0.4 | 0.3 | < | 0.7 |
| 145 | | 0.4 | 0.7 | 0.8 | < | 0.4 | 0.2 | < | < | < | < | < | < | < | < | < | < | 0.4 | < | 0.3 | < | 0.8 | 0.6 | 0.3 | 0.3 | 0.4 | 0.3 | < | 0.5 |
| 150 | | 0.4 | 0.7 | 1.2 | < | 0.6 | 0.7 | < | < | < | < | < | < | < | < | < | < | 0.5 | < | 0.3 | < | 0.9 | 0.5 | 0.3 | 0.7 | 0.4 | 0.4 | < | 0.8 |

| **Strážnice channel bars** | | | | | | | | | | | | | | | | | | | | | | | | | | | | | |
| --- | --- | --- | --- | --- | --- | --- | --- | --- | --- | --- | --- | --- | --- | --- | --- | --- | --- | --- | --- | --- | --- | --- | --- | --- | --- | --- | --- | --- | --- |
|  | µg/kg; < under the limit of quantification | | | | | | | | | | | | | | | | | | | | | | | | | | | | |
| Sample | | Ametryn | Atrazine | Atrazine-2-hydroxy | Azoxystrobin | Chloridazon | Chlorotoluron | Chlorpyrifos | Clomazon | Cyproconazol | Diazinon | Difenoconazol | Diflufenican | Diuron | Epoxiconazole | Hexazinone | Metazachlor | Prochloraz | Propiconazole | Simazin-2-hydroxy | Spiroxamine | Tebuconazole | Terbuthylazin | Terbuthylazin-desethyl-2-hydroxy | Terbuthylazine-2-hydroxy | Terbutryn | Prometryn | Chloridazon-desphenyl | Chloridazon-m.-desphenyl |
| SP1 | | < | < | 0.6 | 0.2 | < | < | < | 0.4 | < | < | 0.2 | 0.3 | < | 0.2 | < | 0.6 | 0.2 | 0.5 | 0.1 | < | 0.7 | 0.2 | < | 0.6 | 0.3 | < | < | 0.3 |
| SP2 | | < | < | 0.6 | 0.4 | 0.2 | 0.1 | < | 0.7 | < | < | 0.2 | 0.6 | < | 0.4 | < | 1.3 | 0.3 | 0.8 | 0.1 | < | 1.2 | 0.3 | 0.1 | 0.8 | 0.4 | < | < | 0.6 |
| SP3 | | < | < | 1.0 | 0.3 | 0.4 | 0.1 | < | 0.9 | < | < | 0.2 | 0.5 | < | 0.4 | < | 2.0 | 0.3 | 0.7 | 0.1 | < | 1.2 | 0.3 | 0.1 | 0.9 | 0.3 | < | < | 0.6 |
| SP4 | | < | < | 0.9 | 0.4 | 0.2 | 0.1 | < | 0.9 | < | < | 0.3 | 0.5 | < | 0.4 | < | 1.8 | 0.4 | 0.7 | 0.1 | < | 1.4 | 0.3 | 0.1 | 0.9 | 0.5 | < | < | 0.7 |
| SP5 | | < | < | < | 0.1 | < | < | < | < | < | < | < | < | < | < | < | < | < | < | < | < | < | < | < | < | < | < | < | < |
| SP6 | | < | < | < | < | < | < | < | < | < | < | < | < | < | < | < | 0.1 | < | < | < | < | < | < | < | < | < | < | < | < |
| SP7 | | < | < | < | < | < | < | < | < | < | < | < | < | < | < | < | < | < | < | < | < | < | < | < | 0.2 | < | < | < | 0.1 |
| SP8 | | < | < | 0.3 | 0.1 | < | < | < | 0.4 | < | < | 0.1 | 0.2 | < | 0.1 | < | 0.1 | 0.1 | 0.3 | 0.1 | < | 0.4 | 0.2 | < | 0.3 | 0.3 | < | < | 0.2 |
| SP9 | | < | < | 0.6 | 0.3 | 0.2 | < | < | 0.7 | < | < | 0.2 | 0.4 | < | 0.3 | < | 1.0 | 0.3 | 0.6 | 0.1 | < | 1.1 | 0.2 | < | 0.7 | 0.3 | < | < | 0.5 |
| SP10 | | < | < | 0.8 | 0.2 | 0.3 | < | < | 0.4 | < | < | 0.2 | 0.4 | < | 0.3 | < | 0.5 | 0.2 | 0.5 | 0.1 | < | 0.9 | 0.2 | < | 0.6 | 0.3 | < | < | 0.4 |
| SP11 | | < | < | < | < | < | < | < | < | < | < | < | < | < | < | < | 0.1 | < | < | < | < | < | < | < | 0.2 | < | < | < | 0.1 |
| SP12 | | < | 0.1 | 0.9 | 0.3 | 0.1 | 0.1 | < | 0.8 | < | < | 0.2 | 0.6 | < | 0.4 | < | 1.6 | 0.4 | 0.8 | 0.1 | < | 1.2 | 0.3 | 0.1 | 0.8 | 0.4 | < | < | 0.7 |
| SP13 | | < | < | 0.7 | 0.2 | 0.2 | < | < | 0.9 | < | < | 0.2 | 0.4 | < | 0.2 | < | 1.0 | 0.2 | 0.5 | 0.1 | < | 0.8 | 0.2 | < | 0.6 | 0.2 | < | < | 0.4 |
| SP14 | | < | < | 0.5 | 0.2 | < | < | < | 0.6 | < | < | 0.2 | 0.3 | < | 0.2 | < | 0.6 | 0.2 | 0.4 | 0.1 | < | 0.6 | 0.2 | < | 0.5 | 0.4 | < | < | 0.3 |
| SP15 | | < | < | < | < | < | < | < | < | < | < | < | < | < | < | < | < | < | < | < | < | < | < | < | < | < | < | < | < |
| SP16 | | < | < | 0.4 | 0.1 | 0.1 | < | < | < | < | < | 0.1 | 0.2 | < | 0.2 | < | 0.1 | 0.1 | < | 0.1 | < | 0.3 | < | < | 0.3 | < | < | < | 0.2 |

| **Strážnice abandoned meander, SPO2 core** | | | | | | | | | | | | | | | | | | | | | | | | | | | | | |
| --- | --- | --- | --- | --- | --- | --- | --- | --- | --- | --- | --- | --- | --- | --- | --- | --- | --- | --- | --- | --- | --- | --- | --- | --- | --- | --- | --- | --- | --- |
|  | µg/kg; < under the limit of quantification | | | | | | | | | | | | | | | | | | | | | | | | | | | | |
| Depth (cm) | | Ametryn | Atrazine | Atrazine-2-hydroxy | Azoxystrobin | Chloridazon | Chlorotoluron | Chlorpyrifos | Clomazon | Cyproconazol | Diazinon | Difenoconazol | Diflufenican | Diuron | Epoxiconazole | Hexazinone | Metazachlor | Prochloraz | Propiconazole | Simazin-2-hydroxy | Spiroxamine | Tebuconazole | Terbuthylazin | Terbuthylazin-desethyl-2-hydroxy | Terbuthylazine-2-hydroxy | Terbutryn | Prometryn | Chloridazon-desphenyl | Chloridazon-m.-desphenyl |
| 5 | | < | < | 1.3 | 0.3 | 0.5 | 0.7 | < | < | < | < | 1.0 | 1.7 | < | 1.1 | < | 0.8 | 0.6 | 1.5 | 0.5 | < | 4.3 | 0.6 | 0.3 | 2.1 | 0.6 | < | 4.5 | 1.0 |
| 10 | | < | < | 1.6 | 0.2 | 0.6 | 0.6 | < | 1.5 | < | < | 0.7 | 1.7 | < | 1.4 | < | 1.7 | 0.5 | 1.3 | 0.5 | < | 3.3 | 0.6 | 0.3 | 2.5 | 0.6 | < | 5.6 | 1.2 |
| 15 | | < | < | 1.4 | < | 0.6 | < | < | < | < | < | < | 1.3 | < | 1.1 | < | 0.1 | 0.3 | < | 0.4 | < | 0.7 | 0.6 | 0.3 | 2.0 | 0.3 | < | < | 1.1 |
| 24 | | < | < | 1.8 | < | 0.7 | < | < | < | < | < | 0.7 | 1.6 | < | 1.3 | < | 0.4 | 0.4 | < | 0.7 | < | 1.2 | 0.7 | 0.5 | 2.5 | 0.4 | < | 3.4 | 0.9 |
| 32 | | < | < | < | < | < | < | < | < | < | < | < | < | < | < | < | 0.1 | < | < | < | < | < | < | < | 0.2 | < | < | < | < |
| 49 | | < | < | < | < | < | < | < | < | < | < | < | < | < | < | < | 0.1 | < | < | < | < | < | < | < | 0.2 | < | < | < | < |
| 67 | | < | < | < | < | < | < | < | < | < | < | < | < | < | < | < | < | 0.2 | < | < | < | < | < | < | 0.2 | < | < | < | < |
| 84 | | < | < | 2.2 | < | 0.6 | < | < | < | < | < | < | 1.4 | < | < | < | < | 0.3 | 0.7 | 0.6 | < | 0.5 | 0.5 | 0.3 | 2.9 | 0.4 | < | 3.8 | 0.8 |
| 101 | | 0.2 | < | 2.0 | < | 0.9 | 0.8 | < | 0.9 | < | < | 0.7 | 1.3 | < | < | < | < | 0.5 | 2.1 | 0.4 | < | 2.0 | 0.5 | 0.3 | 2.8 | 1.2 | 0.2 | 3.9 | 0.8 |
| 120 | | 0.2 | < | 1.7 | < | 0.7 | 0.7 | < | < | < | < | 0.5 | 1.3 | < | < | < | < | 0.4 | 1.7 | 0.4 | < | 1.5 | 0.6 | 0.3 | 2.5 | 0.8 | 0.2 | < | 0.8 |
| 125 | | < | < | < | < | < | < | < | < | < | < | < | < | < | < | < | < | < | < | < | < | < | < | < | 0.2 | < | < | < | < |
| 135 | | 0.3 | < | 2.8 | < | 0.9 | 0.8 | < | 1.1 | < | < | 0.8 | < | < | 1.0 | < | < | 0.9 | 2.4 | 0.5 | < | 2.2 | 0.6 | 0.4 | 4.2 | 1.4 | 0.2 | 4.9 | 0.8 |
| 145 | | 0.2 | < | 2.4 | < | 0.8 | 0.8 | < | < | < | < | 0.7 | 1.3 | < | < | < | < | 0.7 | 2.4 | 0.4 | < | 2.6 | 0.6 | 0.3 | 3.5 | 1.5 | 0.2 | 4.0 | 0.8 |
| 155 | | 0.2 | < | 2.0 | < | 0.8 | 0.8 | < | < | < | < | 0.9 | 1.2 | < | < | < | < | 0.7 | 2.8 | 0.4 | < | 3.2 | 0.5 | 0.3 | 2.6 | 1.2 | 0.2 | 4.3 | 0.8 |
| 165 | | < | < | < | < | < | < | < | < | < | < | < | < | < | < | < | < | < | < | < | < | < | < | < | 0.2 | < | < | < | < |

**Table S11: Concentrations of pharmaceuticals and caffeine**

| **Bohumín channel bars** | | | | | | | | | | | | |
| --- | --- | --- | --- | --- | --- | --- | --- | --- | --- | --- | --- | --- |
|  | µg/kg; < under the limit of quantification | | | | | | | | | | | |
| Sample | 3-hydroxycarbamazepine | Acebutolol | Caffeine | Carbamazepine | Clarithromycin | Diclofenac | Metoprolol | Phenazone | Sulfapyridine | Tramadol | Trimethoprim | N-acetylsulfapyridine |
| ODB 1 | < | 1.2 | 2.2 | 5.7 | < | 0.4 | 1.3 | < | 0.4 | 1.0 | 0.5 | < |
| ODB 2 | 0.1 | 1.1 | 1.4 | 1.1 | 2.6 | 0.3 | 2.3 | < | 0.4 | 0.7 | 0.4 | < |
| ODB 3 | 0.1 | 2.2 | 2.0 | 1.8 | 3.9 | 0.4 | 2.8 | < | 0.4 | 2.0 | 0.5 | < |
| ODB 4 | 0.1 | 1.5 | 5.2 | 2.1 | 1.9 | < | 1.5 | < | 0.4 | 1.2 | 0.4 | < |
| ODB 5 | 0.1 | 0.5 | 1.2 | 0.3 | 1.7 | < | 0.3 | < | 0.2 | 0.4 | 0.2 | < |
| ODB 6 | 0.1 | 0.6 | 1.0 | 1.6 | < | < | 0.9 | < | 0.3 | 1.0 | 0.3 | < |
| ODB 7 | 0.2 | 1.3 | 1.4 | 1.6 | 2.9 | 0.6 | 2.8 | < | 0.8 | 1.3 | 0.4 | < |
| ODB 8 | 0.1 | 1.7 | 2.2 | 1.8 | 2.1 | 0.4 | 2.1 | < | 0.4 | 1.7 | 0.5 | < |
| ODB 9 | 0.1 | 1.2 | 1.4 | 1.7 | 2.8 | 0.5 | 1.6 | < | 0.5 | 1.2 | 0.5 | < |
| ODB10 | 0.1 | 0.7 | 1.3 | 0.3 | 1.7 | < | 0.4 | < | 0.2 | 0.5 | 0.2 | < |
| ODB 11 | 0.1 | 0.7 | 1.2 | 0.5 | 2.0 | < | 0.6 | < | 0.4 | 0.5 | 0.4 | < |
| ODB 12 | 0.1 | 1.4 | 1.7 | 1.8 | 1.4 | 0.6 | 2.5 | < | 0.5 | 1.4 | 0.5 | < |
| OB 13 | 0.3 | 1.5 | 0.9 | 4.0 | 3.6 | 1.4 | 4.8 | 0.3 | 1.2 | 4.3 | 0.5 | < |
| OB 14 | 0.2 | 1.4 | 0.7 | 3.1 | 3.0 | 1.0 | 4.0 | 0.3 | 1.0 | 4.3 | 0.4 | < |

| **Bohumín abandoned meander, CHAL1 core** | | | | | | | | | | | | |
| --- | --- | --- | --- | --- | --- | --- | --- | --- | --- | --- | --- | --- |
|  | µg/kg; < under the limit of quantification | | | | | | | | | | | |
| Depth (cm) | 3-hydroxycarbamazepine | Acebutolol | Caffeine | Carbamazepine | Clarithromycin | Diclofenac | Metoprolol | Phenazone | Sulfapyridine | Tramadol | Trimethoprim | N-acetylsulfapyridine |
| 8 | 0.4 | 0.6 | 2.2 | 2.0 | 0.5 | 0.0 | 1.1 | < | 0.3 | 0.3 | < | < |
| 17 | 0.4 | 0.9 | 2.7 | 2.1 | 0.2 | 0.0 | 2.6 | < | 0.3 | 0.6 | < | < |
| 25 | 0.4 | 0.9 | 3.3 | 2.4 | 0.2 | 0.0 | 3.8 | < | 0.4 | 0.6 | < | < |
| 33 | 0.3 | 0.7 | 3.6 | 2.8 | < | < | 4.2 | < | 0.3 | 0.7 | < | < |
| 42 | < | < | < | < | < | < | < | < | < | < | < | < |
| 50 | 0.4 | 1.0 | 2.8 | 2.2 | 0.3 | 3.1 | 2.5 | < | 0.3 | 0.3 | < | < |
| 58 | 0.3 | 0.4 | 5.1 | 1.6 | 0.4 | < | 1.2 | < | 0.3 | 0.2 | < | < |
| 66 | 0.3 | 0.4 | 3.6 | 1.6 | 0.0 | < | 2.1 | < | 0.3 | 0.1 | < | < |
| 75 | 0.5 | 0.6 | 4.0 | 3.0 | 0.0 | 5.0 | 1.3 | < | 1.0 | 0.1 | < | < |
| 83 | 0.7 | 1.5 | 7.0 | 4.8 | 0.2 | 3.1 | 4.0 | < | 1.0 | 0.2 | < | < |
| 91 | 0.6 | 0.6 | 3.6 | 2.8 | 0.0 | 2.9 | 1.2 | < | 0.7 | 0.1 | < | < |
| 100 | 0.4 | 0.9 | 5.1 | 0.9 | 0.2 | < | 2.6 | < | 0.5 | 0.1 | < | < |
| 110 | 0.4 | 0.5 | 2.9 | 1.4 | 0.3 | < | 0.9 | < | 0.5 | 0.1 | < | < |
| 115 | 0.4 | 0.6 | 10.2 | 0.9 | 0.2 | < | 1.0 | < | 0.5 | 0.1 | < | < |
| 120 | 0.4 | 0.5 | 10.0 | 1.0 | 0.0 | < | 0.5 | < | 0.5 | 0.1 | < | < |
| 125 | 0.4 | 0.5 | 6.2 | 0.9 | 0.0 | < | 0.5 | < | 0.5 | 0.1 | < | < |
| 135 | 0.4 | 0.6 | 5.2 | 1.3 | 0.0 | 3.6 | 1.0 | < | 0.6 | 0.1 | < | < |
| 140 | 0.3 | 0.5 | 5.3 | 0.5 | 0.0 | < | 0.5 | < | 0.4 | 0.1 | < | < |
| 145 | 0.3 | 0.5 | 22.5 | 0.4 | 0.0 | < | 0.8 | < | 0.3 | 0.1 | < | < |
| 150 | 0.4 | 0.4 | 5.8 | 0.9 | 0.0 | 8.8 | 1.0 | < | 0.5 | 0.1 | < | < |

| **Strážnice channel bars** | | | | | | | | | | | | |
| --- | --- | --- | --- | --- | --- | --- | --- | --- | --- | --- | --- | --- |
|  | µg/kg; < under the limit of quantification | | | | | | | | | | | |
| Ssmple | 3-hydroxycarbamazepine | Acebutolol | Caffeine | Carbamazepine | Clarithromycin | Diclofenac | Metoprolol | Phenazone | Sulfapyridine | Tramadol | Trimethoprim | N-acetylsulfapyridine |
| SP1 | < | 0.7 | 0.2 | 0.3 | 1.3 | 0.4 | < | < | 0.3 | < | 0.2 | < |
| SP2 | < | 0.9 | 0.1 | 0.4 | 1.7 | 0.5 | < | < | 0.7 | < | 0.3 | 0.1 |
| SP3 | < | 0.9 | 0.1 | 0.5 | 1.8 | 0.1 | < | < | 0.8 | < | 0.3 | 0.1 |
| SP4 | < | 1.1 | 0.0 | 0.6 | 2.7 | 0.6 | < | < | 1.1 | < | 0.4 | 0.1 |
| SP5 | < | 0.4 | 0.0 | 0.1 | 0.5 | < | < | < | < | < | < | < |
| SP6 | < | 0.3 | 0.0 | 0.1 | 0.6 | < | < | < | < | < | < | < |
| SP7 | < | 0.4 | 0.6 | 0.1 | 0.6 | 0.1 | < | < | < | < | < | < |
| SP8 | < | 0.6 | 0.5 | 0.2 | 0.8 | 0.1 | < | < | 0.2 | < | 0.2 | < |
| SP9 | < | 0.8 | < | 0.5 | 2.9 | 0.3 | < | < | 0.6 | < | 0.3 | 0.1 |
| SP10 | < | 0.7 | 0.2 | 0.4 | 1.5 | 0.2 | < | < | 0.4 | < | 0.3 | 0.1 |
| SP11 | < | 0.3 | 1.5 | 0.1 | 0.4 | < | < | < | < | < | < | < |
| SP12 | < | 0.9 | 0.1 | 0.5 | 2.6 | 0.1 | < | < | 0.8 | < | 0.4 | 0.1 |
| SP13 | < | 0.7 | 0.2 | 0.3 | 2.3 | 0.3 | < | < | 0.6 | < | 0.3 | 0.1 |
| SP14 | < | 0.7 | 0.1 | 0.3 | 2.0 | 0.1 | < | < | 0.4 | < | 0.2 | < |
| SP15 | < | 0.3 | < | 0.1 | 0.4 | < | < | < | < | < | 0.1 | < |
| SP16 | < | 0.5 | < | 0.2 | 0.8 | 0.2 | < | < | 0.1 | < | 0.2 | < |

| **Strážnice abandoned meander, SPO2 core** | | | | | | | | | | | | |
| --- | --- | --- | --- | --- | --- | --- | --- | --- | --- | --- | --- | --- |
|  | µg/kg; < under the limit of quantification | | | | | | | | | | | |
| Depth (cm) | 3-hydroxycarbamazepine | Acebutolol | Caffeine | Carbamazepine | Clarithromycin | Diclofenac | Metoprolol | Phenazone | Sulfapyridine | Tramadol | Trimethoprim | N-acetylsulfapyridine |
| 5 | < | 0.5 | 2.8 | 0.5 | 0.0 | 37.6 | < | < | 0.9 | 0.4 | < | < |
| 10 | < | 0.5 | 3.5 | 0.7 | 0.7 | 23.3 | < | < | 0.6 | 0.6 | < | < |
| 15 | < | 0.0 | 2.7 | 0.6 | 0.8 | 12.3 | < | < | 0.5 | 0.4 | < | < |
| 24 | < | 0.4 | 12.0 | 0.5 | 1.1 | 4.4 | < | < | 0.7 | 0.5 | < | < |
| 32 | < | < | < | < | < | < | < | < | < | < | < | < |
| 49 | < | < | < | < | < | < | < | < | < | < | < | < |
| 67 | < | < | < | < | < | < | < | < | < | < | < | < |
| 84 | < | < | 1.8 | 0.6 | 0.8 | 0 | < | < | 0.5 | < | < | < |
| 101 | 0.2 | 0.5 | 57.9 | 0.9 | 0 | 1.30 | < | < | 1.9 | < | < | < |
| 120 | 0.2 | 0.4 | 4.3 | 0.8 | 0.7 | 0 | < | < | 1.3 | < | < | < |
| 125 | < | < | < | < | < | < | < | < | < | < | < | < |
| 135 | 0.3 | 0.4 | 2.6 | 0.7 | 0 | 3.02 | < | < | 1.5 | < | < | < |
| 145 | 0.3 | 0.4 | 2.4 | 0.8 | 0 | 3.80 | < | < | 1.6 | < | < | < |
| 155 | 0.3 | 0.4 | 1.1 | 0.7 | 0 | 2.63 | < | < | 1.5 | < | < | < |
| 165 | < | < | < | < | < | < | < | < | 0.4 | < | < | < |
